# Design of new disubstituted imidazo[1,2-*b*]pyridazine derivatives as selective Haspin inhibitors. Synthesis, binding mode and anticancer biological evaluation

**DOI:** 10.1080/14756366.2020.1825408

**Published:** 2020-10-12

**Authors:** Jonathan Elie, Omid Feizbakhsh, Nathalie Desban, Béatrice Josselin, Blandine Baratte, Amandine Bescond, Julien Duez, Xavier Fant, Stéphane Bach, Dominique Marie, Matthieu Place, Sami Ben Salah, Agnes Chartier, Sabine Berteina-Raboin, Apirat Chaikuad, Stefan Knapp, Fabrice Carles, Pascal Bonnet, Frédéric Buron, Sylvain Routier, Sandrine Ruchaud

**Affiliations:** a Institut de Chimie Organique et Analytique, Université d’Orléans, UMR CNRS 7311, Orléans Cedex 2, France; b Sorbonne Université/CNRS UMR8227, Station Biologique, Roscoff cedex, France; c Sorbonne Université/CNRS FR2424, Plateforme de criblage KISSf (Kinase Inhibitor Specialized Screening facility) Station Biologique, Roscoff cedex, France; d Sorbonne Université/CNRS UMR7144, Station Biologique, Roscoff cedex, France; e Institute for Pharmaceutical Chemistry, Johann Wolfgang Goethe University, Frankfurt am Main, Germany; f Structure Genomics Consortium, Johann Wolfgang Goethe University, Buchmann Institute for Molecular Life Sciences, Frankfurt am Main, Germany

**Keywords:** Imidazopyridazine, Haspin kinase, co-crystallisation and docking, cellular effects, 3D spheroids

## Abstract

Haspin is a mitotic protein kinase required for proper cell division by modulating Aurora B kinase localisation and activity as well as histone phosphorylation. Here a series of imidazopyridazines based on the CHR-6494 and Structure Activity Relationship was established. An assessment of the inhibitory activity of the lead structures on human Haspin and several other protein kinases is presented. The lead structure was rapidly optimised using a combination of crystal structures and effective docking models, with the best inhibitors exhibiting potent inhibitory activity on Haspin with IC_50_ between 6 and 100 nM *in vitro*. The developed inhibitors displayed anti-proliferative properties against various human cancer cell lines in 2D and spheroid cultures and significantly inhibited the migration ability of osteosarcoma U-2 OS cells. Notably, we show that our lead compounds are powerful Haspin inhibitors in human cells, and did not block G2/M cell cycle transition due to improved selectivity against CDK1/CyclinB.

## Graphical Abstract

**Figure:**
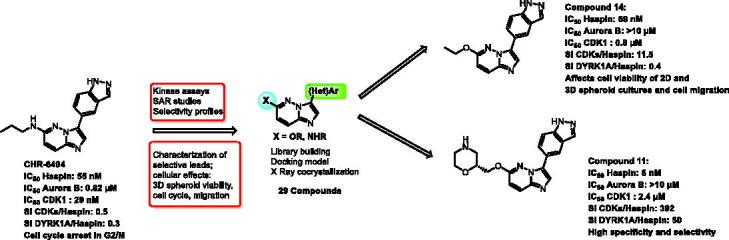

## Introduction

The deregulation of protein phosphorylation is directly responsible for the pathogenesis of several diseases and protein kinases are therefore considered as major drug targets. Specific targeting of kinases that are essential for unwanted cancerous cells is therefore an important step towards therapy. The search for pharmacological kinase inhibitors has become a major approach to discover new therapeutic agents. The most commonly used conventional anticancer agents such as taxanes and vinca alkaloids aim at impairing the ability of the cancerous cell to divide by targeting the microtubule cytoskeleton leading to mitotic arrest and ultimately cell death^1^. Nowadays, more specific ways disturbing cell division are being explored by the pharmaceutical community that involve targeting essential mitotic protein kinases whose expression or activity are found deregulated in many cancers^2–5^.

Aurora B kinase controls many aspects of mitosis, including the correction of microtubule-kinetochore attachment errors and cytokinesis^6^. The deregulation of its activity or expression is often associated with genomic instability and aneuploidy, commonly observed in a vast majority of solid tumours and hematological malignancies^7^. In 2013, more than a dozen Aurora kinase inhibitors were tested in phase I clinical trials^8^. Despite recent advances, the effectiveness of Aurora B kinase inhibitors has so far been limited by the development of resistance and side effects^9^. It is therefore of great interest to develop new strategies targeting Aurora B indirectly through its upstream natural regulators. Many other protein kinases control Aurora B activity directly or indirectly, making them potential therapeutic targets^10–12^. Amongst them, the protein kinase Haspin (also known as GSG2, germ cell associated 2) which has emerged as a key regulator of Aurora B functions. It has been shown to act upstream of Aurora B, regulating both its centromeric localisation and activity^13–17^. It also plays several key roles during mitosis both in maintaining chromatin and centromeric cohesion and spindle pole structures^13^^,^^18–20^. Haspin phosphorylates Histone H3 on threonine 3 (H3T3ph) at mitosis leading to the recruitment and clustering of Aurora B to the centromere^21–23^. This clustering is essential for Aurora B kinase activation, which in return phosphorylates and activates Haspin, creating a positive feedback loop between the two kinases^16^. Haspin kinase appears essential for mitosis as its depletion or inhibition results in cell death caused by mitotic catastrophe^24^^,^^25^. Additionally, Haspin kinase is overexpressed in several malignancies such as Burkitt’s lymphoma, B cell chronic lymphocytic leukaemia and pancreatic cancers^26–29^. Hence, Haspin represents an interesting target for cancer therapy.

**Figure 1.**
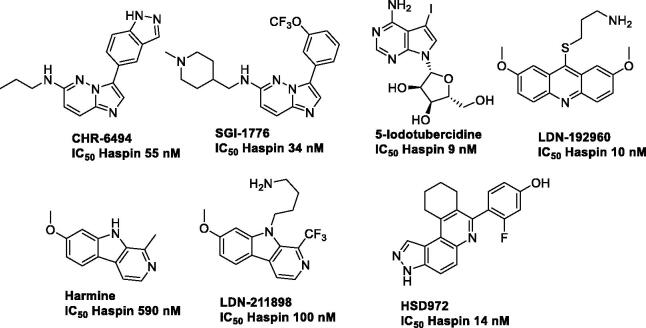
Example of Haspin inhibitors.

Haspin has atypical structural features that are unique to this kinase, increasing the likelihood of identifying specific inhibitors that may result in fewer off-target effects^30^^,^^31^. There are currently a few Haspin inhibitors described in the literature (Figure 1): bicyclic heterocyclic structures such as imidazopyridazine CHR-6494 from Chroma Biotech or SGI-1776^24^^,^^32^; the nucleotide like 5-iodotubercidine^13^^,^^17^ and finally methoxylated fused tricyclic derivatives containing an acridine (LDN-192960) or a β-carboline such as in harmine or LDN-211898^17^^,^^33^^,^^34^. More recently, 3*H*-pyrazolo[4,3-*f*]quinolone as HSD972^35^, and natural products^29^^,^^36^ were additionally reported.

The heterocyclic derivative CHR-6494 is active both *in vitro*, on tumour cells^24^^,^^37^ and *in vivo* as it reduced angiogenesis and tumour growth on nude mice xenografts of HCT-116 cells. CHR-6494 affects also cell viability and mobility of several melanoma cancer lines when used alone or in combination with the Mitogen-activated protein kinase kinase (MEK) inhibitor Trametinib which acts synergistically^37^. However, the compound displays poor selectivity against other kinases but the structure offers an attractive starting point for optimisation.

**Figure 2.**
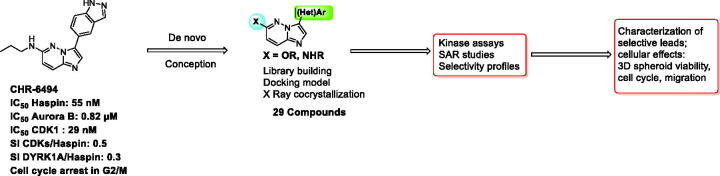
Presented work.

In this study, we report the development of novel imidazopyrimidine derivatives that display improved potency towards Haspin and improved selectivity (Figure 2). Synthesis was fully optimised and modulations were performed in parallel with docking studies. To guide the design of new inhibitors, we solved a high-resolution crystal structure of an early derivative with Haspin. The structure confirmed the expected binding mode interacting with the hinge of the kinase in an adenosine triphosphate (ATP) competitive way. For ligand optimisation, further docking experiments were performed using this structural model.

Structure activity relationships were established and selectivity was assessed using a representative kinase panel. Several cellular studies were performed to demonstrate the specific mode of action of newly synthesised derivatives. Very promising novel leads were obtained that exhibited anti-proliferative properties against various human cancer cell lines grown in 2D and 3D spheroid cell cultures and significantly inhibited the migration ability of osteosarcoma U-2 OS cells.

## Results and discussion

### Chemistry

**Scheme 1.**
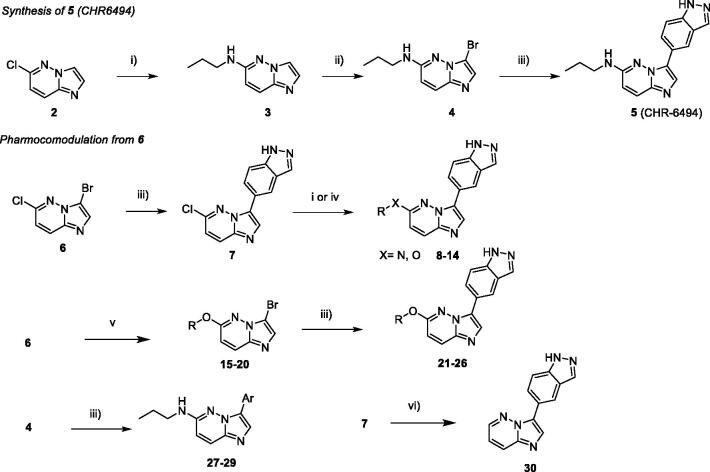
Reagents and conditions: i) HNRR' (5.0 equiv.), NMP, M.W., 180 °C, 1 h; ii) NBS (1.0 equiv.), ACN, r.t. 1 h; iii) ArB(OH)_2_ or ArB(Pin)_2_, Na_2_CO_3_, Pd(PPh_3_)_4_ (0.1 equiv.), Dioxane/H_2_O (9/1), M.W., 150 °C, 1h30. iv) ROH, NaH, NMP, M.W., 180 °C, 1 h; v) ROH, NaH, THF, r.t., 1–5 h; vi) HCO_2_H, Et_3_N, Pd(OAc)_2_ (0.1 equiv.), Xantphos (0.2 equiv.), THF, M.W., 150 °C, 15 min.

First, we developed an efficient synthesis of the CHR-6494 derivative in three steps as this compound was not commercially available at the beginning of this work. The first step consisted of a nucleophilic aromatic substitution (S*_N_*Ar) in position C-6 with propylamine in *N*-methyl-2-pyrrolidone (NMP) under microwave irradiation which furnished compound **3** in 86% yield. Furthermore, a region-selective bromination of compound **3** in the presence of *N*-bromosuccinimide (NBS) led to compound **4** in quantitative yield. The displacement of this halogen was carried out by a Suzuki–Miyaura cross coupling, using 1*H*-indazole-5-boronic acid as partner, in presence of caesium carbonate as base in a mixture of ethanol/water as solvent under microwave irradiation to give compound **5** in the moderate yield of 28% (Scheme 1).

This linear synthetic pathway prompted us to develop a versatile skeleton to explore molecular diversity in C-3 and C-6 positions. We focussed our attention on a bis-halogenated platform **6,** which can be regio-selectively functionalised in C-3 and C-6 positions (Scheme 1) by chlorine and bromine discrimination^38^. However, the method suffered in our hands from long reaction times under thermal conditions, leading to purification difficulties and low yields. Fortunately, the lack of reactivity was fully circumvented using microwave activation, which improved kinetic parameters of the reaction and significantly increased the yield during both palladium cross coupling and S*_N_*Ar reactions^39–41^.

A direct regio-selective Suzuki cross-coupling reaction was first performed with 1*H*-indazole-5-boronic acid under microwave irradiation resulting in a satisfactory yield of compound **7** (61%), which was efficient in comparison to the use of conventional reaction conditions (around 10%). In a second step, starting from compound **7,** S*_N_*Ar occurred in C-6 using various nucleophiles under microwave irradiation and afforded the attempted compounds **8–14** in moderate yields due to the presence of the indazole moiety. Several primary or secondary aliphatic amines were successfully introduced by nucleophilic substitution. An attempted reaction with amino alcohols led only to nitrogen reactions. Condensation of the alcohols required the addition of a base enhancing reactivity for completion of the reaction at a lower temperature in reaction times of only 10 min.

To pursue the building of a focussed library around this scaffold, we modified the synthetic pathway due to the presence of the indazole moiety which led to some limitations such as low solubility or reactivity. We also switched the two steps and began by the S*_N_*Ar, with amines or various chiral alcohols in presence of NaH as base which furnished compounds **15–20** in a moderate to high yields (Table 1).

**Table 1.**
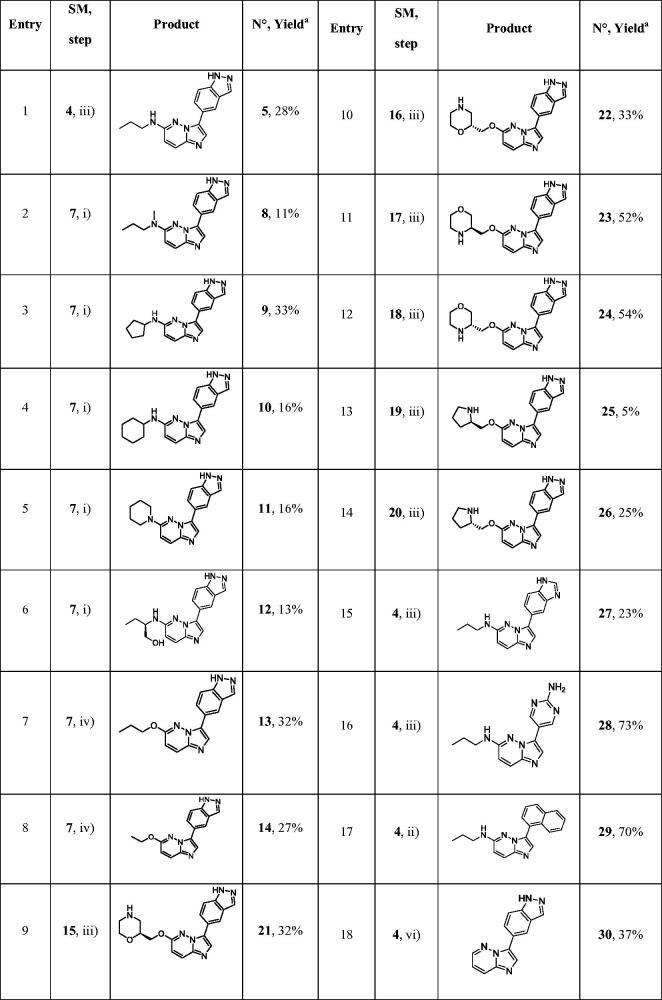
Structure of synthesised derivatives and yields.

SM: starting material.

^a^ Yields are indicated in isolated products.

Next, the Suzuki reaction was performed using indazole boronic acid to furnish compounds **21–26** in moderate yields. Furthermore, benzimidazole and 2-aminopyrimidine as indazole isosters were introduced on bromo derivative **4** to give compounds **27** and **28** in 23% and 73% yield, respectively. We also introduced on compound **4** a naphthyl moiety instead of indazole, to generate derivative **29** with a very satisfactory yield. Without any donor/acceptor hydrogen bond system, this compound was unable to interact with the Haspin hinge region and served as a negative control for biochemical and biological analysis. To complete the construction of the library, we performed the reduction of the C5-Cl bond with a palladium catalyst and microwave irradiation to give the desired product **30** in 37% of yield (Table 1).

### Kinase assays

We tested the inhibitory activity of the 18 synthesised compounds on recombinant *Hs*Haspin activity compared to the reference compound CHR-6494 **5** (Table 2) with the main objective of obtaining more selective Haspin inhibitors and of being able to assess the potency of a selective inhibitor in cellular assays with reduced interference of off-targets.

**Table 2.** IC_50_ (µM) of the imidazopyridazine derivatives on various protein kinases (in bold for haspin kinase). For selected compounds, the selectivity index for each kinase vs Haspin is shown in parentheses.




As expected, compounds without any heteroatom attached to the imidazopyridazine core in C-6 (compound **30**) or lacking the indazole part in C-3 (compounds **27–29**), lost their activity for Haspin. It was evidence that these two elements play a crucial role maintaining potent inhibitory activity and that they define the key pharmacophore. Modulation of the propyl amine in C-6 led to variation in Haspin inhibition. Methylation of the NH group confirmed that the labile proton was not essential (compound **8**). Cycloalkylamines as well as piperidine were used as substituents in C-6 without any significant changes in activity (compounds **9**–**11**). The cyclopentyl group seemed preferred, with an improved activity for compound **9** at IC_50_ = 31 nM. Finally, a second hydrophilic function was introduced on the small amino alkyl side chain in compound **12** and resulted in activity that remained identical to that of other derivatives of this subfamily.

While switching the aminopropyl group for propyloxy in C-6 proved disappointing (compound **13**), the ethoxyl residue was very well tolerated as derivative **14** exhibited good Haspin inhibition with an IC_50_ = 69 nM. The best results were obtained when the C-6 alkyloxy group was substituted by heteroalkyl cycles. With prolinol derivatives, the chiral compounds **25** (R) and **26** (S) were found to be very active with a preference for the S configuration. A real activity improvement was achieved by switching the pyrrolidine for a morpholine (compounds **21**–**24**). The position of the heteroatom had a real impact. Interaction with the kinase active site was favoured when the two electron rich oxygen atoms, which are strong H bond acceptors, were located close to each other. This behaviour was confirmed by the IC_50_ of derivatives **21** and **22** (6 and 12 nM, respectively) which were more active than **23** and **24** (25 and 20 nM, respectively). Finally, the (S) derivative **21** and its enantiomer **22** are to our knowledge the most active Haspin inhibitors in the imidazopyrimidazine series ever reported and join the short list of very potent Haspin inhibitors.

The selectivity of each derivative was quantified on several other kinases. CHR-6494 (**5)** appeared in our hands as a pan kinase inhibitor with moderate selectivity and was surprisingly more active on CDK9 and DYRK1A than on Haspin (Table 2). For the developed imidazopyridazine library, the selectivity *vs* Aurora B was excellent, as the newly designed Haspin inhibitors did not inhibit this kinase at 1 µM. Noteworthy, 12 derivatives did not affect this enzyme at 10 µM (compounds **11**–**14**, **21**–**25**, **28**, **30**). CDK2 and CDK5 inhibition was fully enhanced in the case of C-6-O substituted molecules (compounds **13**,**14**, **21**–**24**). The highest selectivity for CDK9 and DYRK1A occurred with the morpholino containing derivatives (compounds **21**–**24**), which exhibited a (sub)micromolar IC_50_ range on both kinases. The best selectivity was clearly achieved with **21** (IC_50_ Haspin = 6 nM) with selectivity for CDK2, 5, 9 and DYRK1A of 716, 150, 28 and 50-fold, respectively. Altogether, these results showed that our chemical series displayed increased efficacy and selectivity.

Values are IC_50_ expressed in µM and calculated from dose–response curves (each point on the curves was performed in triplicate). Selectivity indexes (SI; in brackets) are calculated as follows: SI = IC_50_ kinase X/IC_50_ Haspin. ND: not determined. *IC_50_ values obtained using the ADP-Glo methodology (see Experimental section, Supplementary Material).

### Binding mode and molecular modelling studies

**Figure 3.**
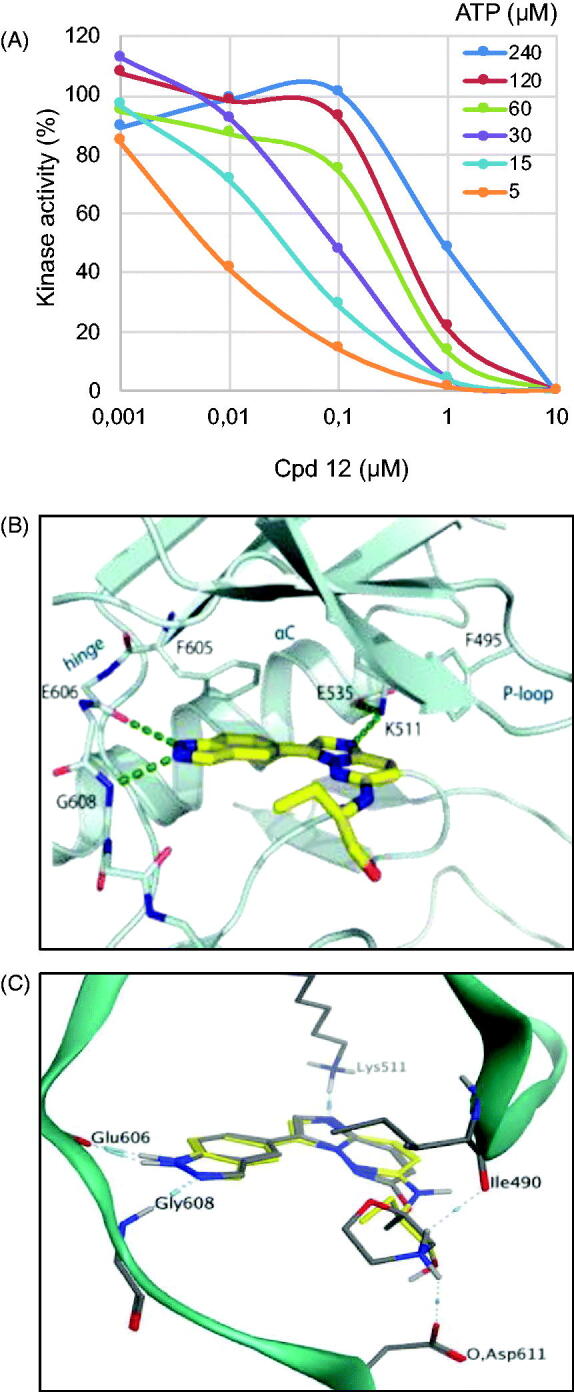
Binding mode of selected compounds with Haspin. (A) ATP competition assay with compound **12**. (B) Crystal structure of Haspin with compound **12**. The inhibitor is displayed in stick representation with yellow carbon atoms, and the key interactions with the kinase ATP binding site are shown. (C) Superimposition of the binding mode of compounds **12** (yellow carbon atoms) and **21** (grey carbon atoms) in Haspin active site. The three-letter amino acid code and residue number are labelled next to each side chain.

We first conducted an ATP competition assay with compound **12** on Haspin kinase activity (Figure 3(A)). We tested an ATP concentration range from 5 to 240 µM on compound **12** concentrations ranging from 0.001 to 10 µm. Our data clearly show the competition between compound **12** and ATP, as the calculated IC_50_ increased from 6 nM at 5 µM ATP to 950 nM at 240 µM ATP.

We next determined the co-crystal structure of Haspin in complex with derivative **12** to determine the binding mode of the inhibitor within the kinase (Figure 3(B)). The inhibitor adopted a planar conformation, positioning the indazole moiety for hydrogen bonds to the hinge region. This orientation resulted in the imidazopyridazine group protruding further in the pocket, interacting with the catalytic lysine Lys511. The amino alkyl side chain decoration tucked beneath the β1 and β2^42^, and occupied the space towards the solvent exposed region of the pocket. This binding mode of compound **12** demonstrated shape complementarity to the ATP-binding pocket of the kinase, thus explaining the good potency of the inhibitor. These results also strongly suggest that our compounds behave as type I kinase inhibitors.

Derivative **21** was docked into the active site of Haspin to understand key interactions with the protein residues. Docking experiments showed that the binding mode of inhibitor **21** was conserved in the ATP active site and formed similar hydrogen bond interactions with the hinge region and catalytic lysine Lys511 to compound **12** (Figure 3(C)). Interestingly, the morpholine group made two additional hydrogen bond interactions between the protonated nitrogen and the oxygen of the backbone carbonyl of isoleucine Ile490 and the carboxyl group of the side chain of aspartic acid Asp611. These extra hydrogen bonds could explain the strong potency of inhibitor **21** compared to compound **12** or CHR-6494 **5.** In addition, this interaction can be seen as a salt bridge between the ammonium of the morpholine group and the carboxylate anion group of the side chain of aspartic acid Asp611. The difference of activity between CHR-6494 **5** and inhibitor **21** towards DYRK1A could be explained by the lack of one hydrogen bond acceptor or a lack of one salt bridge, since the position of Asp611 in Haspin corresponds to asparagine Asn234 in DYRK1A. Moreover, the NH_2_ side chain of Asn234 may be exposed to the protonated nitrogen, thus forming an unfavourable interaction^43^.

### Cellular effects

#### Cell viability

We subsequently tested the effects of selected compounds on the cell viability of various highly proliferative cancerous cell lines (Table 3) from osteosarcoma (U-2 OS), colorectal cancer (HCT116), breast cancer (HBL100) and neuroblastoma (SH-SY5Y) as well as non-cancerous retinal fibroblast RPE-1 immortalised with hTERT, cultured under a conventional monolayer (2-Dimensions) format.

**Table 3.**
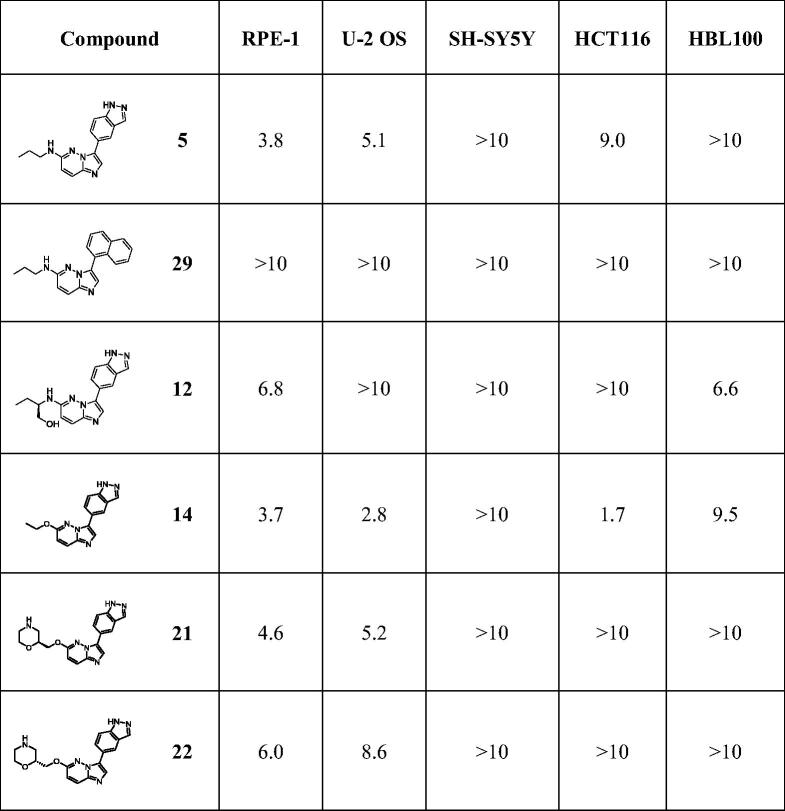
Effects on cell viability.

Cell viability was assessed after 48 h treatment with selected compounds in a dose-dependent manner (Table 3). Among cancerous cell lines, U-2 OS appeared to be the most sensitive one, responding to most compounds apart from compound **12** and our negative control **29** with EC_50_ ranging from 2.8 µM for derivative **14** to 8.6 µM for compound **22**. Compounds were generally more efficient at inhibiting the viability of normal RPE-1 cells than the other cells of cancerous origin. None of the compounds appeared to affect the viability of SH-SY5Y cells and only derivatives **12** and **14** slightly affected HBL100 cells (EC_50_ of 6.6 and 9.5 µM respectively). The negative compound **29** did not impact any of the tested cell lines (all EC_50_ >10 µM). Amongst the selected compounds, **14** appeared the most active one, affecting all cell lines apart from SH-SY5Y. This compound displayed the strongest impact on the viability of U-2 OS and HCT116 cells with EC_50_ of 2.8 µM and 1.7 µM, respectively.

Cells were incubated with increasing doses of each compound for 48 h. Cell viability was determined by MTS assay and EC_50_ (µM) were calculated from the dose–response curves of each compound on the indicated human cell lines (each point made in triplicate).

#### Effects on 3D spheroids

**Figure 4.**
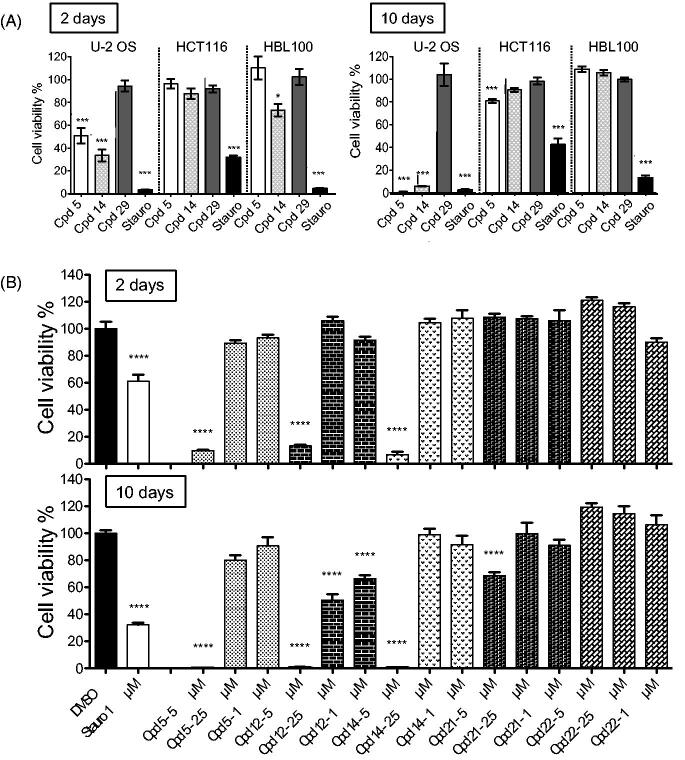
Effects of compounds on spheroid viability. (A) Cell viability in spheroids from HCT116, HBL100 and U-2 OS cells was measured after 2 and 10 days of treatment with DMSO, staurosporine (1 µM) or compounds **5**, **14**, **29** at 5 µM, at a single dose on day 0. Cell viability was expressed in percentage of the DMSO control set at 100%. Data were acquired in triplicates, results are mean ± SEM, ***p ≤ 0.001, *p ≤ 0.05 (two-tailed unpaired *t*-test). (B) U-2 OS spheroids were treated with 5, 2.5 or 1 µM of compounds **5**, **12**, **14**, **21** or **22**, or with 1 µM of staurosporine or DMSO, at a single dose on day 0. Cell viability was measured after 2 and 10 days and expressed in percentage of the DMSO control set at 100%. Data were acquired in triplicates; graphs represent the mean of 2 independent experiments. Results are mean ± SEM, ****p ≤ 0.0001 (two-tailed unpaired *t*-test).

It is known that the impact of drugs on cell division may change markedly depending upon environmental conditions. Therefore, in order to gain in physiological predictivity^44^, the anti-proliferative activity of derivative **14** was further addressed on multicellular spheroids (Figure 4), still using compounds **5** and **29** as reference and negative control respectively. As RPE-1 and SH-SY5Y cells failed to form spheroids, U-2 OS, HCT116 and HBL100 cell lines were used for this approach.

A 48-h exposure with 5 µM of compound **5** triggered a significant decrease in U-2 OS spheroid viability (49%), without any significant effect on that of HCT116 and HBL-100 cells (Figure 4(A), left panel). Notably, derivative **14** impacted U-2 OS spheroid viability slightly more (33%) and showed an additional – rather moderate – activity on HBL-100 spheroids (73%). These results demonstrated that compound **14**, compared to **5**, had similar or slightly stronger effects on U-2 OS spheroid viability and confirmed our results on 2D culture cell viability. After 10-day exposure, the effect on U-2 OS spheroid viability was markedly increased with both compounds while HCT116 and HBL100 remained mostly unresponsive (Figure 4(A), right panel). At 5 µM, cell viability was down to 1% and 5% for compounds **5** and **14,** respectively.

As U-2 OS spheroids appeared the most responsive to our inhibitors, we tested our other hit compounds (**14**, **12**, **21**, **22**) at three concentrations (1, 2.5 and 5 µM) in cell viability assays after 2 and 10 days (Figure 4(B)). After 2-day treatments, spheroid viability was strongly affected by compounds **5**, **14** and **12** at 5 µM concentration (9.7, 6.9, 13.1% remaining viability, respectively) while compounds **21** and **22** showed no effects. Interestingly, after a 10-day treatment compound **12** appeared as the most effective inhibitor using U-2 OS cells, showing growth inhibition at 2.5 and 1 µM (50 and 66% remaining viability, respectively). For the other compounds these lower concentrations were mostly ineffective. However, a slight decrease in spheroid viability was observed for compound **21** at 5 µM concentration (69%). Taken together, our data showed that this series displayed some anti-proliferative properties without being generally cytotoxic. Results on U-2 OS spheroids clearly indicated stronger anti-proliferative effects for less selective compounds.

#### Effects on cellular Haspin kinase

**Figure 5.**
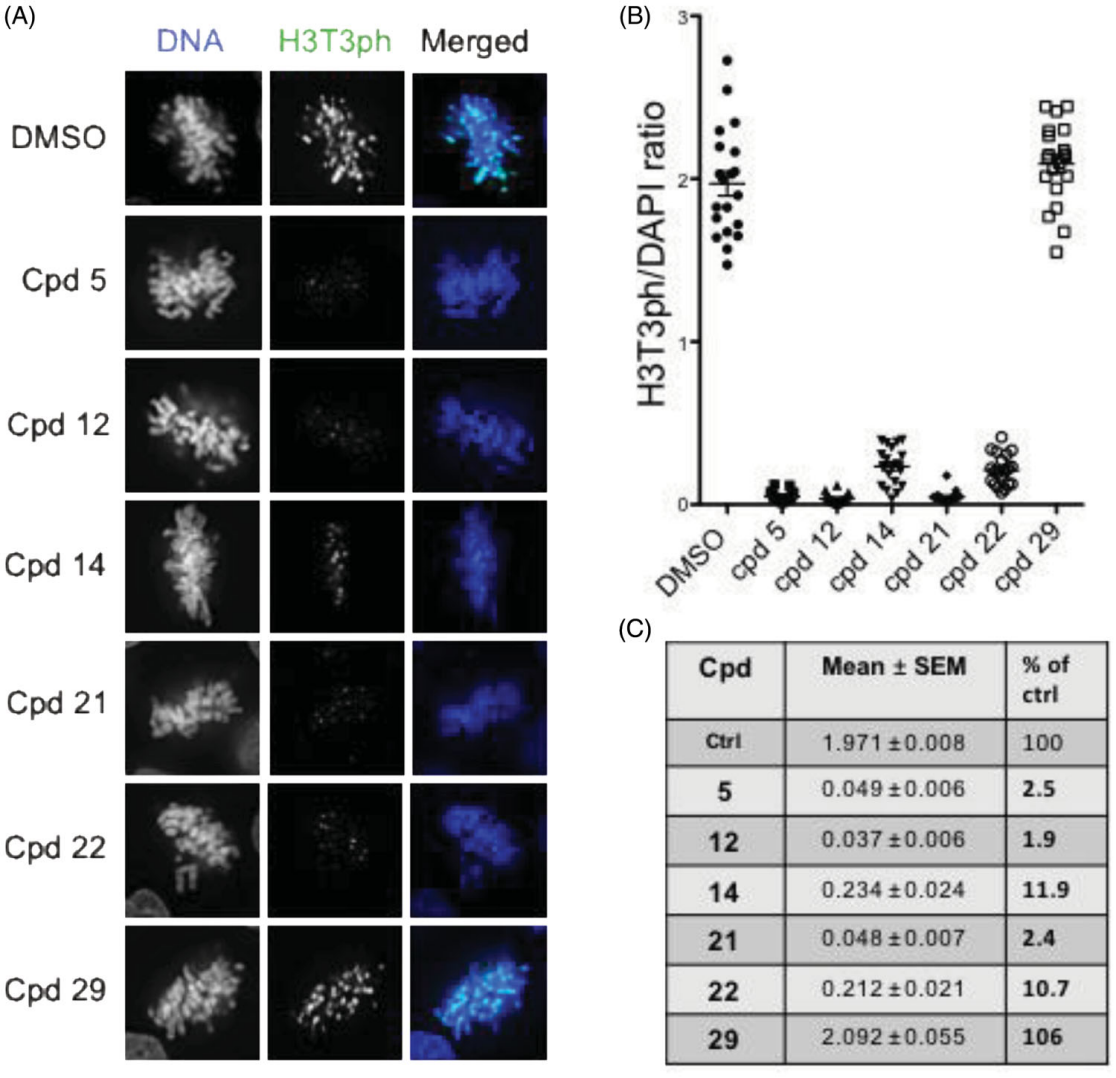
In-cell evaluation of Haspin inhibition. (A) immunofluorescence staining of U-2 OS cells treated for 24 h with 500 nM of each compound or 0.1% of DMSO. Haspin activity was monitored by staining of phosphorylated Histone H3 on threonine 3 (H3T3ph, green), deoxyribonucleic acid DNA was visualised by DAPI (4′,6-diamidino-2-phenylindole) staining (blue). Bar 10 µm. (B) Quantification of Haspin activity in prometaphase cells from (A); box and whiskers representation, *n* ≥ 30. (C) Statistical analysis of data obtained in (B). Results are mean ± SEM, *p* values are indicated (two-tailed unpaired *t*-test). Results are also shown in percentage of the DMSO control.

We next tested in cells the functional effects of compounds **12**, **14**, **21** and **22** as well as compounds **5** and **29** as reference and negative controls, respectively. We monitored and quantified Haspin activity by immunofluorescence and image analysis in U-2 OS cells following the phosphorylation of Threonine 3 of Histone H3 (H3T3ph) as a specific marker (Figure 5). Cells were treated with 0.5 µM of the various compounds for 24 h. As shown in Figure 5(A), treatment of cells with compounds **5, 12, 14**, **21** and **22** greatly reduced the H3T3ph signal in selected prometaphase/metaphase cells. We noted that typical chromosome misalignment defects were observed as previously reported in Haspin depletion studies^14^. Quantification of the H3T3ph signal *vs* DAPI is represented in Figure 5(B,C). Compounds **5**, **21** and **12** showed the strongest effect on cellular Haspin activity with only 2.5, 2.4 and 1.9% of H3T3ph signal remaining in prometaphase cells, respectively. This effect was slightly milder with compound **22-** and **14**-treated cells where 10.7 and 11.9% of H3T3p signal respectively remained (Figure 5(B,C)). The negative compound **29** showed a value similar to that of the DMSO control. A similar experiment performed on cells treated with a lower dose of 0.05 µM of compounds for 24 h revealed that cellular Haspin activity was still inhibited by more than 80% by compounds **5**, **12**, **21** and **22** (Supplementary Figure S5). These results showed that all tested compounds are highly cell penetrant and inhibit endogenous Haspin kinase. A comparison of their effects showed that compounds **5, 12** and **21** were the most efficient at reducing cellular Haspin activity.

#### Effects on cell cycle

**Figure 6.**
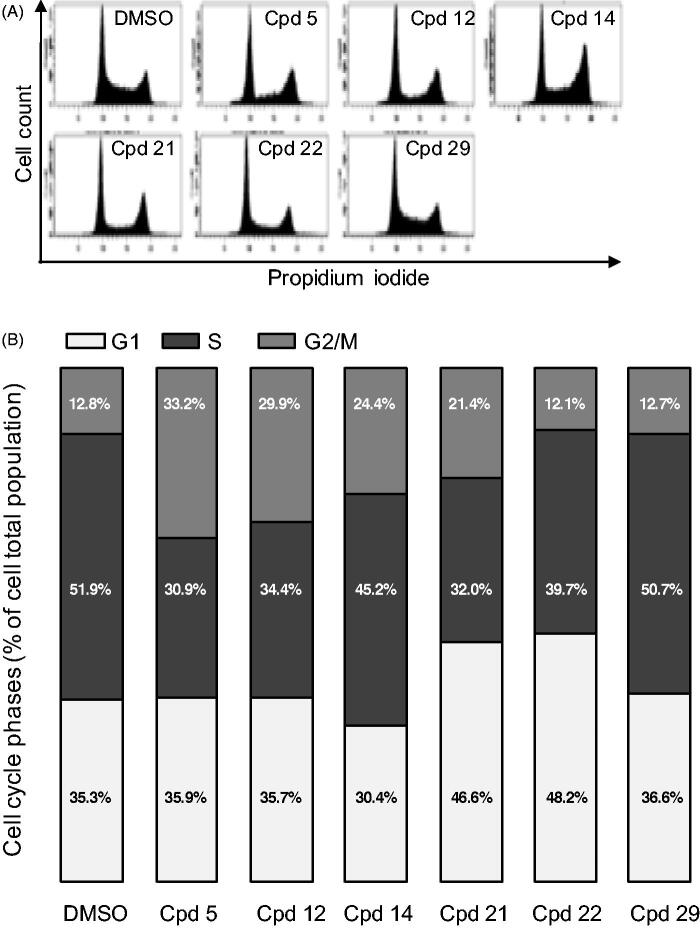
Cell cycle effects of selected compounds. (A) U-2 OS cells were treated with 2.5 µM of each compound for 24 h before ethanol fixation and propidium iodide staining. DNA content was measured by flow cytometry and representative profiles are shown. (B) quantification of the proportion of cells in each phase of the cell cycle from the experiment described in (A). Results are mean percentage (*n* = 3 independent experiments).

We next addressed the effect of our compounds on the cell cycle. U-2 OS cells were treated for 24 h with 2.5 µM of compounds and their cell cycle profile was analysed by flow cytometry (Figure 6). A lower dose of 0.5 µM was simultaneously tested showing no effect on the cell cycle of U-2 OS cells (data not shown), concurring with the results obtained on cell viability assays (Table 3). However, flow cytometry profiles, after treatment with compounds **5, 12, 14** and **21** at 2.5 µM, showed a strong increase of cells in G2/M phase of the cell cycle (33.3, 29.9, 24.4 and 21.4%, respectively vs. 12.8% in control DMSO-treated cells, Figure 6(A,B) accompanied with a reduction of cells in S phase (30.9, 34.4, 45.2 and 32%, respectively vs. 51.9% in control cells). These results are consistent with impaired cell cycle phase progression and transition checkpoints which are under the control of specific protein kinases, the Cyclin-dependent kinases (CDK/cyclin)^45^^,^^46^. The S-phase reduction coupled with a G2/M increase observed for compounds **5** and **12** could be in part explained by their strong inhibition of CDK2, a CDK essential for the progression through these phases (IC_50_ of 91 and 110 nM, respectively, Table 2)^46^. However, the S-phase reduction coupled with a G1 increase observed for compounds **21** and **22** cannot be explained by a CDK2 inhibition (IC_50_ of 4.3 and 5.3 µM, respectively). Such effects could result from the inhibition of G1-specific CDKs such as CDK4 and CDK6^47^^,^^48^, which remains to be tested.

While a slight delay in M phase is expected by inhibition of Haspin, the strong accumulation of cells in G2/M phases observed with compounds **5** and **12** cannot be solely linked to this inhibition but is likely the consequence of the inhibition of several kinases regulating mitotic entry. We recently reported inhibition of 5 on CDK1 (Cyclin-dependent kinase 1) during the first divisions of sea urchin embryos^49^. We therefore tested our compounds on two obvious candidates, *Hs*CDK1 and *Hs*PLK1 (Polo-like_kinase 1) both required for mitotic entry^50^ (Table 4).

**Table 4.**
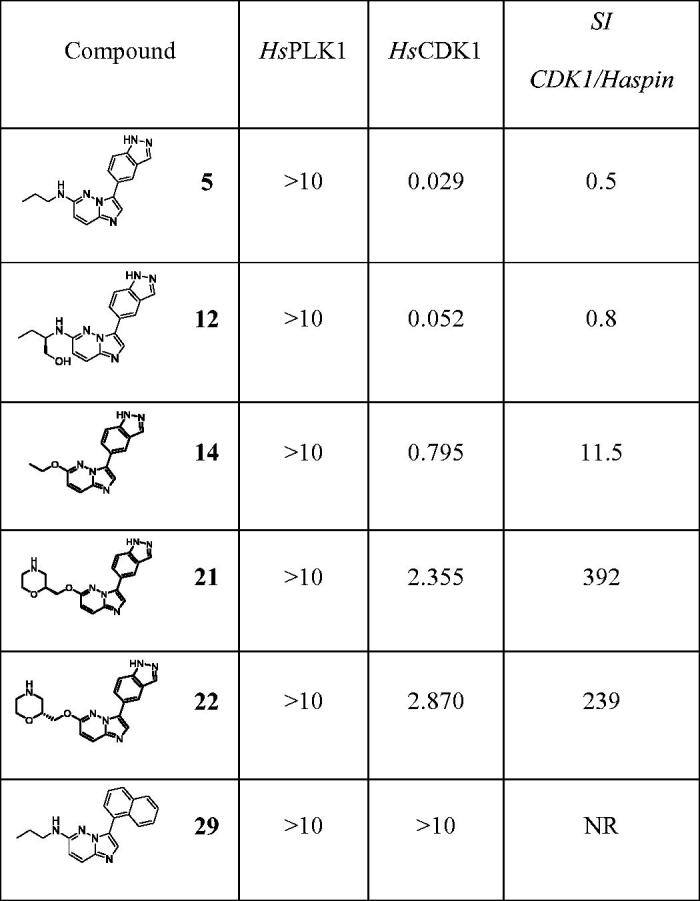
IC_50_ (µM) of selected compounds on *Hs*PLK1 and *Hs*CDK1.

Values are IC_50_ expressed in µM and calculated from dose–response curves (each point from the curves was performed in duplicate). SI are calculated as follows: SI = IC_50_ CDK1/IC_50_ Haspin (shown in Table 2). NR: not relevant.

None of the tested compounds inhibited PLK1 (IC_50_>10 µM). However, two compounds (**5** and **12)** showed strong inhibition of CDK1 (IC_50_ of 29 and 52 nM, respectively), which could account for the accumulation of cells in G2/M phase.

Altogether, these data showed that our compounds strongly affect the cell cycle in diverse ways. Some of these effects, shared by compounds **5** and **12,** can be explained by their ability to inhibit CDK1 and CDK2. The cell cycle kinase profile of these compounds could be of interest for further pharmacological development at a time when the marketing of CDK inhibitors such as Palbociclib, Abemaciclib or Ribociclib is thriving^51^.

#### Effects on cell migration

**Figure 7.**
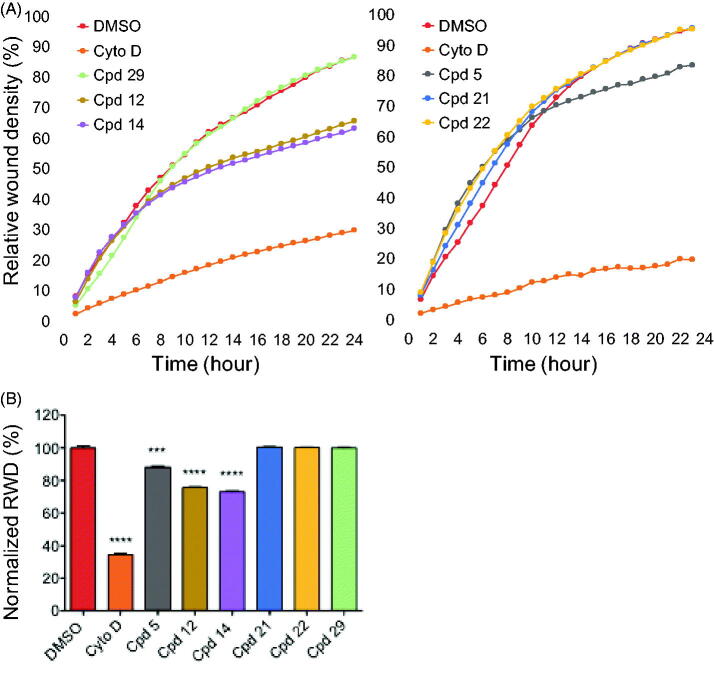
Effects of compounds on cell migration. (A) scratch wound cell migration assays were conducted on U-2 OS cells after treatment with 0.6 µM of each compound or 0.1 µg/mL cytochalasine D or 0.1% DMSO in two sets of experiments. Images were acquired every hour for 24 h 1by real-time live-cell microscopy (IncuCyte, Essen Bioscience) and images were analysed to determine the relative wound density (RWD) (IncuCyte software). Data are RWD mean ± SEM (*n* = 4). (B) Bar graph representation of the end point (24 h) of the experiment in A normalised against DMSO control (100%) for both sets of experiments. Results are mean percentage ± SEM; ***p ≤ 0.001, ****p ≤ 0.0001(two-tailed unpaired *t*-test).

We tested the effect of our most potent inhibitors on U-2 OS cell migration in a wound healing assay over a duration of 24 h where relative wound density (RWD) was measured every hour (Figure 7). The experiment was conducted in the presence of 0.5% FBS (fetal bovine serum). This condition allowed us to differentiate migrating cells from dividing cells which could interfere with the migration results. Compounds were tested at 0.6 µM alongside a DMSO control and cytochalasine D at 0.1 µg/mL as a positive control in two independent sets of experiments due to system limitations. The compound concentration was chosen as the maximum concentration that remained non-toxic to the cells for the length of the experiment in 0.5% FBS. After 24 h, cells treated with compounds **21** and **22** reached around 100% wound closure similar to the DMSO control and compound **29**-treated cells (Figure 7(A,B)). In contrast, both compound **12-** and **14**-treated cells migrated significantly slower, reaching 88 and 73% of normalised wound closure after 24 h, respectively (Figure 7(B)). The latter results confirmed that the observed effects were the result of an impaired cell migration not linked to cell division. Taken together, these results indicated that both compounds **12** and **14** exhibited an inhibitory effect on cell migration, suggesting an interesting anti-angiogenic potential. They also suggested that this effect on cell migration may not be linked to Haspin inhibition itself but rather to an off-target effect of these compounds.

## Conclusion

We synthesised and assessed a novel series of imidazopyridines starting from CHR-6494, a known Haspin inhibitor^24^. We tested the inhibitory properties of the compounds against *Hs*Haspin and showed that the highest activity was obtained with compounds **21** and **22** (6 and 12 nM, respectively) compared to that of CHR-6494 (55 nM). These two compounds also shared the highest SI towards all the protein kinases tested. We saw a modest increase in selectivity of compounds **12** and **14** which notably did not inhibit Aurora B kinase (average SI of 24.2 and 23.6, respectively). All the compounds tested achieved in-cell Haspin inhibition, with compounds **12** and **21** being the most effective. We demonstrated that our best hits, compounds **21** and **22,** were potent inhibitors of endogenous Haspin in human cells, which compared to the reference CHR-6494 or compound **12,** did not strongly affect G2/M transition due to their selectivity against CDK1 and CDK2. Hence, compounds **21** and **22** can be considered interesting and selective tools that will help to dissect Haspin function from other cell cycle regulating kinases. On the other hand, the lower SI of compound **12** and **14** endow them with substantial anti-proliferative properties against human cancer spheroids as well as anti-migrating effects. These highly sought-after properties mean that they could be used as starting material for the potential development of anti-cancer therapeutics.

## Supplementary Material

Supplemental Material
