# Supplementary material for: Design of new disubstituted imidazo[1,2-b]pyridazine derivatives as selective Haspin inhibitors. Synthesis, binding mode and anticancer biological evaluation
Source: J Enzyme Inhib Med Chem. 2020 Oct 12;35(1):1840–53. doi: 10.1080/14756366.2020.1825408 (PMC7580722; doi:10.1080/14756366.2020.1825408)
Supplement: Supplemental Material [file IENZ_A_1825408_SM3381.pdf]

Design of new disubstituted imidazo[1,2-*b*]pyridazine derivatives as selective Haspin inhibitors. Synthesis, binding mode and anticancer biological evaluation

Jonathan Elie[c,#], Omid Feizbakhsh[a,#], Nathalie Desban[a], Béatrice Josselin[a,b], Blandine Baratte[a,b], Amandine Bescond[a], Julien Duez[a,1], Xavier Fant[a], Stéphane Bach[a,b], Dominique Marie[f], Matthieu Place[c], Sami Ben Salah[c], Agnes Chartier[c], Sabine Berteina-Raboin[c], Apirat Chaikuad[d,e], Stefan Knapp[d,e], Fabrice Carles[c], Pascal Bonnet[c], Frédéric Buron[c,*], Sylvain Routier[c,*] and Sandrine Ruchaud[a,*]

[a] Sorbonne Université/CNRS UMR8227, Station Biologique, Place Georges Teissier, CS90074, 29688 Roscoff cedex, France

[b] Sorbonne Université/CNRS FR2424, Plateforme de criblage KISSf (Kinase Inhibitor Specialized Screening facility) Station Biologique, Place Georges Teissier, CS90074, 29688 Roscoff cedex, France

[c] Institut de Chimie Organique et Analytique, Université d'Orléans, UMR CNRS 7311, rue de Chartres, BP 6759, 45067 Orléans Cedex 2, France.

[d] Institute for Pharmaceutical Chemistry, Johann Wolfgang Goethe University, Max-von-Laue-Str. 9, D-60438 Frankfurt am Main, DE, Germany

[e] Structure Genomics Consortium, Johann Wolfgang Goethe University, Buchmann Institute for Molecular Life Sciences, Max-von-Laue-Str. 15, D-60438 Frankfurt am Main, DE, Germany

[f] Sorbonne Université/CNRS UMR7144, Station Biologique, Place Georges Teissier, CS90074, 29688 Roscoff cedex, France

[1] Present address: tebu-bio SAS, 39 rue de Houdan - BP 15, 78612 Le Perray-en-Yvelines Cedex, France.

The supporting information contains the materials, methods, and the ^{1}H NMR and ^{13}C NMR copy spectra.

1. Chemistry.

^{1}H NMR and ^{13}C NMR spectra were recorded on a Bruker DPX 250 MHz or 400 MHz instrument using $CDCl_3$ or DMSO-d_6. The chemical shifts are reported in parts per million (δ = scale) and all coupling constant (*J*) values are in Hertz (Hz). The following abbreviations were used to explain the multiplicities: s (singlet), d (doublet), t (triplet), q (quartet), m (multiplet) and dd (doublet doublet). Melting points are uncorrected. IR absorption spectra were obtained on a Perkin Elmer PARAGON 1000 PC and values are reported in cm^{-1}. HRMS were recorded on a Bruker maXis mass spectrometer by the "Fédération de Recherche" ICOA/CBM (FR2708) platform. Monitoring of the reactions was performed using silica gel TLC plates (silica Merck 60 F254). Spots were visualized by UV light at 254 nm and 356 nm. Column chromatographies were performed using silica gel 60 (0.063-0.200 mm, Merck).

General procedure A for Suzuki-Miyaura coupling: A solution of corresponding 3-bromoimidazopyridazines (1.0 equiv.), sodium carbonate (3.0 equiv.), and boronic acid (1.2 equiv.) in a mixture 1,4-dioxane/deionised water (0.15 M) was degassed by argon bubbling for 15 min. $Pd(PPh_3)_4$ (0.1 equiv.) was added and the mixture was heated at 150 °C for 1.5 h under microwave irradiation. The reaction mixture was then diluted in dichloromethane, dried over $MgSO_4$, filtered and purified by flash chromatography on silica gel.

General procedure B for S_NAr reaction: General procedure B1: Compound **7** (0.100 g, 0.37 mmol, 1 equiv.), NMP (2 mL), and the corresponding amine (5 equiv.) were introduced in an adapted microwave vial with a stir bar. The vial was then sealed and placed in the microwave oven. The mixture was heated at 180 °C during 1 h. After cooling, the mixture was poured into water and the aqueous layer was extracted three times with EtOAc. Organic layers were combined, dried over $MgSO_4$, filtrated, concentrated and purified with flash chromatography with EtOAc or EtOAc/MeOH as eluent. ***General procedure B2:*** NaH (5 equiv.), NMP (2 mL), and the corresponding amine or alcohol (5 equiv.) were introduced in an adapted microwave vial with a stir bar. The vial was then sealed and put in the microwave oven. The mixture was heated at 85 °C during 10 min. After cooling, the vial was opened and compound **6** (0.100 g, 0.37 mmol, 1 equiv.) was added. The vial was then resealed and placed in the microwave oven. The mixture was heated at 180 °C during 1 h. After cooling, the mixture was poured into NH_4Cl aqueous saturated solution and the aqueous layer was extracted three times with EtOAc. Organic layers were combined, dried over $MgSO_4$, filtrated, concentrated and purified by flash chromatography with EtOAc or EtOAc/MeOH as eluent. ***General procedure B3 :*** To a solution of corresponding alcohol (1.5 equiv.) in dry THF (0.1M) was added at 0 °C sodium hydride 60 % wt. in mineral oil (2.0 equiv.). The resulting solution was stirred at 0 °C for 5 min then compound **6** (1.0 equiv.) was introduced portion wise. The reaction was warmed at room temperature and stirred for 1-5 h. After this time, a saturated aqueous ammonium chloride solution (10 mL) was added, and the solution diluted with ethyl acetate (50 mL). The phase was separated, the aqueous phase extracted with ethyl acetate (2x20 mL), and combined organic phases were washed with water (50 mL), brine (50 mL) and dried over $MgSO_4$. After being concentrated under vacuum, the residue was purified by flash chromatography on silica gel.

6-(Propylamino)imidazo[1,2-*b*]pyridazine (3).

Compound **3** was synthesized under the general procedure B1 with compound **2** and *n*-propylamine. Purification was achieved by flash chromatography with EtOAc as eluent and the title compound **3** was obtained as a yellow solid in 86% yield. R_f (EtOAc): 0.11. m.p. 148-150 °C. ^{1}H NMR (400 MHz, $CDCl_3$) δ = 7.63 (s, 1H), 7.58 (d, *J* = 9.6 Hz, 1H), 7.46 (s, 1H), 6.38 (d, *J* = 9.6 Hz, 1H), 4.40 (s, 1H, NH), 3.30 (q, *J* = 7.0 Hz, 2H), 1.67 (h, *J* = 7.3 Hz, 2H), 1.00 (t, *J* = 7.4 Hz, 3H). ^{13}C NMR (101 MHz, $CDCl_3$) δ = 153.5 (C_q), 136.6 (C_q), 131.2 (CH), 126.0 (CH), 116.7 (CH), 111.8 (CH_r), 44.0 (CH_2), 22.5 (CH_2), 11.7 (CH_3). IR (ATR diamond, cm^{-1}) ν = 3225, 3104, 3040, 2958, 2933, 2886, 2854, 1625, 1576, 1485, 1472, 1459, 1334, 1306, 1269, 1210, 1152, 1137, 1121, 924, 808, 772, 758, 747, 638. HRMS (EI-MS): m/z calcd for $C_9H_{13}N_4$: 177.1135 $[M+H]^+$, found : 177.1138.

3-Bromo-6-(propylamino)imidazo[1,2-*b*]pyridazin-6-amine (4).

To a solution of compound **3** (0.500 g, 2.84 mmol, 1.0 equiv.) in ACN (20 mL) was added NBS (0.511 g, 2.84 mmol, 1.0 equiv.). The mixture was mixed at room temperature during 1 h. Then the solvent was removed and the residue was taken with DCM (10 mL), washed with water (40 mL). The aqueous layer was extracted three times with DCM. Organic layers were combined, dried over $MgSO_4$, filtrated, concentrated and the residue was purified by flash chromatography with EtOAc as eluent to give the compound **4** (0.702 g, 97 %) as a pale-yellow solid. R_f (AcOEt/EP 8/2) 0.11. m.p. 142-144 °C. ^{1}H NMR (400 MHz, $CDCl_3$) δ = 7.56 (d, *J* = 9.6 Hz, *J* = 0.6 Hz, 1H),

7.46 (s, 1H), 6.43 (d, J = 9.6 Hz, 1H), 4.58 (t, J = 5.6 Hz, 1H, NH), 3.29–3.48 (q, J = 7.3 Hz, 2H), 1.69 (m, 2H), 1.01 (t, J = 7.4 Hz, 3H). ^{13}C NMR (101 MHz, $CDCl_3$) δ = 154.0 (C_q), 137.2 (C_q), 131.4 (CH), 125.9 (CH), 111.6 (CH), 100.2 (C_q), 43.9 (CH_2), 22.5 (CH_2), 11.7 (CH_3). IR (ATR diamond, cm^{-1}) ν = 3254, 2958, 2876, 1620, 1572, 1475, 1459, 1325, 1135, 959, 831, 748. HRMS (EI-MS) m/z calcd for $C_9H_{12}BrN_4$: 255.0239 [M+H]$^+$, found : 255.0238.

3-(*1H*-Indazol-5-yl)-6-(propylamino)imidazo[1,2-*b*]pyridazine (5).

Compound **5** was synthesized under the general procedure A using compound **4** and 1*H*-indazole-5-boronic acid. After purification, the title compound was obtained (0.040 g, 28 %) as a yellow solid in 28% yield. R_f (EtOAc): 0.09. m.p. >250 °C. ^{1}H NMR (400 MHz, DMSO-d_6) δ = 13.13 (s, 1H), 8.73 (s, 1H), 8.10 (s, 1H), 8.04 (d, J = 8.8 Hz, 1H), 7.86 (s, 1H), 7.74 (d, J = 9.6 Hz, 1H), 7.61 (d, J = 8.8 Hz, 1H), 7.08 (t, J = 5.4 Hz, 1H), 6.69 (d, J = 9.6 Hz, 1H), 3.28 (q, J = 6.6 Hz, 2H), 1.70 (h, J = 7.3 Hz, 2H), 1.00 (t, J = 7.3 Hz, 3H). ^{13}C NMR (101 MHz, DMSO-d_6) δ = 153.4 (Cq), 138.8 (Cq), 136.8 (Cq), 133.9 (CH), 129.2 (CH), 127.2 (Cq), 125.5 (CH), 125.0 (CH), 123.0 (Cq), 121.8 (Cq), 117.3 (CH), 111.8 (CH), 110.2 (CH), 43.1 (CH_2), 21.4 (CH_2), 11.7 (CH_3). IR (ATR diamond, cm^{-1}) ν : 3297, 3104, 3043, 2951, 2911, 2868, 2729, 1625, 1579, 1492, 1469, 1430, 1372, 1331, 1283, 1173, 1147, 1077, 946, 894, 871, 829, 799, 785, 750. HRMS (EI-MS) m/z calcd for $C_{16}H_{17}N_6$: 293.1509 [M+H]$^+$, found : 293.1510.

6-Chloro-3-(*1H*-indazol-5-yl)imidazo[1,2-*b*]pyridazine (7).

Starting from **6** and 1*H*-indazole-5-boronic acid, compound **7** was obtained following the general procedure A and after flash chromatography (EtOAc as eluent) as a yellow solid in a 61% yield. R_f (EtOAc): 0.31. m.p. 224-226 °C. ^{1}H NMR (400 MHz, DMSO-d_6) δ = 13.24 (s, 1H), 8.55 (s, 1H), 8.30 (s, 1H), 8.30 (d, J = 9.5 Hz, 1H), 8.23 (s, 1H), 7.99 (dd, J = 8.7, 1.7 Hz, 1H), 7.70 (d, J = 8.7 Hz, 1H), 7.40 (d, J = 9.5 Hz, 1H). ^{13}C NMR (101 MHz, DMSO-d_6) δ = 146.2 (Cq), 139.3 (Cq), 138.2 (Cq), 134.2 (CH), 133.4 (CH), 128.5 (Cq), 128.0 (CH), 125.3 (CH), 122.9 (Cq), 119.9 (Cq), 118.6 (CH), 118.1 (CH), 110.6 (CH). IR (ATR diamond, cm^{-1}) ν : 3122, 3015, 2856, 1612, 1517, 1476, 1441, 1306, 1153, 1129, 1093, 947, 901, 840, 790, 775, 728. HRMS (EI-MS) m/z calcd for $C_{13}H_9ClN_5$: 270.0541 [M+H]$^+$, found : 270.0539.

3-(*1H*-Indazol-5-yl)-*N*-methyl-*N*-propylimidazo[1,2-*b*]pyridazin-6-amine (8).

Compound **8** was synthesized under the general procedure B1 with compound **7** and *N*-methyl-1-propylamine. Purification was achieved by flash chromatography with EtOAc as eluent and the title compound **8** was obtained as a yellow solid in a 28 % yield. R_f (EtOAc): 0.11. m.p. 205-207 °C. ^{1}H NMR (400 MHz, DMSO-d_6) δ = 13.14 (s, 1H), 8.73 (s, 1H), 8.11 (s, 1H), 8.06 (d, J = 8.8 Hz, 1H), 7.95 (s, 1H), 0.94 (t, J = 7.4 Hz, 3H), 7.87 (d, J = 9.6 Hz, 1H), 7.63 (d, J = 8.8 Hz, 1H), 7.06 (d, J = 9.9 Hz, 1H), 3.51 (t, J = 7.5 Hz, 2H), 1.66 (h, J = 7.5 Hz, 2H), 3.13 (s, 3H). ^{13}C NMR (101 MHz, DMSO-d_6) δ = 153.7 (Cq), 138.8 (Cq), 136.4 (Cq), 133.9 (CH), 130.0 (CH), 127.1 (Cq), 125.8 (CH), 125.0 (CH), 123.0 (Cq), 121.7 (Cq), 117.2 (CH), 110.2 (CH), 108.5 (CH), 52.1 (CH_2), 36.6 (CH_3), 19.8 (CH_2), 11.2 (CH_3). IR (ATR diamond, cm^{-1}) ν : 3107, 2863, 1628, 1566, 1482, 1429, 1330, 1164, 1096, 939, 891, 829, 789, 746. HRMS (EI-MS) m/z calcd for $C_{17}H_{19}N_6$: 307.1666 [M+H]$^+$, measured : 307.1665.

***N*-Cyclopentyl-3-(*1H*-indazol-5-yl)imidazo[1,2-*b*]pyridazin-6-amine (9).**

Compound **9** was synthesized under the general procedure B1 with compound **7** and cyclopentylamine. Purification was achieved by flash chromatography with EtOAc as eluent and the title compound was obtained as a beige solid in a 33% yield. R_f (EtOAc): 0.17. m.p. 195-197 °C. ^{1}H NMR (250 MHz, DMSO-d_6) δ = 13.12 (s, 1H), 8.76 (s, 1H), 8.09 (s, 1H), 8.05 (d, J = 8.2 Hz, 1H), 7.85 (s, 1H), 7.73 (d, J = 9.6 Hz, 1H), 7.61 (d, J = 8.8 Hz, 1H), 7.04 (d, J = 5.5 Hz, 1H), 6.67 (d, J = 9.7 Hz, 1H), 4.16–4.02 (m, 1H), 2.13–1.99 (m, 2H), 1.76–1.55 (m, 6H). ^{13}C NMR (63 MHz, DMSO-d_6) δ = 153.0 (Cq), 138.8 (Cq), 136.7 (Cq), 133.9 (CH), 129.1 (CH), 127.2 (Cq), 125.4 (CH), 125.0 (Cq), 123.0 (Cq), 121.9 (Cq), 117.3 (CH), 111.8 (CH), 110.1 (CH), 52.8 (CH), 32.2 (2xCH_2), 23.7 (2xCH_2). IR (ATR diamond, cm^{-1}) ν: 3247, 3097, 2942, 1628, 1573, 1485, 1331, 1270, 1165, 944, 887, 807, 784, 750. HRMS (EI-MS) m/z calcd for $C_{18}H_{19}N_6$: 319.1666 [M+H]$^+$, found : 319.1666.

***N*-Cyclohexyl-3-(*1H*-indazol-5-yl)imidazo[1,2-*b*]pyridazin-6-amine (10).**

Compound **10** was synthesized under the general procedure B1 with compound **7** and cyclohexylamine. Purification was achieved by flash chromatography with EtOAc as eluent and the title compound was obtained as a beige solid in a 16% Yield. R_f (EtOAc): 0.11. m.p. 240-242 °C. ^{1}H NMR (250 MHz, DMSO-d_6) δ = 13.13 (s,

1H), 8.79 (s, 1H), 8.06 (s, 1H), 8.02 (dd, *J* = 8.9, 1.6 Hz, 1H), 7.87 (s, 1H), 7.73 (d, *J* = 9.7 Hz, 1H), 7.60 (d, *J* = 8.9 Hz, 1H), 6.94 (d, *J* = 6.6 Hz, 1H), 6.68 (d, *J* = 9.7 Hz, 1H), 3.64 (dt, *J* = 9.6, 5.0 Hz, 1H), 2.16 (d, *J* = 11.7 Hz, 2H), 1.85–1.76 (m, 2H), 1.49–1.19 (m, 6H). ^{13}C NMR (63 MHz, DMSO-*d$_6$*) δ = 153.0 (Cq), 139.2 (Cq), 137.2 (Cq), 134.3 (CH), 129.6 (CH), 127.5 (Cq), 126.0 (CH), 125.5 (CH), 123.4 (Cq), 122.4 (Cq), 117.5 (CH), 112.3 (CH), 110.6 (CH), 50.8 (CH), 32.3 (2xCH_2), 26.1 (2xCH_2), 25.2 (CH_2). IR (ATR diamond, cm^{-1}) ν : 3278, 3097, 2923, 2844, 1736, 1628, 1573, 1484, 1329, 1278, 1169, 949, 892, 857, 800, 784, 751. HRMS (EI-MS) m/z calcd for $C_{19}H_{21}N_6$: 333.1822 [M+H]$^+$, found : 333.1823.

3-(*1H*-Indazol-5-yl)-6-(piperidin-1-yl)imidazo[1,2-*b*]pyridazine (11).

Compound **11** was synthesized under the general procedure B1 with compound **7** and piperidine. Purification was achieved by flash chromatography with EtOAc as eluent and the title compound was obtained as a white solid in a 16% yield. R$_f$ (EtOAc): 0.20. m.p. 231-233 °C. ^{1}H NMR (250 MHz, DMSO-*d$_6$*) δ = 13.14 (s, 1H), 8.63 (s, 1H), 8.14 (s, 1H), 8.03 (dd, *J* = 8.9, 1.6 Hz, 1H), 7.95 (s, 1H), 7.88 (d, *J* = 9.9 Hz, 1H), 7.63 (dt, *J* = 8.8, 0.9 Hz, 1H), 7.21 (d, *J* = 9.9 Hz, 1H), 3.57 (t, *J* = 3.8 Hz, 4H), 1.65 (s, 6H). ^{13}C NMR (63 MHz, DMSO-*d$_6$*) δ = 155.2 (Cq), 137.1 (Cq), 134.5 (CH), 130.7 (CH), 127.9 (Cq), 126.4 (Cq), 126.4 (CH), 125.5 (CH), 123.5 (Cq), 122.0 (Cq), 118.0 (CH), 110.8 (CH), 110.6 (CH), 47.4 (CH_2), 25.3 (2xCH_2), 24.5 (2xCH_2). IR (ATR diamond, cm^{-1}) ν = 3097, 2923, 2850, 1619, 1561, 1459, 1353, 1331, 1243, 1181, 1012, 945, 899, 852, 802, 772, 737. HRMS (EI-MS) m/z calcd for $C_{18}H_{19}N_6$: 319.1666 [M+H]$^+$, measured : 319.1669.

(*R*)-2-((3-(*1H*-Indazol-5-yl)imidazo[1,2-*b*]pyridazin-6-yl)amino)butan-1-ol (12).

Compound **12** was synthesized under the general procedure B1 with compound **7** and (R)-(-)-2-amino-1-butanol. Purification was achieved by flash chromatography with EtOAc then EtOAc/MeOH (9/1) as eluent to give the title compound as a pale yellow solid in a 13% yield. R$_f$ (EtOAc): 0.04. m.p. 219-221 °C. ^{1}H NMR (250 MHz, DMSO-*d$_6$*) δ = 13.13 (s, 1H), 8.71 (s, 1H), 8.09 (s, 1H), 8.04 (s, 1H), 7.84 (s, 1H), 7.73 (d, *J* = 9.6 Hz, 1H), 7.61 (d, *J* = 8.8 Hz, 1H), 6.82 (d, *J* = 7.7 Hz, 1H), 6.77 (d, *J* = 9.7 Hz, 1H), 4.74 (t, *J* = 5.3 Hz, 1H), 3.74 (p, *J* = 6.6 Hz, 1H), 3.58 (p, *J* = 5.3, 4.8 Hz, 2H), 1.67 (dq, *J* = 18.7, 6.9 Hz, 2H), 0.98 (t, *J* = 7.3 Hz, 3H). ^{13}C NMR (63 MHz, DMSO-*d$_6$*) δ = 153.3 (Cq), 138.8 (Cq), 136.8 (Cq), 133.9 (CH), 129.2 (CH), 127.1 (Cq), 125.4 (CH), 125.0 (CH), 123.0 (Cq), 121.8 (Cq), 117.3 (CH), 112.0 (CH), 110.2 (CH), 61.7 (CH_2), 54.6 (CH_3), 23.4 (CH_2), 10.7 (CH_3). IR (ATR diamond, cm^{-1}) ν = 3304, 3108, 2922, 1634, 1578, 1489, 1458, 1385, 1330, 1170, 1071, 981, 805, 750. HRMS (EI-MS) m/z calcd for $C_{17}H_{19}N_6O$: 323.1615 [M+H]$^+$, found : 323.1616. $[\alpha]_D^{20}$ (c = 1.0, DMSO): +0.501.

3-(*1H*-Indazol-5-*yl*)-6-propoxyimidazo[1,2-*b*]pyridazine (13).

Compound **13** was synthesized under the general procedure B2 with derivative **7** and propan-1-ol. Purification was achieved flash chromatography with EtOAc as eluent and the title compound was obtained as a white solid in a 32% yield. R$_f$ (EtOAc): 0.11. m.p. 198-200 °C. ^{1}H NMR (250 MHz, DMSO-*d$_6$*) δ = 13.18 (s, 1H), 8.64–8.60 (m, 1H), 8.16 (s, 1H), 8.10–8.00 (m, 3H), 7.66 (d, *J* = 8.8 Hz, 1H), 6.92 (d, *J* = 9.7 Hz, 1H), 4.36 (t, *J* = 6.6 Hz, 2H), 1.85 (h, *J* = 7.2 Hz, 2H), 1.03 (t, *J* = 7.4 Hz, 3H). ^{13}C NMR (63 MHz, DMSO-*d$_6$*) δ = 159.2 (Cq), 139.0 (Cq), 137.6 (Cq), 134.1 (CH), 131.1 (CH), 128.1 (Cq), 128.0 (CH), 125.1 (CH), 123.0 (Cq), 120.9 (Cq), 117.9 (CH), 110.9 (CH) 110.4 (CH), 68.7 (CH_2), 21.5 (CH_2), 10.3 (CH_3). IR (ATR diamond, cm^{-1}) ν = 3119, 2913, 1619, 1552, 1486, 1463, 1326, 1287, 1173, 1102, 952, 853, 799, 747. HRMS (EI-MS) m/z calcd for $C_{16}H_{16}N_5O$: 294.1349 [M+H]$^+$, found: 294.1353.

6-Ethoxy-3-(*1H*-indazol-5-yl)imidazo[1,2-*b*]pyridazine (14).

Compound **14** was synthesized under the general procedure B2 with compound **7** and ethanol. Purification was achieved by flash chromatography with EtOAc as eluent and the title compound was obtained as a pale green solid in a 27% yield. R$_f$ (EtOAc): 0.23. m.p. 205-207 °C. ^{1}H NMR (400 MHz, $CDCl_3$) δ = 10.36 (s, 1H), 8.54 (s, 1H), 8.16 (s, 1H), 8.00 (d, *J* = 8.6 Hz, 1H), 7.92 (s, 1H), 7.87 (d, *J* = 9.6 Hz, 1H), 7.61 (d, *J* = 8.7 Hz, 1H), 6.73 (d, *J* = 9.6 Hz, 1H), 4.46 (q, *J* = 7.1 Hz, 2H), 1.50 (t, *J* = 7.1 Hz, 3H); ^{13}C NMR (101 MHz, $CDCl_3$) δ = 159.6 (Cq), 139.5 (Cq), 138.3 (Cq), 135.7 (CH), 131.3 (CH), 129.0 (Cq), 127.8 (CH), 126.6 (CH), 123.7 (Cq), 122.4 (Cq), 119.1 (CH), 111.5 (CH), 110.0 (CH), 63.5 (CH_2), 14.5 (CH_3). IR (ATR diamond, cm^{-1}) ν = 3119, 2898, 1622, 1558, 1491, 1473, 1328, 1292, 1167, 1101, 1032, 942, 908, 806, 788. HRMS (EI-MS) m/z calcd for $C_{15}H_{14}N_5O$: 280.1193 [M+H]$^+$, found : 280.1192.

3-Bromo-6-[[(2*S*)-morpholin-2-yl]methanol]imidazo[1,2-*b*]pyridazine (15).

The reaction was carried out as described in general procedure B3 using **6** (121 mg, 0.52 mmol, 1.0 equiv.), (2*S*)-morpholin-2-ylmethanol hydrochloride (120 mg, 0.78 mmol, 1.5 equiv.), NaH 60 % in mineral oil (61 mg, 1.56 mmol, 3.0 equiv.) in dry THF (5 mL). The crude mixture was purified by flash chromatography on silica gel (DCM/MeOH/NH_4OH 95/4/1) to afford **15** as a white solid (131 mg, 80 %). R_f (AcOEt/EP 8/2) 0.07. m.p. 138-140 °C. ^{1}H NMR (250 MHz, $CDCl_3$) δ = 7.75 (d, *J* = 9.6 Hz, 1H), 7.58 (s, 1H), 6.77 (d, *J* = 9.6 Hz, 1H), 4.40 (d, *J* = 5.0 Hz, 2H), 4.03–3.84 (m, 2H), 3.68 (td, *J* = 11.0 Hz, *J* = 3.3 Hz, 1H), 3.09–2.71 (m, 4H), 1.83 (s, 1H, NH). ^{13}C NMR (63 MHz, $CDCl_3$) δ = 160.1 (C_q), 138.0 (C_q), 132.8 (CH), 127.7 (CH), 112.1 (CH), 100.9 (C_q), 74.7 (CH), 68.6 (CH_2), 68.2 (CH_2), 48.0 (CH_2), 45.9 (CH_2). IR (ATR diamond, cm^{-1}) ν = 3253, 2950, 2843, 1616, 1545, 1455, 1298, 1277, 1109, 1028, 850, 823. HRMS (EI-MS) m/z calcd for $C_{11}H_{14}BrN_4O_2$: 313.0295 $[M+H]^+$, found: 313.0294. $[\alpha]_D^{20}$ = +18.9 (c = 0.01 g.cm^{-3} / DMSO).

3-Bromo-6-[[(2*R*)-morpholin-2-yl]methanol]imidazo[1,2-*b*]pyridazine (16).

The reaction was carried out as described in general procedure B3 using **6** (121 mg, 0.52 mmol, 1.0 equiv.), (2*R*)-morpholin-2-ylmethanol hydrochloride (120 mg, 0.78 mmol, 1.5 equiv.), NaH 60 % in mineral oil (61 mg, 1.56 mmol, 3.0 equiv.) in dry THF (5 mL). The crude mixture was purified by flash chromatography on silica gel (DCM/MeOH/NH_4OH 95/4/1) to afford **16** as a white solid (130 mg, 80 %). R_f (DCM/MeOH 96/4) 0.11. m.p. 138-140 °C. ^{1}H NMR (250 MHz, $CDCl_3$) δ = 7.75 (d, *J* = 9.6 Hz, 1H), 7.58 (s, 1H), 6.77 (d, *J* = 9.6 Hz, 1H), 4.40 (d, *J* = 5.0 Hz, 2H), 4.03–3.84 (m, 2H), 3.68 (td, *J* = 11.0 Hz, *J* = 3.3 Hz, 1H), 3.09–2.71 (m, 4H), 1.83 (s, 1H, NH). ^{13}C NMR (63 MHz, $CDCl_3$) δ = 160.1 (C_q), 138.0 (C_q), 132.8 (CH), 127.7 (CH), 112.1 (CH), 100.9 (C_q), 74.7 (CH), 68.6 (CH_2), 68.2 (CH_2), 48.0 (CH_2), 45.9 (CH_2). IR (ATR diamond, cm^{-1}) ν = 3253, 2950, 2843, 1616, 1545, 1455, 1298, 1277, 1109, 1028, 850, 823. HRMS (EI-MS) m/z calcd for $C_{11}H_{14}BrN_4O_2$: 313.0295 $[M+H]^+$, found: 313.0294. $[\alpha]_D^{20}$ = -19.2 (c = 0.01 g.cm^{-3} / DMSO).

3-Bromo-6-[[(3S)-morpholin-3-yl]methanol]imidazo[1,2-*b*]pyridazine (17).

The reaction was carried out as described in general procedure B3 using **6** (98 mg, 0.43 mmol, 1.0 equiv.), (3*R*)-morpholin-3-ylmethanol hydrochloride (98 mg, 0.64 mmol, 1.5 equiv.), NaH 60 % in mineral oil (52 mg, 1.29 mmol, 3.0 equiv.) in dry THF (5 mL). The crude mixture was purified by flash chromatography on silica gel (DCM/MeOH/NH_4OH 95/4/1) to afford **17** as a white solid (97 mg, 73 %). R_f (DCM/MeOH 96/4) 0.10. m.p. 148-150 °C. ^{1}H NMR (250 MHz, $CDCl_3$) δ = 7.76 (d, *J* = 9.6 Hz, 1H), 7.58 (s, 1H), 6.71 (d, *J* = 9.6 Hz, 1H), 4.39–4.21 (m, 2H), 3.93 (dd, *J* = 10.6 Hz, *J* = 2.8 Hz, 1H), 3.82 (dd, *J* = 11.3 Hz, *J* = 3.1 Hz, 1H), 3.66–3.50 (m, 1H), 3.44 (t, *J* = 10.0 Hz, 1H), 3.36–3.21 (m, 1H), 3.05–2.90 (m, 2H), 2.11 (s, 1H, NH). ^{13}C NMR (63 MHz, $CDCl_3$) δ = 160.0 (C_q), 138.0 (C_q), 132.9 (CH), 127.8 (CH), 111.8 (CH), 101.0 (C_q), 69.4 (CH_2), 68.2 (CH_2), 67.7 (CH_2), 53.6 (CH), 45.4 (CH_2). IR (ATR diamond, cm^{-1}) ν = 3309, 3017, 2961, 2852, 1616, 1547, 1488, 1455, 1289, 1101, 863, 817. HRMS (EI-MS) m/z calcd for $C_{11}H_{15}BrN_4O_2$: 313.0295 $[M+H]^+$, found: 313.0293. $[\alpha]_D^{20}$ = +19.4 (c = 0.01 g.cm^{-3} / DMSO).

3-Bromo-6-[[(3*R*)-morpholin-3-yl]methanol]imidazo[1,2-*b*]pyridazine (18).

The reaction was carried out as described in general procedure B3 using **6** (150 mg, 0.64 mmol, 1.0 equiv.), (3*S*)-morpholin-3-ylmethanol hydrochloride (147 mg, 0.96 mmol, 1.5 equiv.), NaH 60 % in mineral oil (28 mg, 0.70 mmol, 1.1 equiv.) in dry THF (3 mL). The crude mixture was purified by flash chromatography on silica gel (DCM/MeOH/NH_4OH 95/4/1) to afford **18** as a white solid (144 mg, 72 %). R_f (DCM/MeOH 96/4) 0.10. m.p. 148-150 °C. ^{1}H NMR (250 MHz, $CDCl_3$) δ = 7.76 (d, *J* = 9.6 Hz, 1H), 7.58 (s, 1H), 6.71 (d, *J* = 9.6 Hz, 1H), 4.39–4.21 (m, 2H), 3.93 (dd, *J* = 10.6 Hz, *J* = 2.8 Hz, 1H), 3.82 (dd, *J* = 11.3 Hz, *J* = 3.1 Hz, 1H), 3.66–3.50 (m, 1H), 3.44 (t, *J* = 10.0 Hz, 1H), 3.36–3.21 (m, 1H), 3.05–2.90 (m, 2H), 2.11 (s, 1H, NH). ^{13}C NMR (63 MHz, $CDCl_3$) δ = 160.0 (C_q), 138.0 (C_q), 132.9 (CH), 127.8 (CH), 111.8 (CH), 101.0 (C_q), 69.4 (CH_2), 68.2 (CH_2), 67.7 (CH_2), 53.6 (CH), 45.4 (CH_2). IR (ATR diamond, cm^{-1}) ν = 3309, 3017, 2961, 2852, 1616, 1547, 1488, 1455, 1289, 1101, 863, 817. HRMS (EI-MS) m/z calcd for $C_{11}H_{14}BrN_4O_2$: 313.0295 $[M+H]^+$, found: 313.0293. $[\alpha]_D^{20}$ = -19.8 (c = 0.01 g.cm^{-3} / DMSO).

3-Bromo-6-[[(2*R*)-pyrrolidin-2-yl]methoxy]imidazo[1,2-*b*]pyridazine (19).

The reaction was carried out as described in general procedure B3 using **6** (250 mg, 1.1 mmol, 1.0 equiv.), D-Prolinol (120 µL, 1.2 mmol, 1.1 equiv.), NaH 60 % in mineral oil (50 mg, 1.26 mmol, 1.15 equiv.) in dry THF (5 mL). The crude mixture was purified by flash chromatography on silica gel (DCM/MeOH/NH_4OH 95/4/1) to afford **19** as a white solid (122 mg, 37 %). R_f (DCM/MeOH 97/3) 0.40. m.p. 109-110 °C. ^{1}H NMR (400 MHz,

MeOD) δ = 7.86 (d, *J* = 9.7 Hz, 1H), 7.60 (s, 1H), 6.93 (d, *J* = 9.7 Hz, 1H), 4.42 (dd, *J* = 10.8 Hz, *J* = 4.7 Hz, 1H), 4.31 (dd, *J* = 10.8 Hz, *J* = 7.5 Hz, 1H), 3.57 (qd, *J* = 7.4 Hz, *J* = 4.8 Hz, 1H), 3.05–2.85 (m, 2H), 2.08–1.97 (m, 1H), 1.93–1.75 (m, 2H), 1.66–1.54 (m, 1H). ^{13}C NMR (101 MHz, MeOD) δ = 162.0 (C_q), 139.3 (C_q), 132.9 (CH), 128.2 (CH), 114.4 (CH), 102.1 (C_q), 71.5 (CH_2), 58.0 (CH), 47.2 (CH_2), 29.1 (CH_2), 26.3 (CH_2). IR (ATR diamond, cm^{-1}) ν = 3021, 2962, 2838, 1546, 1489, 1455, 1291, 1201, 1002, 834, 749. HRMS (EI-MS) m/z calcd for $C_{11}H_{14}BrN_4O$: 297.0345 [M+H]$^+$, found: 297.0345. $[\alpha]_D^{20}$ = +11.4 (c = 0.01 g.cm^{-3} / DMSO).

3-Bromo-6-[[(2*S*)-pyrrolidin-2-yl]methoxy]imidazo[1,2-*b*]pyridazine (20).

The reaction was carried out as described in general procedure B3 using **6** (250 mg, 1.1 mmol, 1.0 equiv.), L-Prolinol (120 µL, 1.2 mmol, 1.1 equiv.), NaH 60 % in mineral oil (50 mg, 1.26 mmol, 1.15 equiv.) in dry THF (5 mL). The crude mixture was purified by flash chromatography on silica gel (DCM/MeOH/NH_4OH 95/4/1) to afford **20** as a white solid (67 mg, 24 %). R_f (DCM/MeOH 97/3) 0.40. m.p. 109-110 °C. ^{1}H NMR (400 MHz, MeOD) δ = 7.86 (d, *J* = 9.7 Hz, 1H), 7.60 (s, 1H), 6.93 (d, *J* = 9.7 Hz, 1H), 4.42 (dd, *J* = 10.8 Hz, *J* = 4.7 Hz, 1H), 4.31 (dd, *J* = 10.8 Hz, *J* = 7.5 Hz, 1H), 3.57 (qd, *J* = 7.4 Hz, *J* = 4.8 Hz, 1H), 3.05–2.85 (m, 2H), 2.08–1.97 (m, 1H), 1.93–1.75 (m, 2H), 1.66–1.54 (m, 1H). ^{13}C NMR (101 MHz, MeOD) δ = 162.0 (C_q), 139.3 (C_q), 132.9 (CH), 128.2 (CH), 114.4 (CH), 102.1 (C_q), 71.5 (CH_2), 58.0 (CH), 47.2 (CH_2), 29.1 (CH_2), 26.3 (CH_2). IR (ATR diamond, cm^{-1}) ν = 3021, 2962, 2838, 1546, 1489, 1455, 1291, 1201, 1002, 834, 749. HRMS (EI-MS) m/z calcd for $C_{11}H_{14}BrN_4O$: 297.0345 [M+H]$^+$, found: 297.0345. $[\alpha]_D^{20}$ = -11.9 (c = 0.01 g.cm^{-3} / DMSO).

3-(1*H*-Indazol-5-yl)-6-[[(2*S*)-morpholin-2-yl]methanol]imidazo[1,2-*b*]pyridazine (21).

The reaction was carried out as described in general procedure A using **15** (130 mg, 0.42 mmol, 1.0 equiv.), 1*H*-indazole-5-boronic acid (81 mg, 0.50 mmol, 1.2 equiv.) in a degassed mixture of 1,4-dioxane/deionised water (9/1, 5 mL). The crude mixture was purified by flash chromatography on silica gel (DCM/MeOH/NH_4OH 96/3/1) to afford **21** as a white solid (47 mg, 32 %). R_f (DCM/MeOH/NH_4OH 89/10/1) 0.10. m.p. 190-192 °C. ^{1}H NMR (400 MHz, MeOD) δ = 8.50 (s, 1H), 8.10 (s, 1H), 7.93 (dd, *J* = 8.8 Hz, *J* = 1.6 Hz, 1H), 7.89–7.84 (m, 3H), 7.61 (d, *J* = 8.8 Hz, 1H), 6.85 (d, *J* = 9.6 Hz, 1H), 4.34 (d, *J* = 4.9 Hz, 2H), 4.00–3.87 (m, 2H), 3.72–3.61 (m, 1H), 3.06–2.99 (m, 1H), 2.88–2.83 (m, 2H), 2.79–2.69 (m, 1H). ^{13}C NMR (101 MHz, MeOD) δ = 160.8 (C_q), 141.0 (C_q), 139.1 (C_q), 135.5 (CH), 131.1 (CH), 130.5 (C_q), 128.2 (CH), 127.3 (CH) 124.4 (C_q), 122.5 (C_q), 120.1 (CH), 112.9 (CH), 111.5 (CH), 75.1 (CH), 69.4 (CH_2), 68.1 (CH_2), 47.9 (CH_2), 45.9 (CH_2). IR (ATR diamond, cm^{-1}) ν = 3117, 2916, 2849, 1621, 1556, 1488, 1463, 1344, 1298, 1177, 1086, 1020, 945, 848, 805, 784. HRMS (EI-MS) m/z calcd for $C_{18}H_{19}N_6O_2$: 351.1564 [M+H]$^+$, found: 351.1562. $[\alpha]_D^{20}$ = +37.1 (c = 0.01 g.cm^{-3} / DMSO).

3-(1*H*-Indazol-5-yl)-6-[[(2*R*)-morpholin-2-yl]methanol]imidazo[1,2-*b*]pyridazine (22).

The reaction was carried out as described in general procedure A using **16** (130 mg, 0.42 mmol, 1.0 equiv.), 1*H*-indazole-5-boronic acid (81 mg, 0.50 mmol, 1.2 equiv.) in a degassed mixture of 1,4-dioxane/deionised water (9/1, 5 mL). The crude mixture was purified by flash chromatography on silica gel (DCM/MeOH/NH_4OH 96/3/1) to afford **22** as a white solid (48 mg, 33 %). R_f (DCM/MeOH/NH_4OH 89/10/1) 0.10. m.p. 190-192 °C. ^{1}H NMR (400 MHz, MeOD) δ = 8.56 (s, 1H), 8.14 (s, 1H), 7.99 (dd, *J* = 8.8 Hz, *J* = 1.6 Hz, 1H), 7.95–7.89 (m, 3H), 7.66 (d, *J* = 8.8 Hz, 1H), 6.92 (d, *J* = 9.7 Hz, 1H), 4.43 (d, *J* = 4.8 Hz, 2H), 4.07–3.94 (m, 2H), 3.80–3.63 (m, 1H), 3.15–3.09 (m, 1H), 2.96–2.91 (m, 2H), 2.90–2.80 (m, 1H). ^{13}C DEPT 135 NMR (101 MHz, MeOD) δ = 135.9 (CH), 131.6 (CH), 128.8 (CH), 127.8 (CH), 120.6 (CH), 113.4 (CH), 112.0 (CH), 75.1 (CH), 69.6 (CH_2), 68.0 (CH_2), 47.9 (CH_2), 46.0 (CH_2). IR (ATR diamond, cm^{-1}) ν = 3189, 2917, 2849, 1621, 1557, 1489, 1463, 1344, 1288, 1178, 1085, 1020, 945, 849, 807. HRMS (EI-MS) m/z calcd for $C_{18}H_{19}N_6O_2$: 351.1564 [M+H]$^+$, found : 351.1562 . $[\alpha]_D^{20}$ = -36.5 (c = 0.01 g.cm^{-3} / DMSO).

3-(1*H*-indazol-5-yl)-6-[[(3*S*)-morpholin-3-yl]methanol]imidazo[1,2-*b*]pyridazine (23).

The reaction was carried out as described in general procedure A using **17** (90 mg, 0.29 mmol, 1.0 equiv.), 1*H*-indazole-5-boronic acid (57 mg, 0.35 mmol, 1.2 equiv.) in a degassed mixture of 1,4-dioxane/deionised water (9/1, 4 mL). The crude mixture was purified by flash chromatography on silica gel (DCM/MeOH/NH_4OH 96/3/1) to afford **23** as a white solid (53 mg, 52 %). R_f (DCM/MeOH 96/4) 0.09. m.p. 212-214 °C. ^{1}H NMR (400 MHz, MeOD) δ = 8.53 (s, 1H), 8.13 (s, 1H), 8.04–7.84 (m, 5H), 7.64 (d, *J* = 8.8 Hz, 1H), 6.92 (d, *J* = 9.8 Hz, 1H), 4.39-4.23 (m, 2H), 3.96 (dd, *J* = 11.4 Hz, *J* = 3.1 Hz, 1H), 3.81 (d, *J* = 11.3 Hz, 1H), 3.61–3.52 (m, 1H), 3.45 (t, *J* = 10.3 Hz, 1H), 3.35–3.31 (m, 1H), 2.97–2.90 (m, 2H). ^{13}C NMR (101 MHz, MeOD) δ = 159.5 (C_q), 139.7 (C_q), 137.8 (C_q), 134.1 (CH), 129.8 (CH), 129.3 (C_q), 126.9 (CH), 126.0 (CH), 123.0 (C_q), 121.1 (C_q), 118.8 (CH), 111.6

(CH), 110.1 (CH), 68.5 (CH_2), 67.2 (CH_2), 66.6 (CH_2), 53.1 (CH), 44.6 (CH_2). IR (ATR diamond, cm^{-1}) ν = 3118, 2958, 2855, 2359, 1622, 1556, 1489, 1463, 1322, 1289, 1174, 1101, 1016, 945, 830, 804, 786. HRMS (EI-MS) m/z calcd for $C_{18}H_{19}N_6O_2$: 351.1564 $[M+H]^+$, found : 351.1563. $[\alpha]_D^{20}$ = +29.4 (c = 0.01 $g.cm^{-3}$ / DMSO).

3-(1*H*-Indazol-5-yl)-6-[[(3*R*)-morpholin-3-yl]methanol]imidazo[1,2-*b*]pyridazine (24).

The reaction was carried out as described in general procedure A using **18** (90 mg, 0.29 mmol, 1.0 equiv.), 1*H*-indazole-5-boronic acid (57 mg, 0.35 mmol, 1.2 equiv.) in a degassed mixture of 1,4-dioxane/deionised water (9/1, 4 mL). The crude mixture was purified by flash chromatography on silica gel (DCM/MeOH/NH_4OH 96/3/1) to afford **24** as a white solid (55 mg, 54 %). R_f (DCM/MeOH 96/4) 0.09. m.p. 212-214 °C. ^1H NMR (250 MHz, $CDCl_3$) δ = 10.52 (s, 1H, NH), 8.45 (s, 1H), 8.16 (s, 1H), 8.01–7.84 (m, 3H), 7.61 (d, *J* = 8.8 Hz, 1H), 6.75 (d, *J* = 9.6 Hz, 1H), 4.47–4.19 (m, 2H), 3.94 (dd, *J* = 10.4 Hz, *J* = 2.4 Hz, 1H), 3.85 (dt, *J* = 11.2 Hz, *J* = 2.8 Hz, 1H), 3.68–3.54 (m, 1H), 3.53-3.26 (m, 3H), 3.07-2.91 (m, 2H). ^{13}C NMR (63 MHz, $CDCl_3$) δ = 159.3 (C_q), 139.6 (C_q), 138.3 (C_q), 135.6 (CH), 131.6 (CH), 129.2 (C_q), 128.1 (CH), 126.5 (CH), 123.7 (C_q), 122.1 (C_q), 119.3 (CH), 110.9 (CH), 110.1 (CH), 69.4 (CH_2), 68.1 (CH_2), 67.8 (CH_2), 53.6 (CH), 45.5 (CH_2). IR (ATR diamond, cm^{-1}) ν = 3115, 2853, 1622, 1551, 1458, 1318, 1289, 1251, 1098, 948, 885, 790. HRMS (EI-MS) m/z calcd for $C_{18}H_{18}N_6O_2$: 351.1564 $[M+H]^+$, found : 351.1566. $[\alpha]_D^{20}$ = -29.8 (c = 0.01 $g.cm^{-3}$ / DMSO).

3-(1*H*-Indazol-5-yl)-6-[[(2*R*)-pyrrolidin-2-yl]methoxy]imidazo[1,2-*b*]pyridazine (25).

The reaction was carried out as described in general procedure A using **19** (227 mg, 0.76 mmol, 1.0 equiv.), *1H*-indazole-5-boronic acid (148 mg, 0.91 mmol, 1.2 equiv.) in a degassed mixture of 1,4-dioxane/deionised water (9/1, 10 mL). The crude mixture was purified by flash chromatography on silica gel (DCM/MeOH/NH_4OH 96/3/1) to afford **25** as a white solid (63 mg, 25 %). R_f (DCM/MeOH 97/3) 0.21. m.p. 169-171 °C. ^1H NMR (250 MHz, MeOD) δ = 8.51 (s, 1H), 8.10 (s, 1H), 7.94 (dd, *J* = 8.8 Hz, *J* = 1.6 Hz, 1H), 7.89–7.83 (m, 3H), 7.61 (dd, *J* = 8.8 Hz, *J* = 0.9 Hz, 1H), 6.86 (d, *J* = 9.6 Hz, 1H), 4.44–4.20 (m, 2H), 3.70–3.51 (m, 1H), 3.06–2.87 (m, 2H), 2.13–1.94 (m, 1H), 1.91–1.77 (m, 2H), 1.71-1.51 (m, 1H). ^{13}C NMR (63 MHz, MeOD) δ = 161.0 (C_q), 141.0 (C_q), 139.2 (C_q), 135.5 (CH), 131.1 (CH), 130.5 (C_q), 128.1 (CH), 127.3 (CH), 124.4 (C_q), 122.6 (C_q), 120.0 (CH), 113.1(CH), 111.5 (CH), 71.1 (CH_2), 58.1 (CH), 47.2 (CH_2), 29.1 (CH_2), 26.2 (CH_2). IR (ATR diamond, cm^{-1}) ν = 3108, 2922, 1621, 1557, 1489, 1404, 1287, 1164, 945, 803. HRMS (EI-MS) m/z calcd for $C_{18}H_{19}N_6O$: 335.1615 $[M+H]^+$, found : 335.1614. $[\boldsymbol{\alpha}]_D^{20}$ = +28.3 (c = 0.01 $g.cm^{-3}$ / DMSO).

3-(1*H*-Indazol-5-yl)-6-[[(2*S*)-pyrrolidin-2-yl]methoxy]imidazo[1,2-*b*]pyridazine (26).

The reaction was carried out as described in general procedure A using **20** (227 mg, 0.76 mmol, 1.0 equiv.), *1H*-indazole-5-boronic acid (148 mg, 0.91 mmol, 1.2 equiv.) in a degassed mixture of 1,4-dioxane/deionised water (9/1, 10 mL). The crude mixture was purified by flash chromatography on silica gel (DCM/MeOH/NH_4OH 96/3/1) to afford **26** as a white solid (63 mg, 25 %). R_f (DCM/MeOH 97/3) 0.21. m.p. 169-171 °C. ^1H NMR (250 MHz, MeOD) δ = 8.52 (s, 1H), 8.10 (s, 1H), 7.94 (dd, *J* = 8.8 Hz, *J* = 1.6 Hz, 1H), 7.91–7.85 (m, 3H), 7.62 (dd, *J* = 8.8 Hz, *J* = 0.9 Hz, 1H), 6.86 (d, *J* = 9.6 Hz, 1H), 4.46–4.21 (m, 2H), 3.72–3.55 (m, 1H), 3.10–2.90 (m, 2H), 2.15–1.94 (m, 1H), 1.92–1.79 (m, 2H), 1.72–1.52 (m, 1H). ^{13}C NMR (63 MHz, MeOD) δ = 161.0 (C_q), 141.0 (C_q), 139.2 (C_q), 135.5 (CH), 131.2 (CH), 130.5 (C_q), 128.2 (CH), 127.3 (CH), 124.5 (C_q), 122.6 (C_q), 120.0 (CH), 113.1 (CH), 111.5 (CH), 71.1 (CH_2), 58.1 (CH), 47.2 (CH_2), 29.1 (CH_2), 26.2 (CH_2). IR (ATR diamond, cm^{-1}) ν = 3192, 2918, 2871, 1618, 1563, 1463, 1321, 1171, 1041, 947, 796. HRMS (EI-MS) m/z calcd for $C_{18}H_{18}N_6O$: 335.1615 $[M+H]^+$, found: 335.1615. $[\alpha]_D^{20}$ = -37.1 (c = 0.01 $g.cm^{-3}$ / DMSO).

3-(*1H*-Benzo[*d*]imidazol-5-yl)-*N*-propylimidazo[1,2-*b*]pyridazin-6-amine (27).

The reaction was carried out as described in general procedure A using **4** (0.100 g, 0.39 mmol, 1 equiv.), 1*H*-benzimidazole-5-boronic acid pinacol ester (0.147 g, 0.58 mmol, 1.5 equiv.) and Cs_2CO_3 (0.384 g, 1.17 mmol, 3 equiv.). The crude mixture was purified by flash chromatography on silica gel (EtOAc) to afford **27** (0.026 g, 23 %) as a yellow solid. R_f (EtOAc,/MeOH, 9/1): 0.14. m.p. 242-244 °C. ^1H NMR (400 MHz, DMSO-d_6) δ = 12.54 (d, *J* = 37.3 Hz, 1H), 8.57 (d, *J* = 35.9 Hz, 1H), 8.24 (s, 1H), 7.89 (d, *J* = 31.4 Hz, 2H), 7.73 (d, *J* = 9.6 Hz, 1H), 7.71–7.56 (m, 1H), 7.04 (t, *J* = 5.3 Hz, 1H), 6.69 (d, *J* = 9.6 Hz, 1H), 3.29 (d, *J* = 7.6 Hz, 2H), 1.68 (h, *J* = 7.3 Hz, 2H), 1.00 (t, *J* = 7.2 Hz, 3H). ^{13}C NMR (101 MHz, DMSO-d_6) δ = 153.4 (CH), 144.3 (Cq), 142.5 (CH), 136.8 (Cq), 136.1 (Cq), 129.3 (CH), 127.5 (Cq), 125.5 (CH), 123.4 (Cq), 120.7 (CH), 111.6 (CH), 105.0 (CH), 99.5

(Cq), 43.0 (CH_2), 21.5 (CH_2), 11.7 (CH_3). IR (ATR diamond, cm^{-1}) ν: 3059, 2951, 1628, 1590, 1476, 1330, 1286, 1260, 1164, 827, 798, 754. HRMS (EI-MS) m/z calcd for $C_{16}H_{17}N_6$: 293.1509 $[M+H]^+$, found : 293.1506.

3-(2-Aminopyrimidin-5-yl)-6-(propylamino)imidazo[1,2-*b*]pyridazine (28).

The reaction was carried out as described in general procedure A using **4** (0.150 g, 0.58 mmol, 1 equiv.), 2-aminopyrimidine-5-boronic acid (0.122 g, 0.88 mmol, 1.5 equiv.) and Cs_2CO_3 (0.575 g, 1.75 mmol, 3 equiv.). The crude mixture was purified by flash chromatography on silica gel (EtOAc) to afford **28** (0.115 g, 73 %) as a yellow solid. R_f (EtOAc): 0.07. m.p. 87-89 °C. ^1H NMR (400 MHz, DMSO-d_6) δ = 8.97 (s, 2H), 7.77 (s, 1H), 7.71 (d, *J* = 9.6 Hz, 1H), 7.06 (t, *J* = 5.3 Hz, 1H), 6.81 (s, 2H), 6.66 (d, *J* = 9.7 Hz, 1H), 3.20 (q, *J* = 6.6 Hz, 2H), 1.63 (h, *J* = 7.3 Hz, 2H), 0.96 (t, *J* = 7.4 Hz, 3H). ^{13}C NMR (101 MHz, DMSO-d_6) δ = 162.1 (Cq), 153.5 (Cq), 155.2 (2xCH), 136.5 (Cq), 127.6 (CH), 125.4 (CH), 122.9 (Cq), 112.9 (Cq), 111.8 (CH), 42.9 (CH_2), 21.3 (CH_2), 11.7 (CH_3). IR (ATR diamond, cm^{-1}) ν : 3492, 3254, 3154, 3050, 2951, 1628, 1605, 1582, 1556, 1469, 1368, 1328, 1289, 1170, 956, 809, 792, 754, 656. HRMS (EI-MS) m/z calcd for $C_{13}H_{16}N_7$: 270.1462 $[M+H]^+$, found : 270.1463.

3-(Naphthalen-1-yl)-*N*-propylimidazo[1,2-*b*]pyridazin-6-amine (29).

The reaction was carried out as described in general procedure A using **4** (0.100 g, 0.39 mmol, 1 equiv.), 1-naphthaleneboronic acid (0.083g, 0.47 mmol, 1.2 equiv.) and Cs_2CO_3 (0.381 g, 1.17 mmol, 3 equiv.). The crude mixture was purified by flash chromatography on silica gel (EtOAc) to afford (0. 083 g, 70 %) as a white solid. R_f (EtOAc): 0.17. m.p. 144-146 °C. ^1H NMR (400 MHz, DMSO-d_6) δ = 7.99 (d, *J* = 8.1 Hz, 2H), 7.81–7.72 (m, 3H), 7.64–7.58 (m, 2H), 7.57–7.52 (m, 1H), 7.47 (ddd, *J* = 8.2, 6.8, 1.4 Hz, 1H), 6.89 (t, *J* = 5.4 Hz, 1H), 6.71 (d, *J* = 9.7 Hz, 1H), 2.85 (q, *J* = 6.7 Hz, 2H), 1.39 (h, *J* = 7.3 Hz, 2H), 0.71 (t, *J* = 7.4 Hz, 3H). ^{13}C NMR (101 MHz, DMSO-d_6) δ = 153.4 (Cq), 136.4 (Cq), 133.3 (Cq), 131.3 (CH), 131.2 (Cq), 128.5 (CH), 128.2 (CH), 128.1 (CH), 126.7 (Cq), 126.2 (Cq), 126.1 (CH), 126.0 (CH), 125.9 (CH), 125.4 (CH), 125.3 (CH), 112.6 (CH), 42.5 (CH_2), 21.3 (CH_2), 11.4 (CH_3). IR (ATR diamond, cm^{-1}) ν : 3243, 3047, 3958, 2933, 2869, 1627, 1583, 1487, 1324, 1274, 1159, 935, 804, 792, 765, 751. HRMS (EI-MS) m/z calcd for $C_{19}H_{19}N_4$: 303.1604 $[M+H]^+$, found : 303.1607.

3-(*1H*-Indazol-5-yl)imidazo[1,2-*b*]pyridazine (30).

In a 2-5 mL microwave vial with a stir bar was introduced compound **7** (0.100 g, 0.37 mmol, 1 equiv.), THF (2 mL), Et_3N (0.16 mL, 1.12 mmol, 3 equiv.) then formic acid (0.03 mL, 0.74 mmol, 2 equiv.). The mixture was degassed with inert gas during 15 min and $Pd(OAc)_2$ (0.008g, 0.01 equiv.) and Xantphos (0.043 g, 0.02 equiv.) were added. The vial was then sealed and placed in the microwave oven. The mixture was heated at 150 °C during 15 min. After cooling, the solvent was removed and the residue was purified by flash chromatography with EtOAc as eluent to give compound **17** (0.032 g, 37 %) as a yellow solid. R_f (EtOAc): 0.33. m.p. 98-100 °C. ^1H NMR (400 MHz, DMSO-d_6) δ = 13.20 (s, 1H), 8.66 (q, *J* = 2.0 Hz, 2H), 8.29–8.17 (m, 3H), 8.03 (dd, *J* = 8.9, 1.6 Hz, 1H), 7.68 (d, *J* = 8.8 Hz, 1H), 7.29 (dd, *J* = 9.2 Hz, *J* = 4.4 Hz, 1H). ^{13}C NMR (101 MHz, DMSO-d_6) δ = 143.8 (CH), 139.5 (Cq), 139.1 (Cq), 134.1 (CH), 132.4 (CH), 126.0 (CH), 125.4 (CH), 122.9 (Cq), 120.7 (Cq), 119.5 (Cq), 118.3 (CH), 116.7 (CH), 110.5 (CH). IR (ATR diamond, cm^{-1}) ν: 3151, 1615, 1524, 1489, 1372, 1334, 1292, 1169, 1109, 947, 846, 798, 776. HRMS (EI-MS) m/z calcd for $C_{13}H_{10}N_5$: 236.0931 $[M+H]^+$, found : 236.0929.

2. Kinase assays

Recombinant protein kinases were expressed and purified from *E. coli* (*Hs*Haspin kinase domain aa470-798, *Hs*Cdk5/p25, *Rn*DYRK1A kinase domain aa1-499, *Hs*PIM1, *Mm*CLK1) or baculovirus/Sf9 cells (*Hs*Cdk2/Cyclin A, *Hs*Cdk9/Cyclin T). Native *Ssc*CK1δ /ε and *Ssc*GSK3α/β were purified from pig brain whereas *Hs*Plk1, *Hs*Cdk1 and *Hs*Aurora B were purchased from ProQinase and SignalChem respectively. Kinase assays were performed in specific buffers and substrates for each kinase tested in the presence of 15 µM [γ-^{33}P] ATP (3,000 Ci/mmol, 10 mCi/ml; PerkinElmer) in a final volume of 30 µl as previously reported [51]. After 30 min incubation at 30°C, the reaction mix was spotted onto GE healthcare P81 phosphocellulose paper using a FilterMate Harvester (PerkinElmer), and the filter was extensively washed with a 1% phosphoric acid solution. The wet filter-bound radioactivity was determined in the presence of 20 µl scintillation fluid (GE healthcare) with a TopCount® Scintillation and Luminescence Counter (PerkinElmer). IC_{50} values were calculated from dose-response curves out of duplicate points.

Kinase activities using the ADP-Glo methodology (ADP-Glo Kinase Assay; Promega) were assayed according to the assay described in Nguyen *et al.* [52] and Ibrahim *et al.* [53].

3. Crystollagraphic experimental part

Recombinant Haspin was prepared as previously described [54]. Crystallization was performed using the sitting-drop vapor diffusion method at 4 °C and the condition containing 63% MPD and 0.1 M SPG, pH 6.5. Diffraction data collected at the Diamond beamline i03 were processed and scaled with iMOSFLM [55] and Scala [56], respectively. Molecular replacement was performed using Phaser [57] and the published coordinates of Haspin [58]. Manual model rebuilding alternated with structure refinement was performed in COOT [59] and REFMAC [60], respectively. Data collection and refinement statistics are summarized in Table 6.

Table 6. Data collection and refinement statistics

	**Haspin-12**
**Data collection**	
Space group	$P\ 2_1 2_1 2_1$
Cell dimensions	
a, *b*, *c* (Å)	70.4, 78.0, 86.2
α, *β*, *γ* (°)	90.0, 90.0, 90.0
Resolution (Å)	70.35-1.65 (1.74-1.65)*
R_{merge}	0.067 (0.776)
$I/\sigma I$	11.9 (2.2)
Completeness (%)	98.3 (97.1)
Redundancy	5.3 (5.1)
**Refinement**	
No. reflections	56,638 (8,080)
R_{work} / R_{free}	0.159/ 0.179
No. atoms	
Protein	2,688
Compound	24
Water and solvents	315
B factors (Å^2)	
Protein	31
Compounds (SM1-71)	20
Water and solvents	42
r.m.s. deviations	
Bond lengths (Å)	0.016
Bond angles (°)	1.6

* numbers in brackets are for the high-resolution shell.

4. Molecular modeling methods

Molecular modelling studies were performed with MOE2016 (Molecular Operating Environment) [61]. The crystal structure of CHR-6494 bound to Haspin was used for docking. First, the structure was prepared with the standard protocol QuickPrep implemented in the MOE software and tethering receptor, ligand and solvent molecules during preparation. The ligand, water molecules and ions were removed from the structure. The inhibitor **21** was prepared

from SMILES notation with VSprep [62]. Docking was performed with the Dock tool implemented in the MOE software using default parameters. The MMGBSA scoring function was used to rank the best docking poses.

5. Cellular evaluation methods

5.1 Cell culture

HCT116 cells were cultured in McCoy's medium. SH-SY5Y, HBL100 and U-2 OS cells were cultured in Dulbecco's modified Eagle's medium (DMEM) and hTERT RPE-1 cells in DMEM:F12 medium. All media were supplemented with 10% fetal calf serum (unless overwise specified) and 2 mM L-glutamine and cells were cultured at 37°C in a 5% CO_2 humidified atmosphere.

5.2 Cell viability on monolayer cultures (2D)

Briefly, cells were grown in 96-well plates in the presence of increasing concentrations of each compound (from 50 to 0.05 µM) for 48 h. Cell viability was then assessed using the CellTiter96 AQueous cell proliferation assay from Promega according to manufacturer's instructions. Each experiment was done in triplicate and IC_{50} were determined from the dose-response curves according to the signal given by the control (0.1% DMSO) set at 100% viability.

5.3 Cell viability on spheroid cultures (3D)

For the experiment in figure 4A, multicellular spheroids were produced using the liquid overlay technique as described in [63]. Briefly, 96-well flat-bottom plates were coated with 60 µl of agarose-DMEM, then loaded with 200 µl of cell suspensions at predefined concentrations (HCT116: 17,500 cells/ml; U-2 OS: 50,000 cells/ml; HBL100: 200,000 cells/ml). Following a 4-day incubation period, spheroids with an average 400 µm diameter were observed. Alternatively, for figure 4B, U-2 OS cell were seeded at 5000 cells per well in a 96-well black ULA plate (Ultra Low Adherence, Corning). After centrifugation at 200g for 10 min, spheroids were allowed to form for 3 days in order to reach 400 µm in diameter.

Compounds were then added at a single dose and half of the medium was changed every 2 days [64]. Cell viability was measured after 2 and 10 days using the CellTiter-Glo® Luminescent Cell Viability Assay (Promega) following the manufacturer's protocol.

5.4 In-cell kinase activity evaluation

For immunofluorescence, cells were grown on glass coverslips, fixed with 4% paraformaldehyde in PBS, permeabilized by 0.15% Triton-X100 for 2 minutes and processed using standard protocols. Images were acquired with a Coolsnap HQ_2 CCD camera (Photometrics) on a Zeiss Axio microscope (Carl Zeiss) using a 100x NA 1.40 objective. Image acquisition and processing were performed using Metamorph (Molecular Device). Deconvolution was performed using the AutoQuant module in Metamorph. Quantification of signal intensity was performed using ImageJ software (NIH). Primary antibodies used in this study included anti-phospho-Thr3 Histone H3 (1/1000 dilution, Millipore).

5.5 Cell cycle analysis

After treatment with the compounds, $0.5\text{-}1.10^6$ cells were trypsinized and washed twice in PBS containing 0.1% F68-pluronic acid. Cells were fixed for 2h in ice-cold ethanol, then washed twice in PBS containing 0.1% F68-pluronic acid, centrifuged at 200 g and resuspended in a PBS buffer containing 1% Triton, 100 µg/ml RNase A (Thermo Scientific) and 40 µg/ml propidium iodide (Life Technologies). DNA content was determined using a FACS Canto II (BD) and the data were analyzed using FCS Express Cytometry software (De Novo Software). Twenty thousand events were collected in each run.

5.6 Migration assay

U-2 OS cells were seeded in 96-well plates (ZOOM-image Lock, Essen Bioscience) in DMEM medium containing 0.5% FBS. Cell-free zones were created by scratch wounds in cell monolayers and gently washed before addition of the compounds or DMSO for the control culture (assays were conducted in quadruplicate). The assay was then initiated by image acquisition (every hour) in real time by microscopy using an IncuCyte system (Essen Bioscience). The Relative Wound Density (RWD) was used as the metric to estimate the cellular migration.

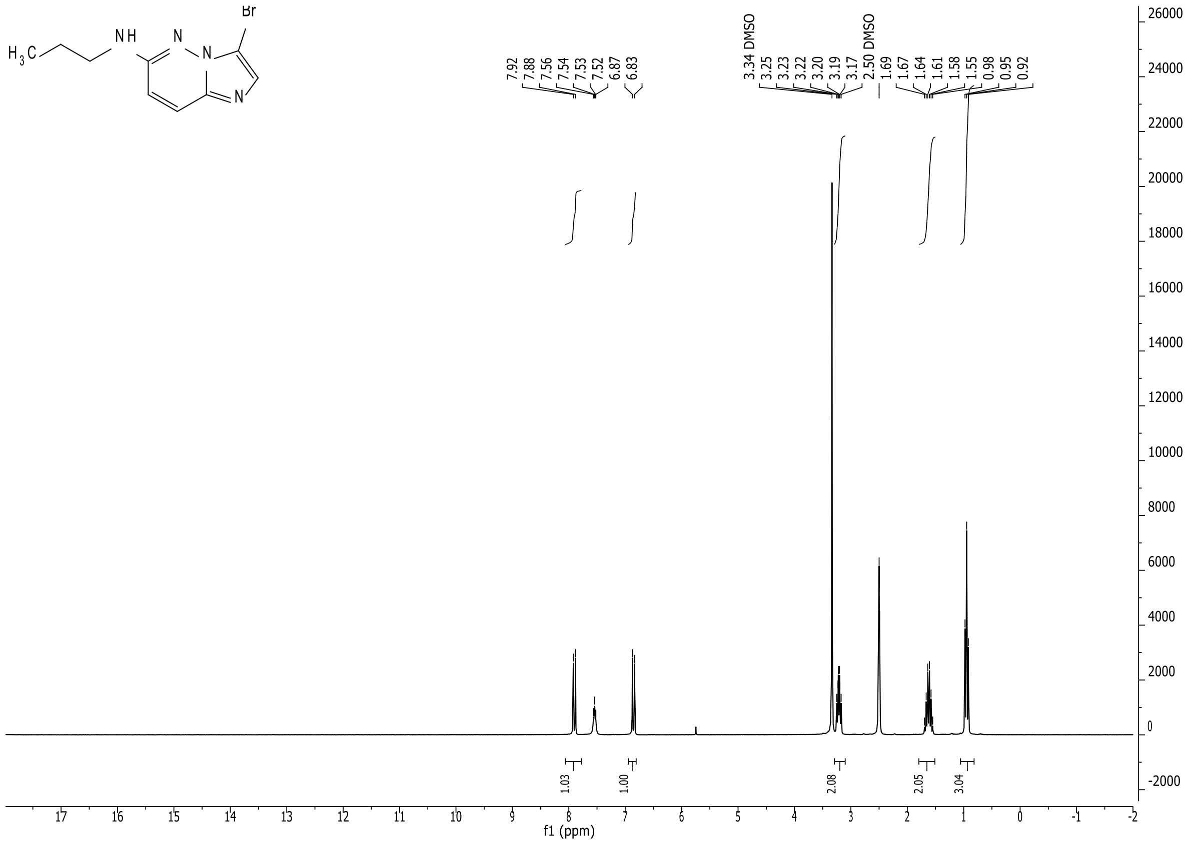

Br
NH
N
N
N
H_3C
7.92
7.88
7.56
7.54
7.53
7.52
6.87
6.83
3.34 DMSO
3.25
3.23
3.22
3.20
3.19
3.17
2.50 DMSO
1.69
1.67
1.64
1.61
1.58
1.55
0.98
0.95
0.92
1.03
1.00
2.08
2.05
3.04
f1 (ppm)

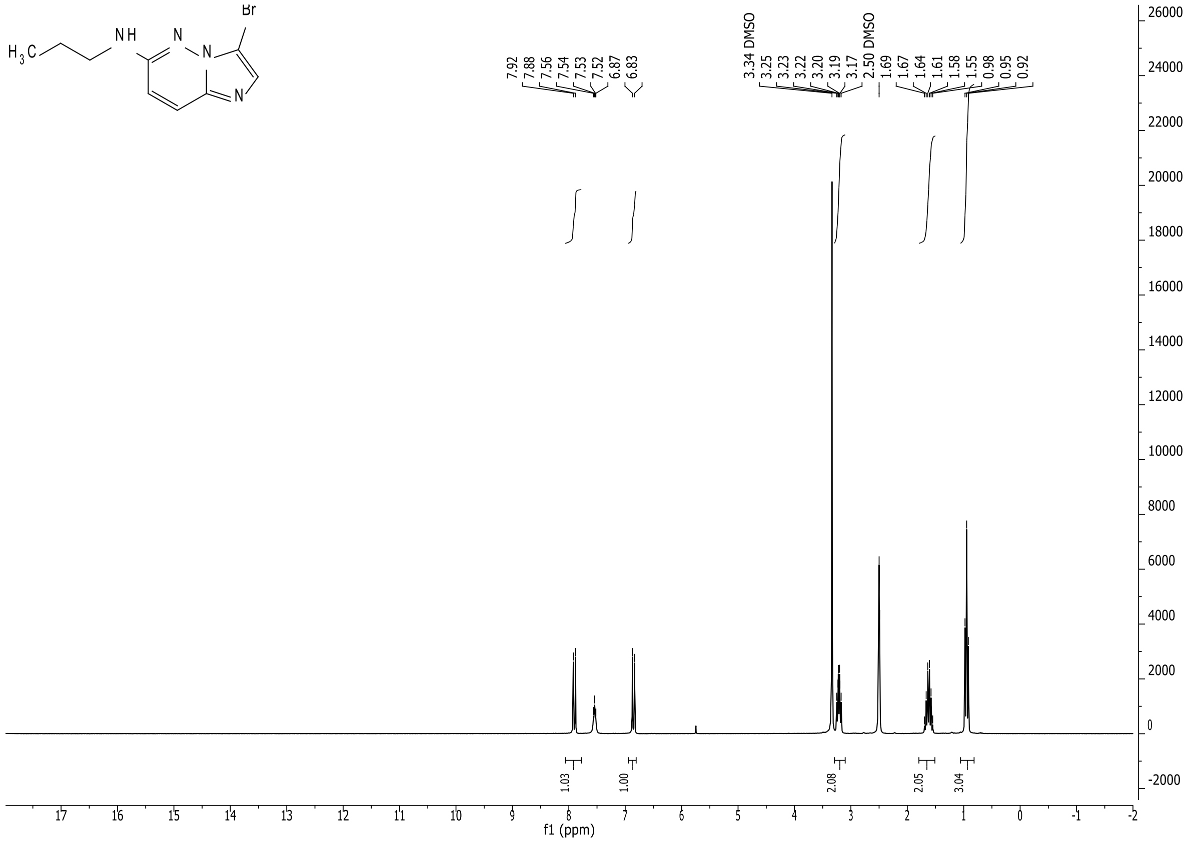

Br
NH
N
N
N
H_3C
7.92
7.88
7.56
7.54
7.53
7.52
6.87
6.83
3.34 DMSO
3.25
3.23
3.22
3.20
3.19
3.17
2.50 DMSO
1.69
1.67
1.64
1.61
1.58
1.55
0.98
0.95
0.92
1.03
1.00
2.08
2.05
3.04
f1 (ppm)

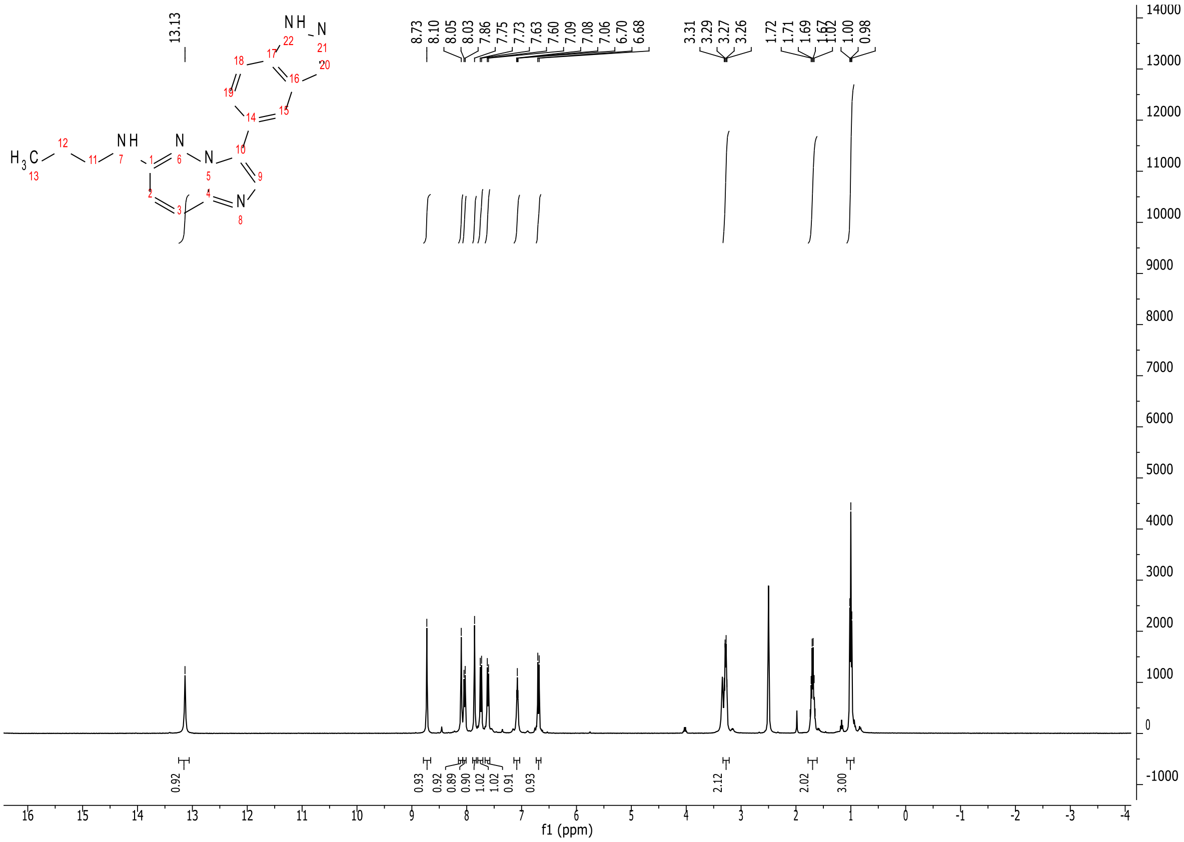
13.13
8.73
8.10
8.05
8.03
7.86
7.75
7.73
7.63
7.60
7.09
7.08
7.06
6.70
6.68
3.31
3.29
3.27
3.26
1.72
1.71
1.69
1.67
1.02
1.00
0.98
0.92
0.93
0.92
0.89
0.90
1.02
1.02
0.91
0.93
2.12
2.02
3.00
f1 (ppm)

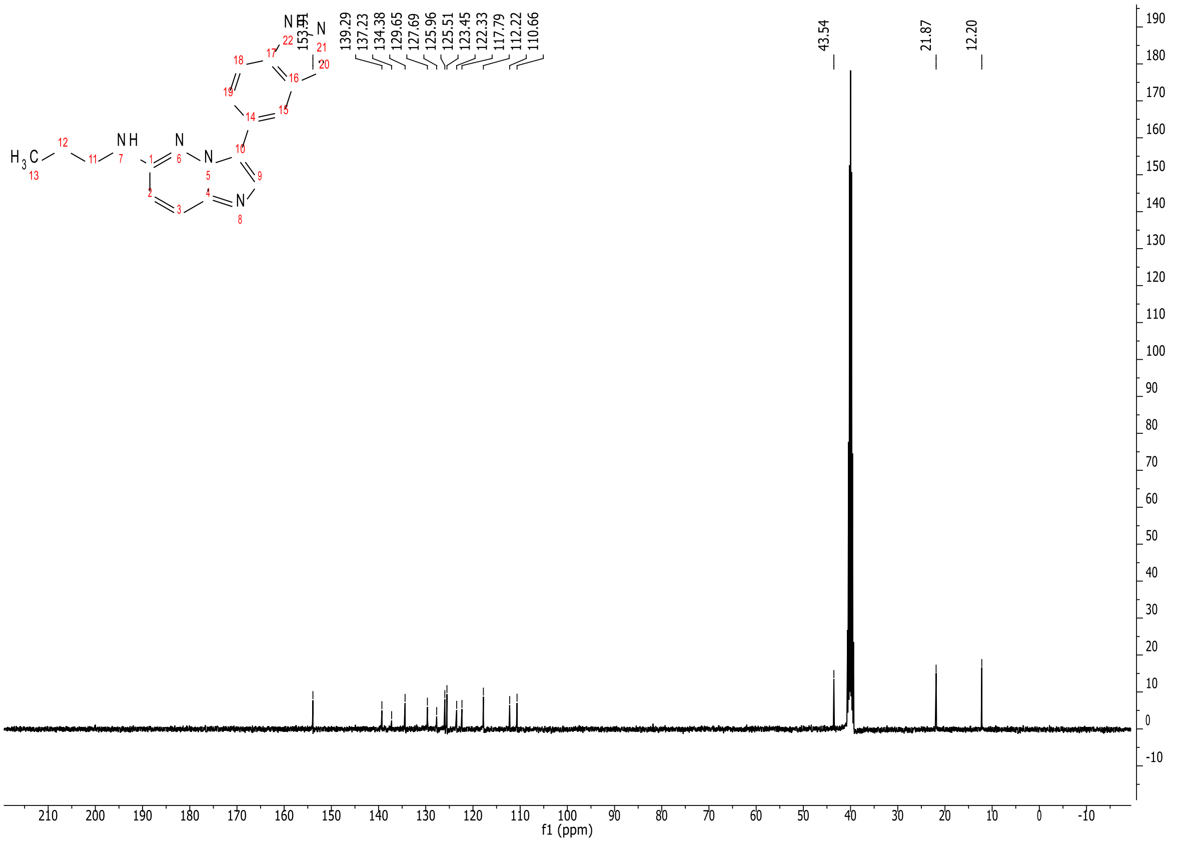
153.91
139.29
137.23
134.38
129.65
127.69
125.96
125.51
123.45
122.33
117.79
112.22
110.66
43.54
21.87
12.20
f1 (ppm)

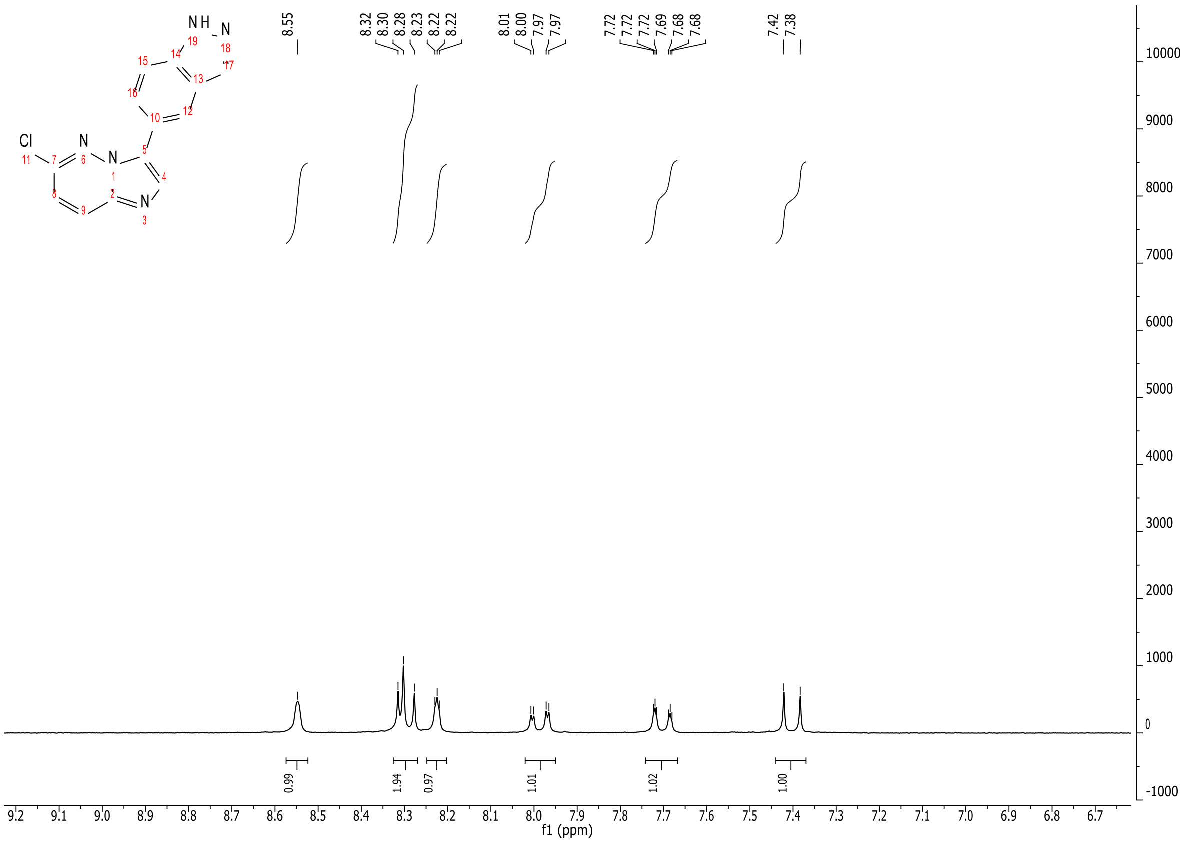
8.55
8.32
8.30
8.28
8.23
8.22
8.22
8.01
8.00
7.97
7.97
7.72
7.72
7.72
7.69
7.68
7.68
7.42
7.38
0.99
1.94
0.97
1.01
1.02
1.00
f1 (ppm)

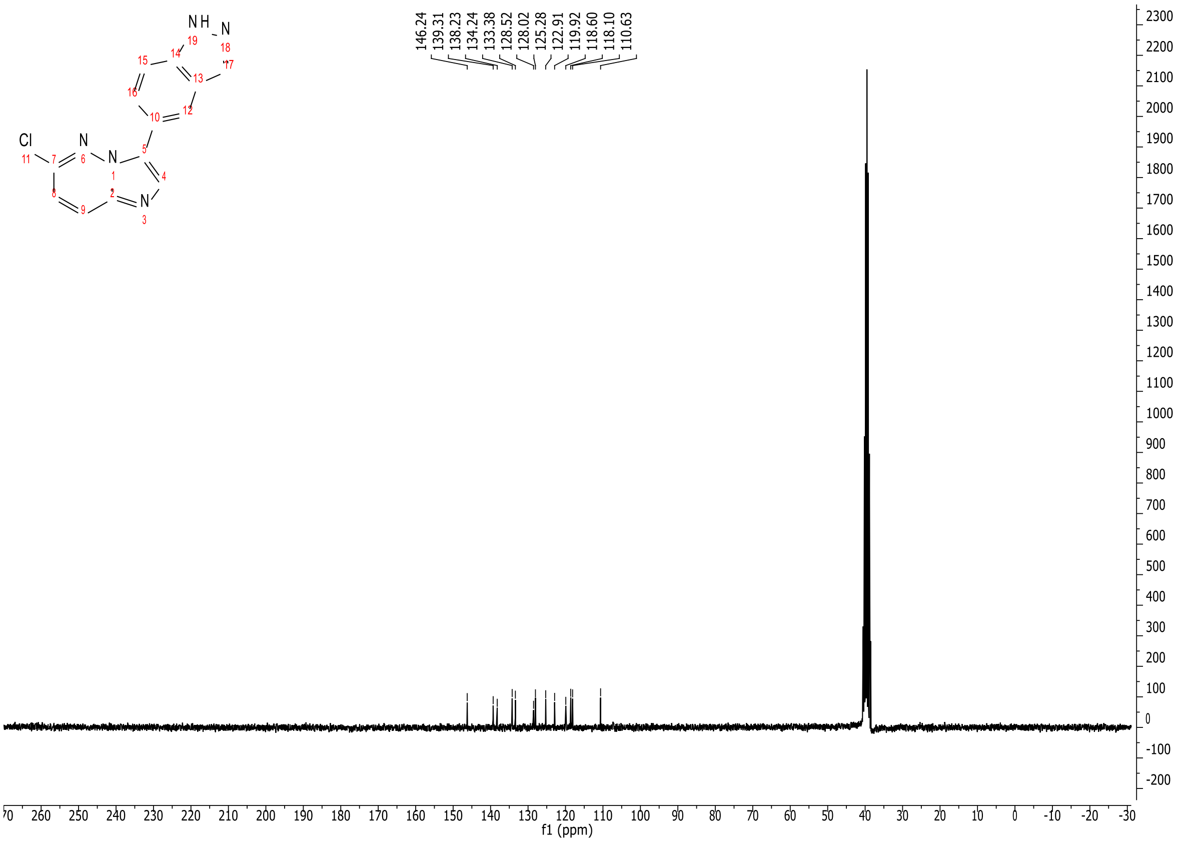
146.24
139.31
138.23
134.24
133.38
128.52
128.02
125.28
122.91
119.92
118.60
118.10
110.63
f1 (ppm)

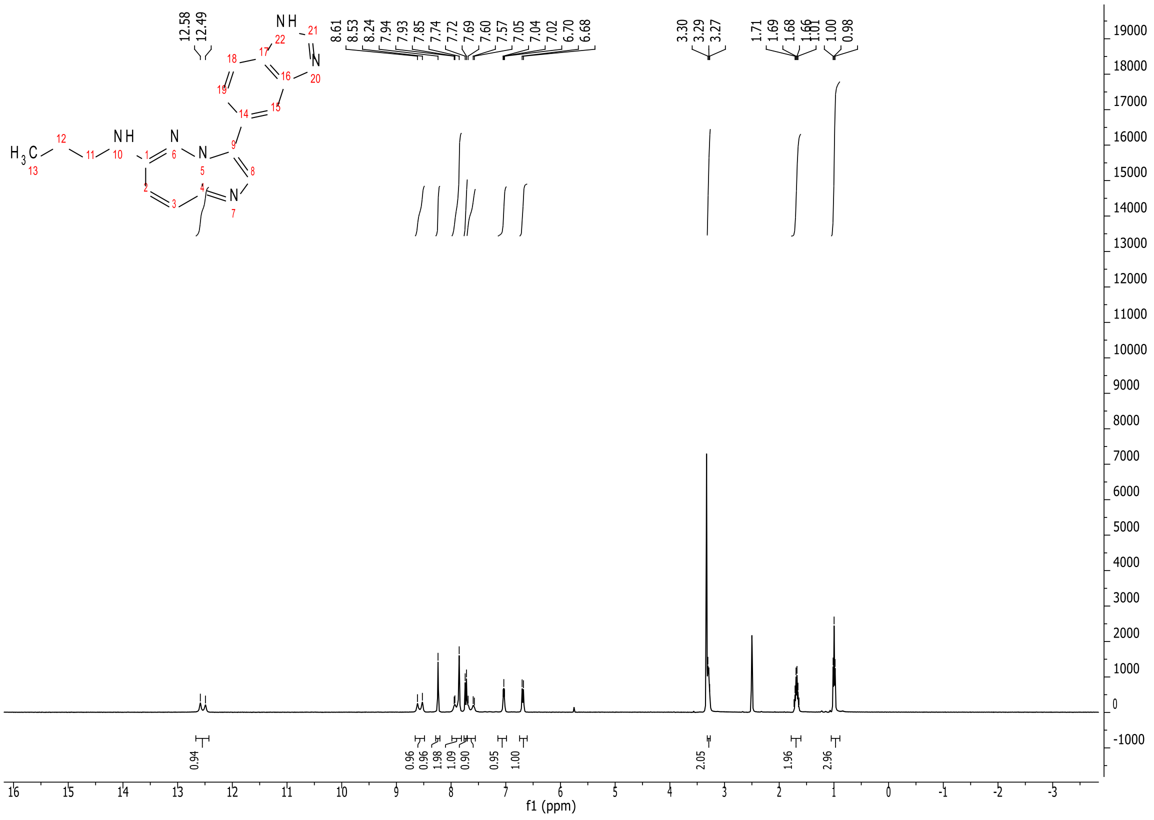
12.58
12.49
8.61
8.53
8.24
7.94
7.93
7.85
7.74
7.72
7.69
7.60
7.57
7.05
7.04
7.02
6.70
6.68
3.30
3.29
3.27
1.71
1.69
1.68
1.00
0.98
0.94
0.96
0.96
1.98
1.09
0.90
0.95
1.00
2.05
1.96
2.96
f1 (ppm)

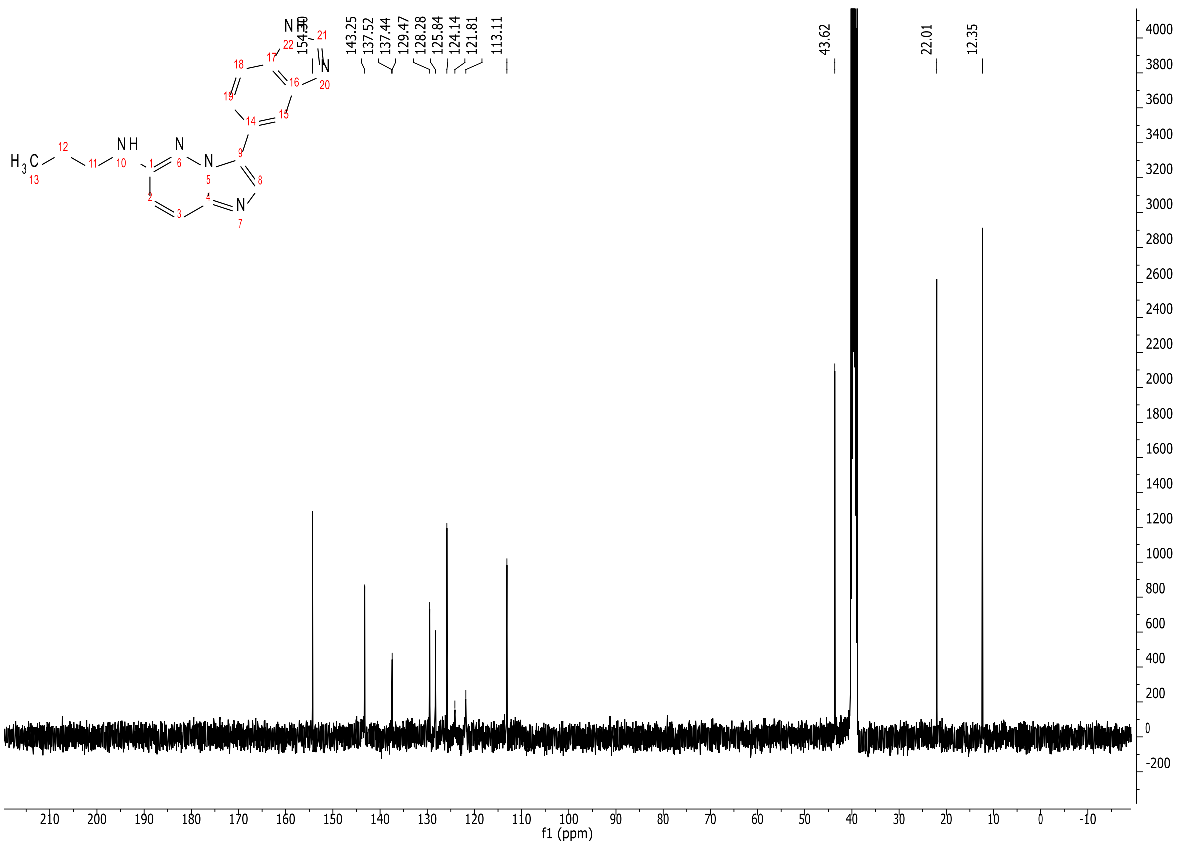
154.30
143.25
137.52
137.44
129.47
128.28
125.84
124.14
121.81
113.11
43.62
22.01
12.35
f1 (ppm)

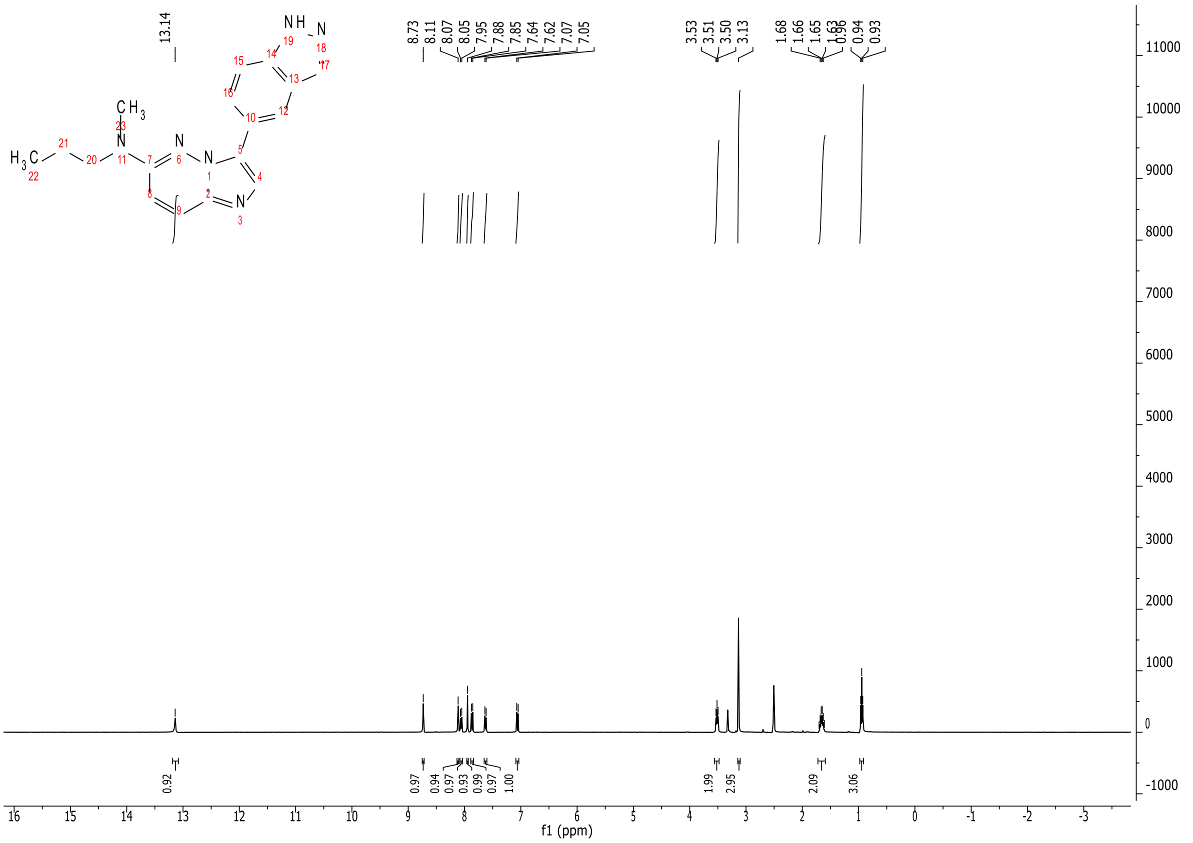
13.14
8.73
8.11
8.07
8.05
7.95
7.88
7.85
7.64
7.62
7.07
7.05
3.53
3.51
3.50
3.13
1.68
1.66
1.65
1.63
0.96
0.94
0.93
f1 (ppm)

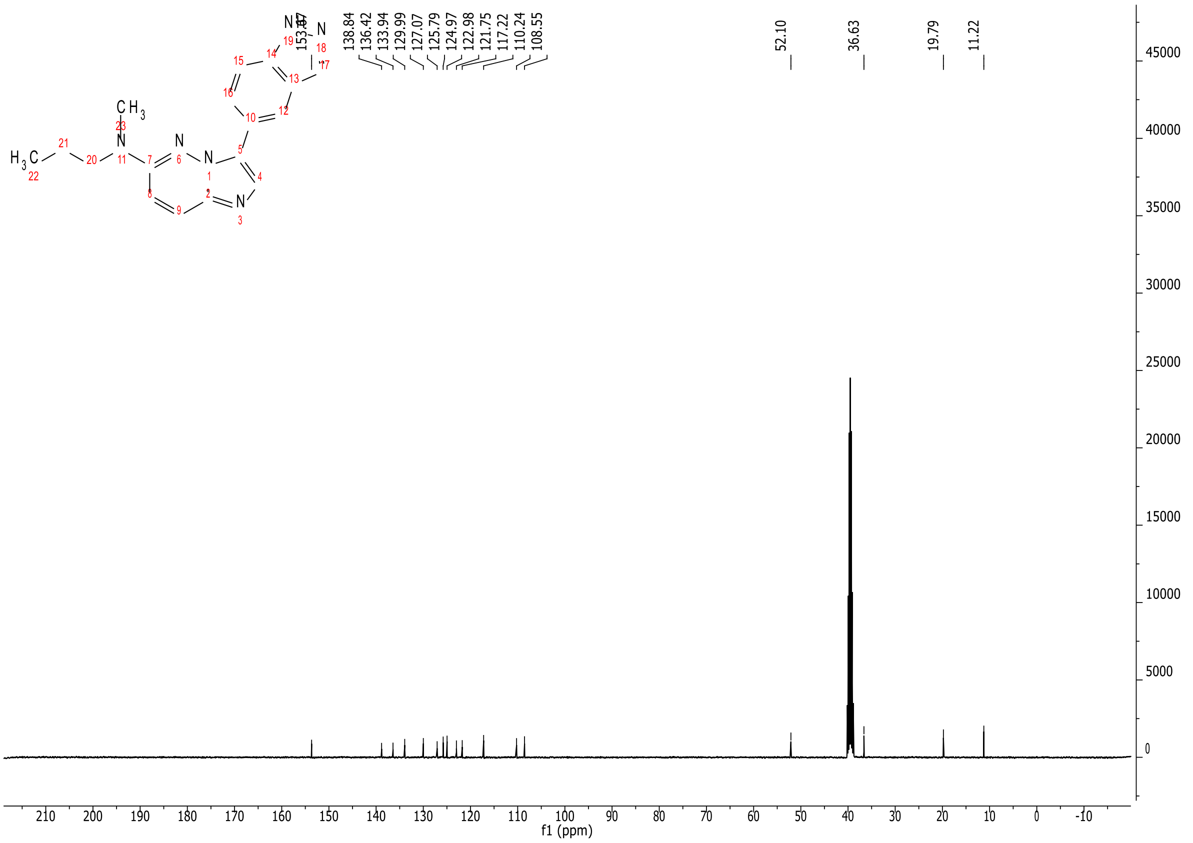
153.47
138.84
136.42
133.94
129.99
127.07
125.79
124.97
122.98
121.75
117.22
110.24
108.55
52.10
36.63
19.79
11.22
f1 (ppm)

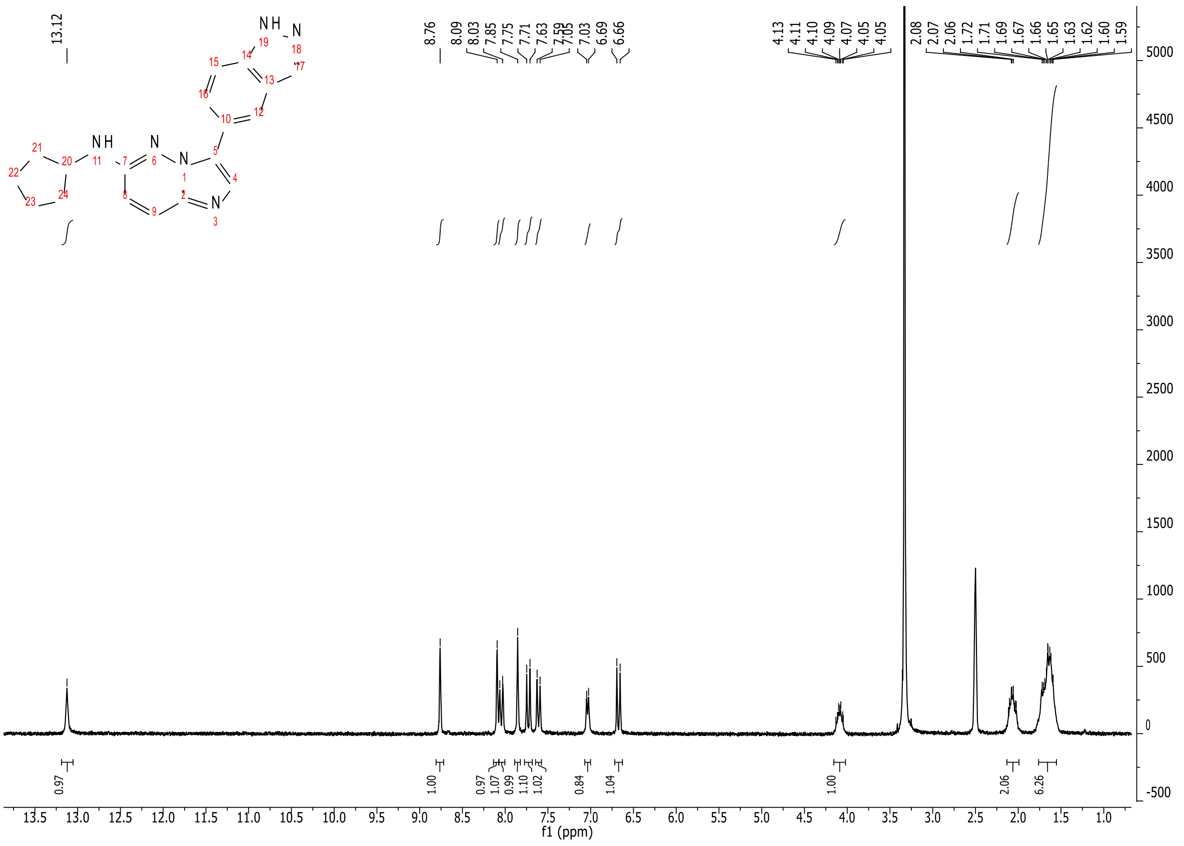
13.12
8.76
8.09
8.03
7.85
7.75
7.71
7.63
7.59
7.05
7.03
6.69
6.66
4.13
4.11
4.10
4.09
4.07
4.05
4.05
2.08
2.07
2.06
1.72
1.71
1.69
1.67
1.66
1.65
1.63
1.62
1.60
1.59
0.97
1.00
0.97
1.07
0.99
1.10
1.02
0.84
1.04
1.00
2.06
6.26
f1 (ppm)

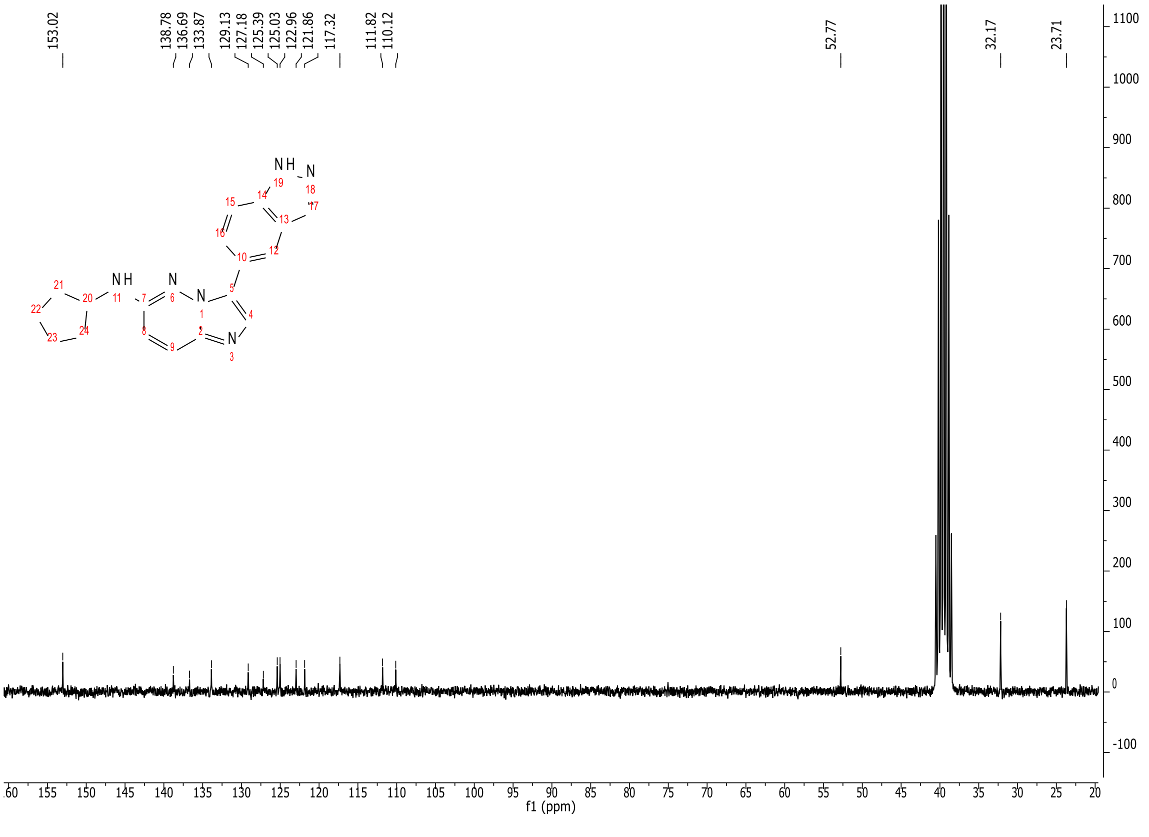
153.02
138.78
136.69
133.87
129.13
127.18
125.39
125.03
122.96
121.86
117.32
111.82
110.12
52.77
32.17
23.71
f1 (ppm)

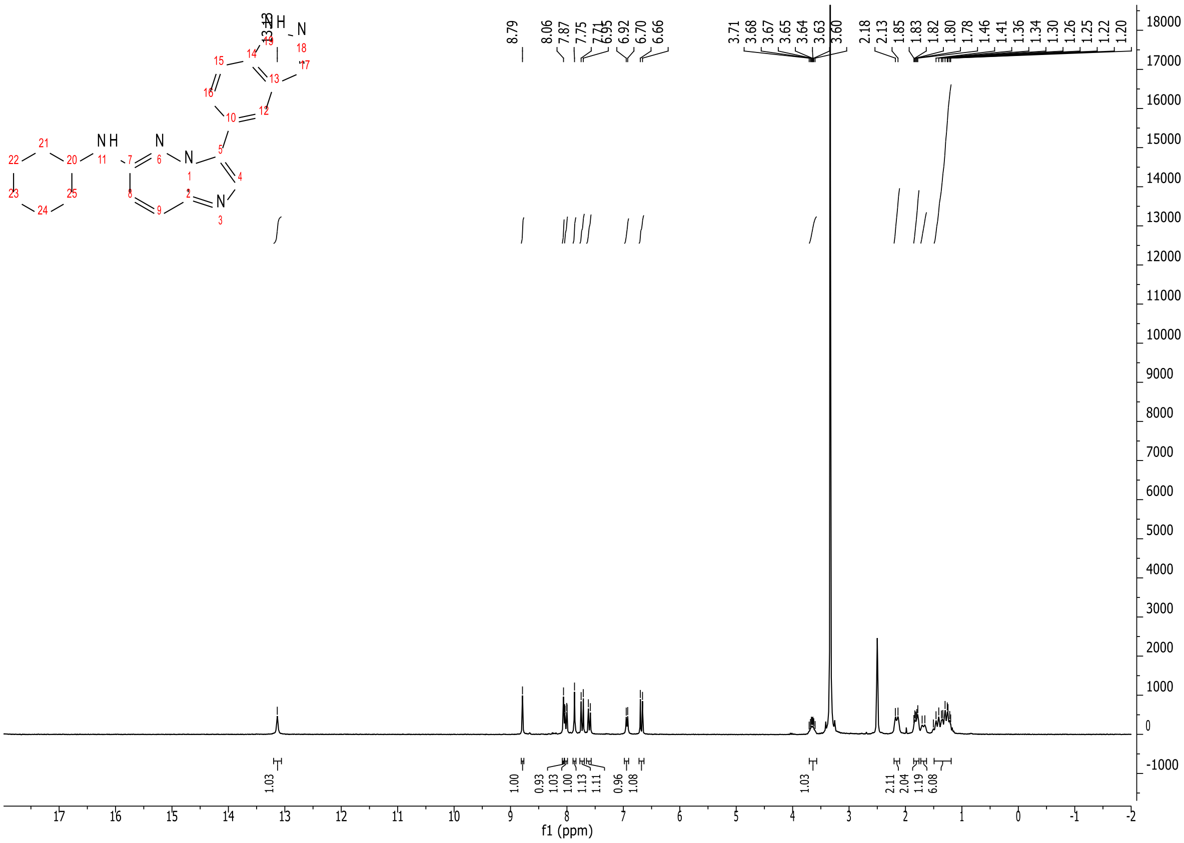
f1 (ppm)

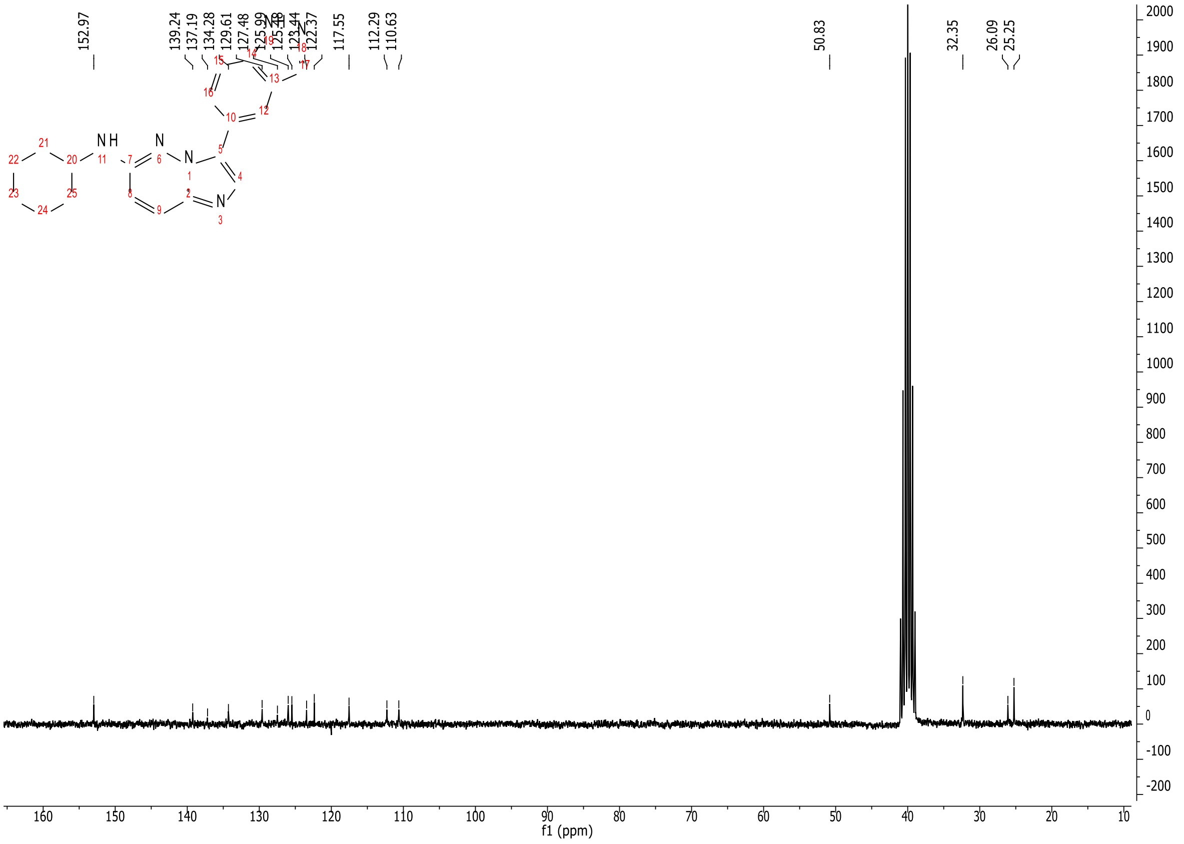
f1 (ppm)

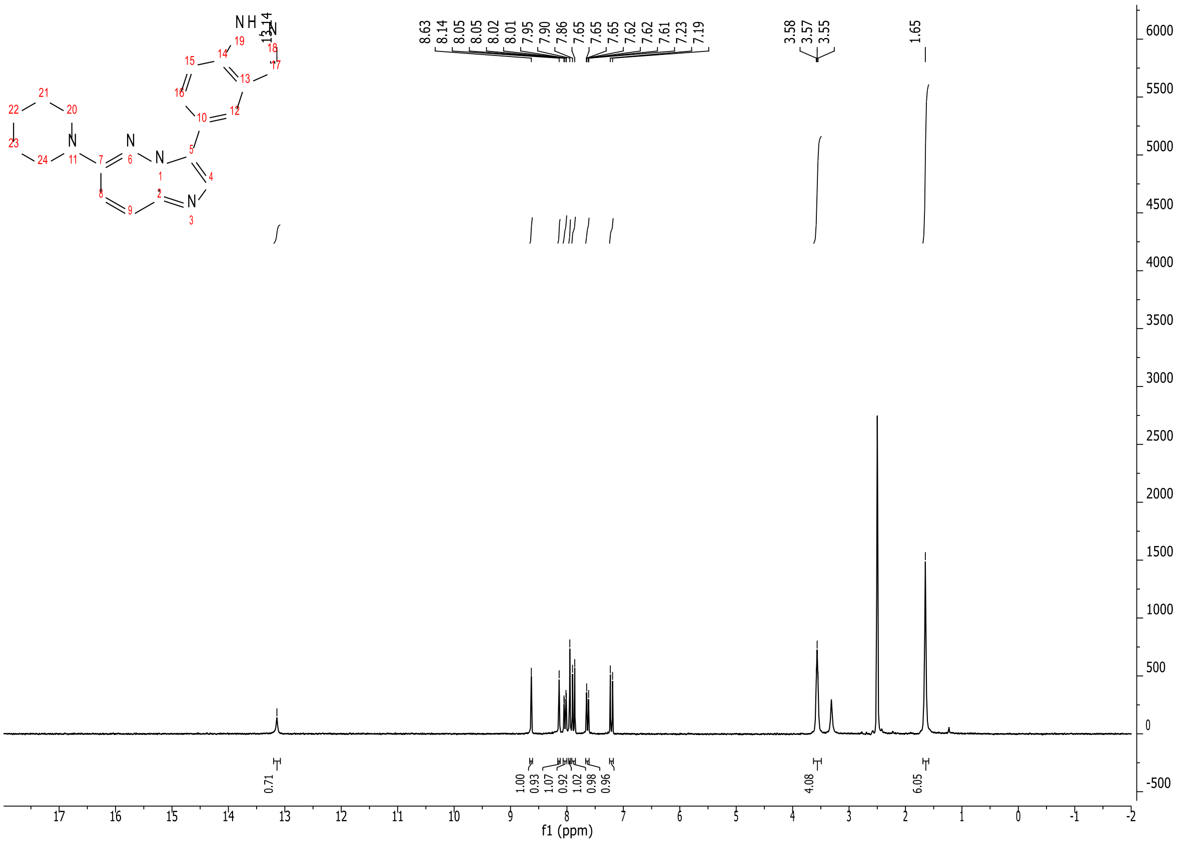

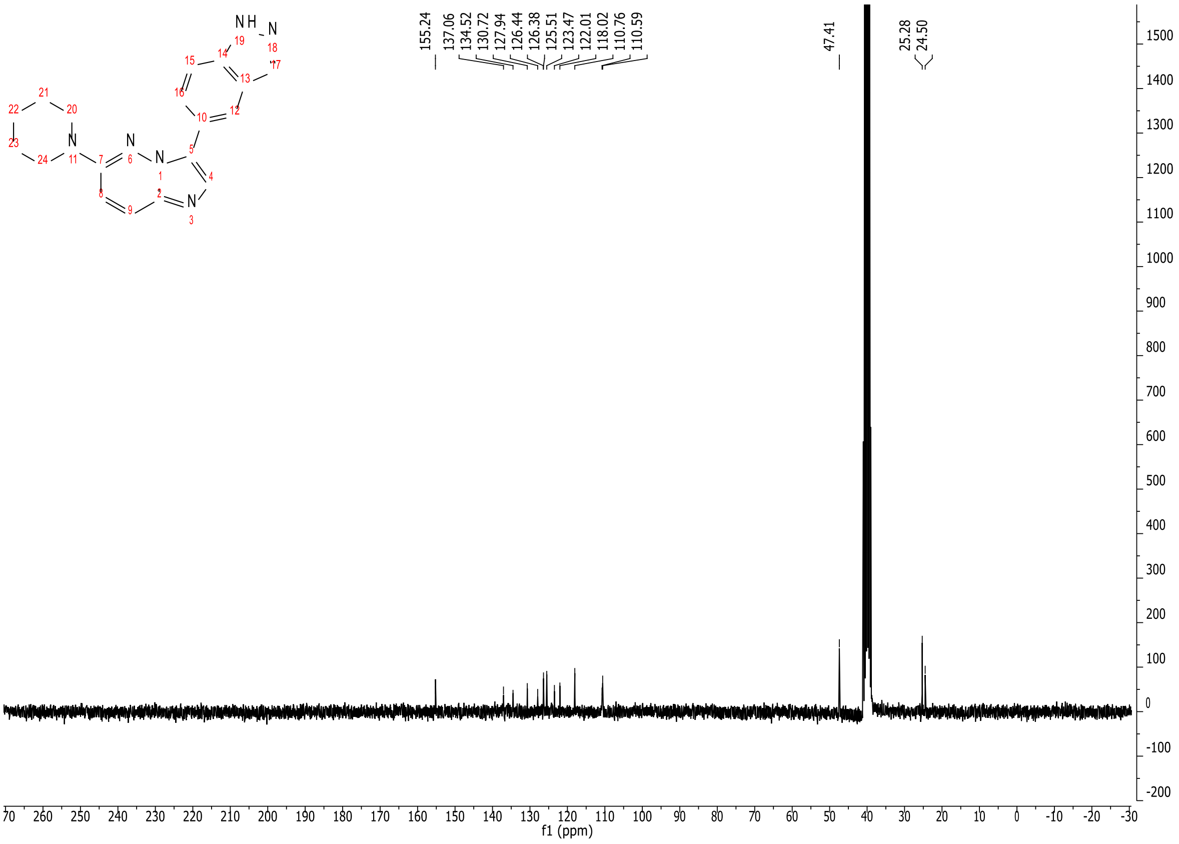

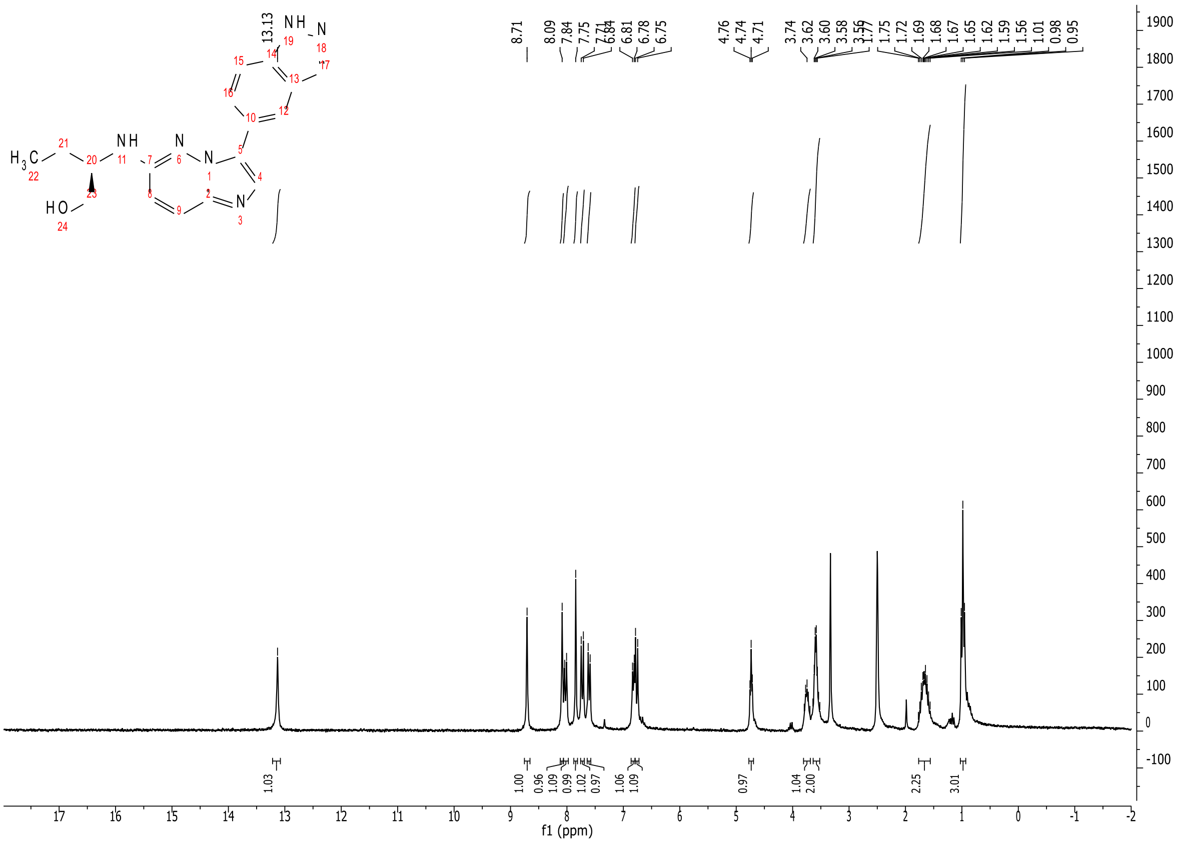

13.13
8.71
8.09
7.84
7.75
7.71
6.84
6.81
6.78
6.75
4.76
4.74
4.71
3.74
3.62
3.60
3.58
3.56
3.37
1.75
1.72
1.69
1.68
1.67
1.65
1.62
1.59
1.56
1.01
0.98
0.95
1.03
1.00
0.96
1.09
0.99
1.02
0.97
1.06
1.09
0.97
1.04
2.00
2.25
3.01
f1 (ppm)

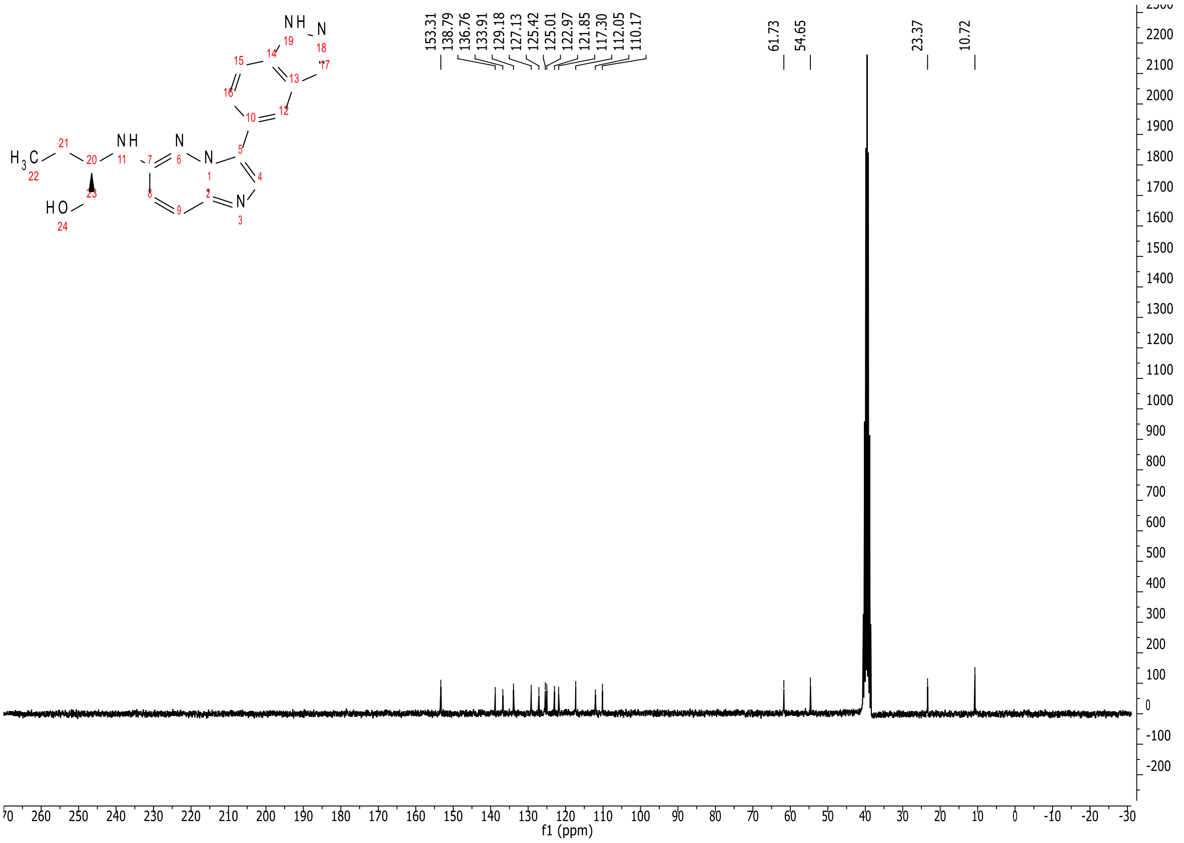

153.31
138.79
136.76
133.91
129.18
127.13
125.42
125.01
122.97
121.85
117.30
112.05
110.17
61.73
54.65
23.37
10.72
f1 (ppm)

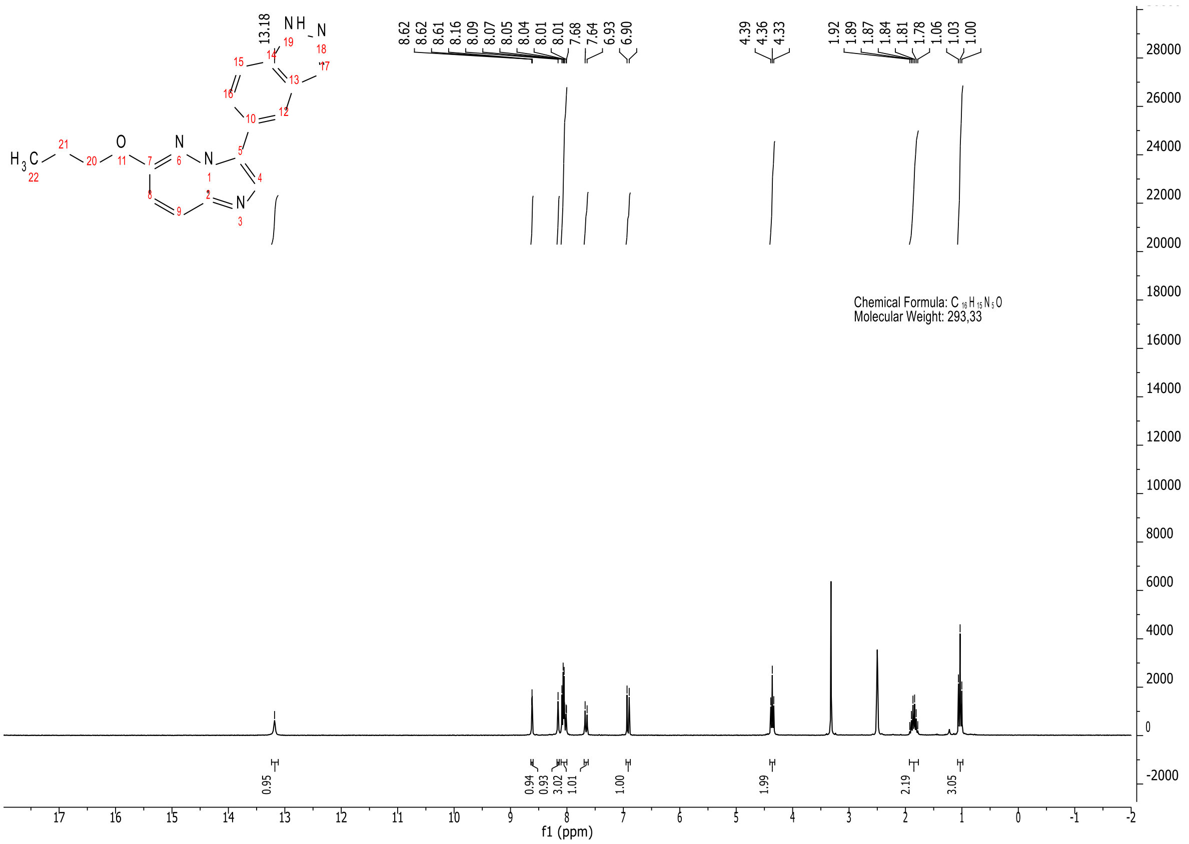
13.18
8.62 8.62 8.61 8.16 8.09 8.07 8.05 8.04 8.01 8.01 7.68 7.64 6.93 6.90
4.39 4.36 4.33
1.92 1.89 1.87 1.84 1.81 1.78 1.06 1.03 1.00
Chemical Formula: C16H15N5O
Molecular Weight: 293,33
0.95 0.94 0.93 3.02 1.01 1.00 1.99 2.19 3.05
f1 (ppm)

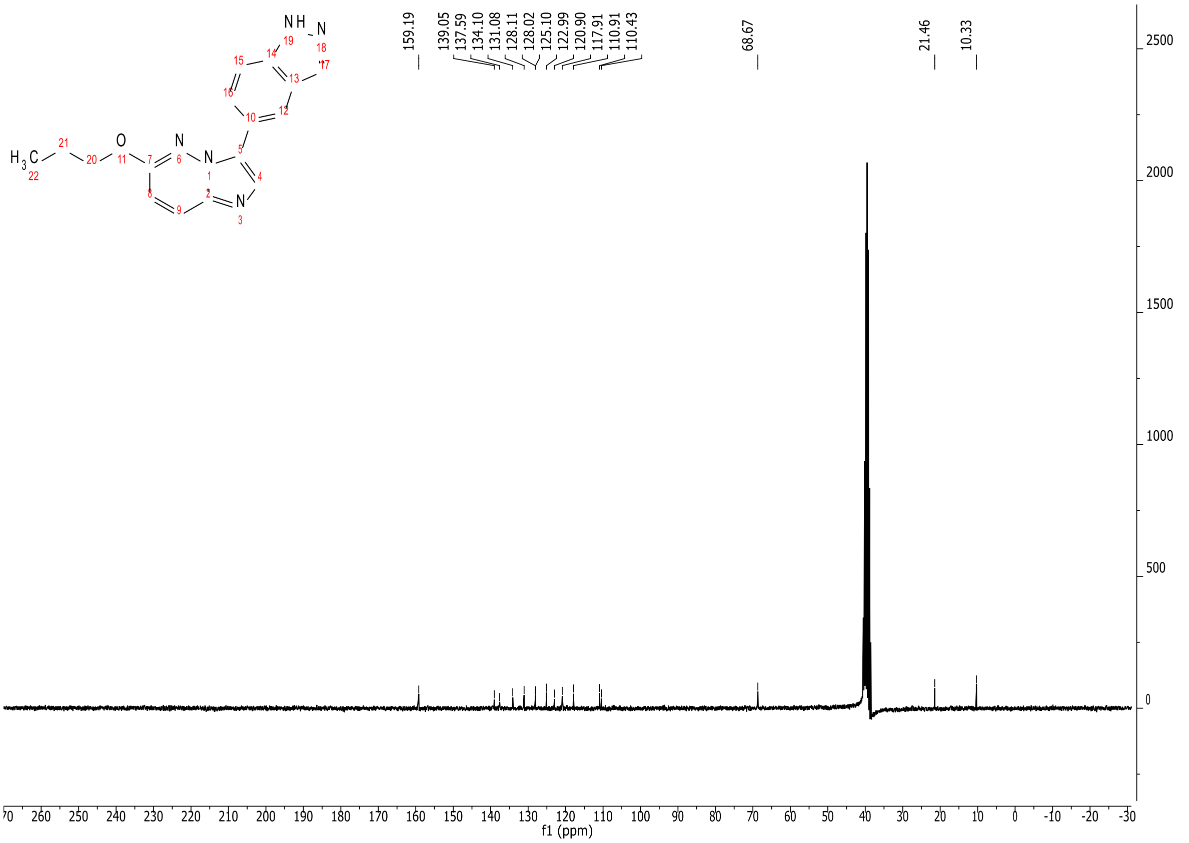
159.19
139.05 137.59 134.10 131.08 128.11 128.02 125.10 122.99 120.90 117.91 110.91 110.43
68.67
21.46
10.33
f1 (ppm)

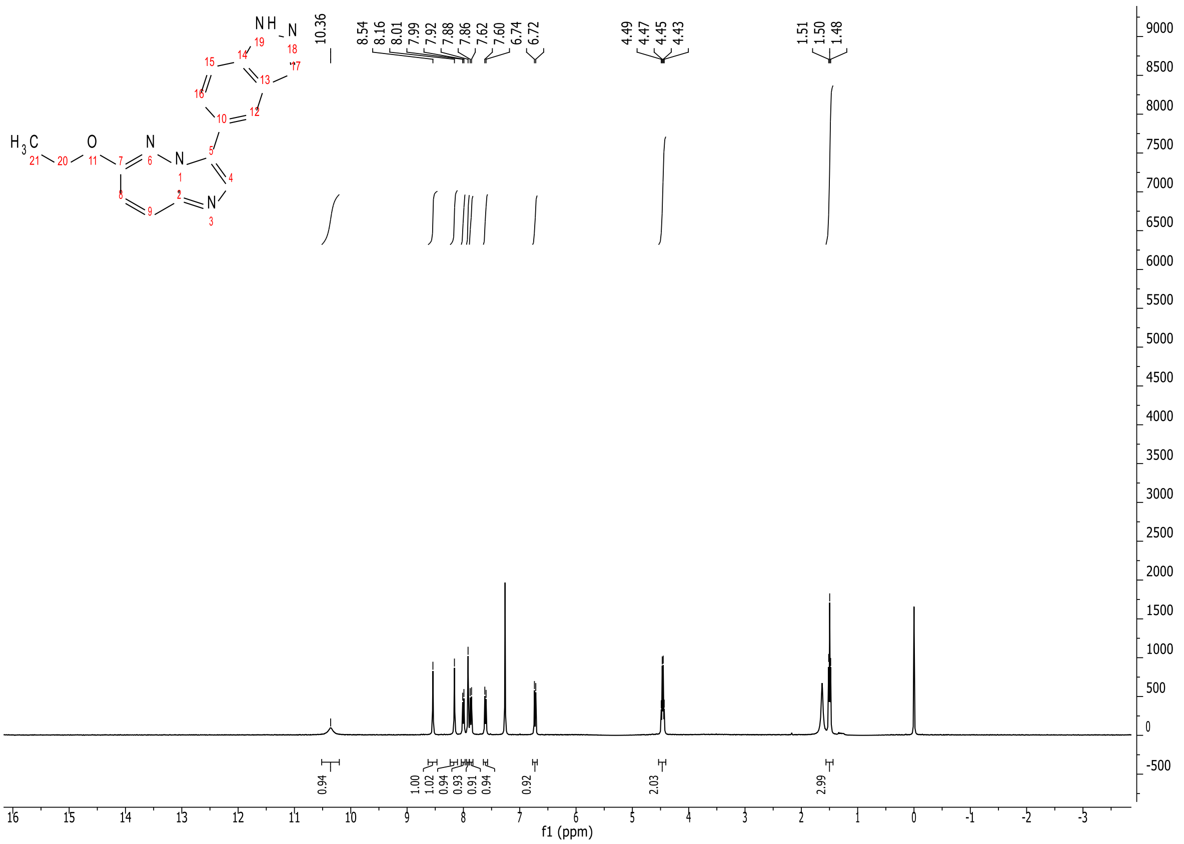
10.36
8.54
8.16
8.01
7.99
7.92
7.88
7.86
7.62
7.60
6.74
6.72
4.49
4.47
4.45
4.43
1.51
1.50
1.48
0.94
1.00
1.02
0.94
0.93
0.91
0.94
0.92
2.03
2.99
f1 (ppm)

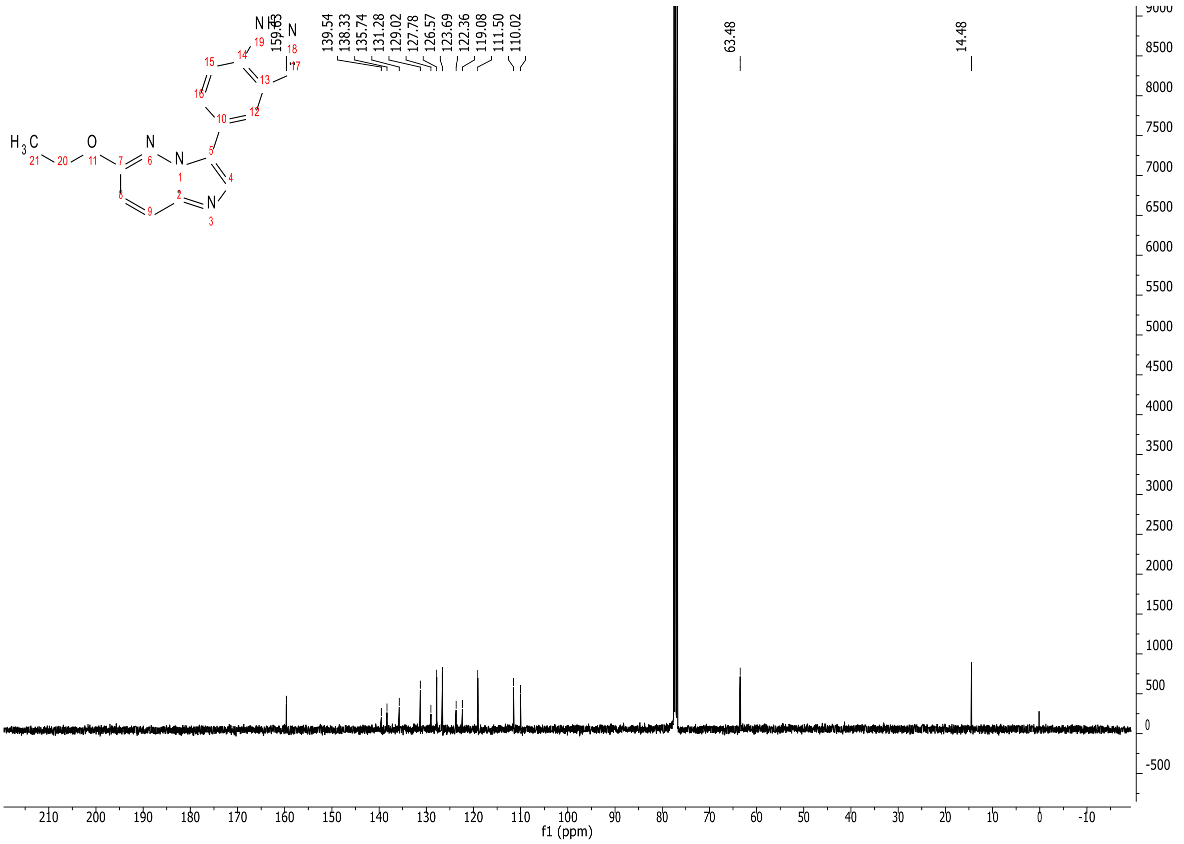
159.65
139.54
138.33
135.74
131.28
129.02
127.78
126.57
123.69
122.36
119.08
111.50
110.02
63.48
14.48
f1 (ppm)

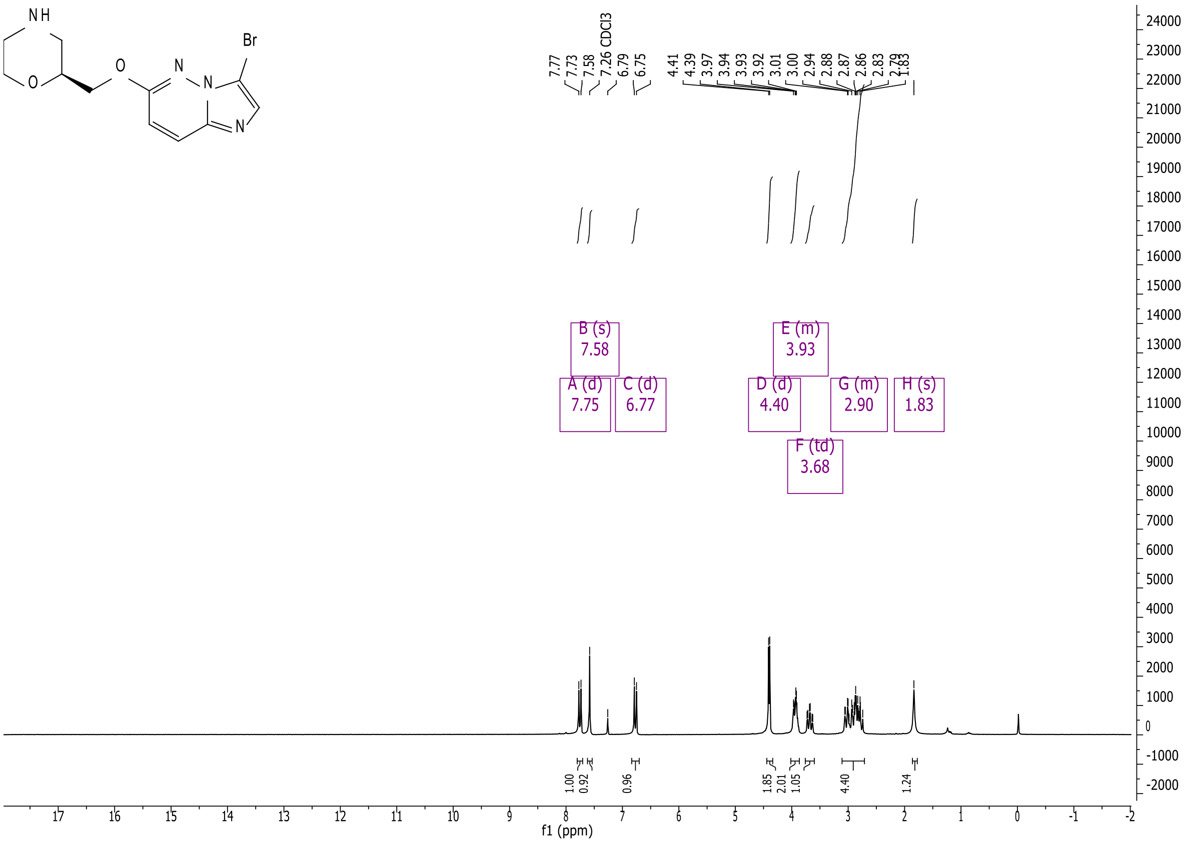
NH
O
O
N
N
N
Br
7.77
7.73
7.58
7.26 CDCl3
6.79
6.75
4.41
4.39
3.97
3.94
3.93
3.92
3.01
3.00
2.94
2.88
2.87
2.86
2.83
2.79
1.83
B (s) 7.58
E (m) 3.93
A (d) 7.75
C (d) 6.77
D (d) 4.40
G (m) 2.90
H (s) 1.83
F (td) 3.68
1.00
0.92
0.96
1.85
2.01
1.05
4.40
1.24
f1 (ppm)

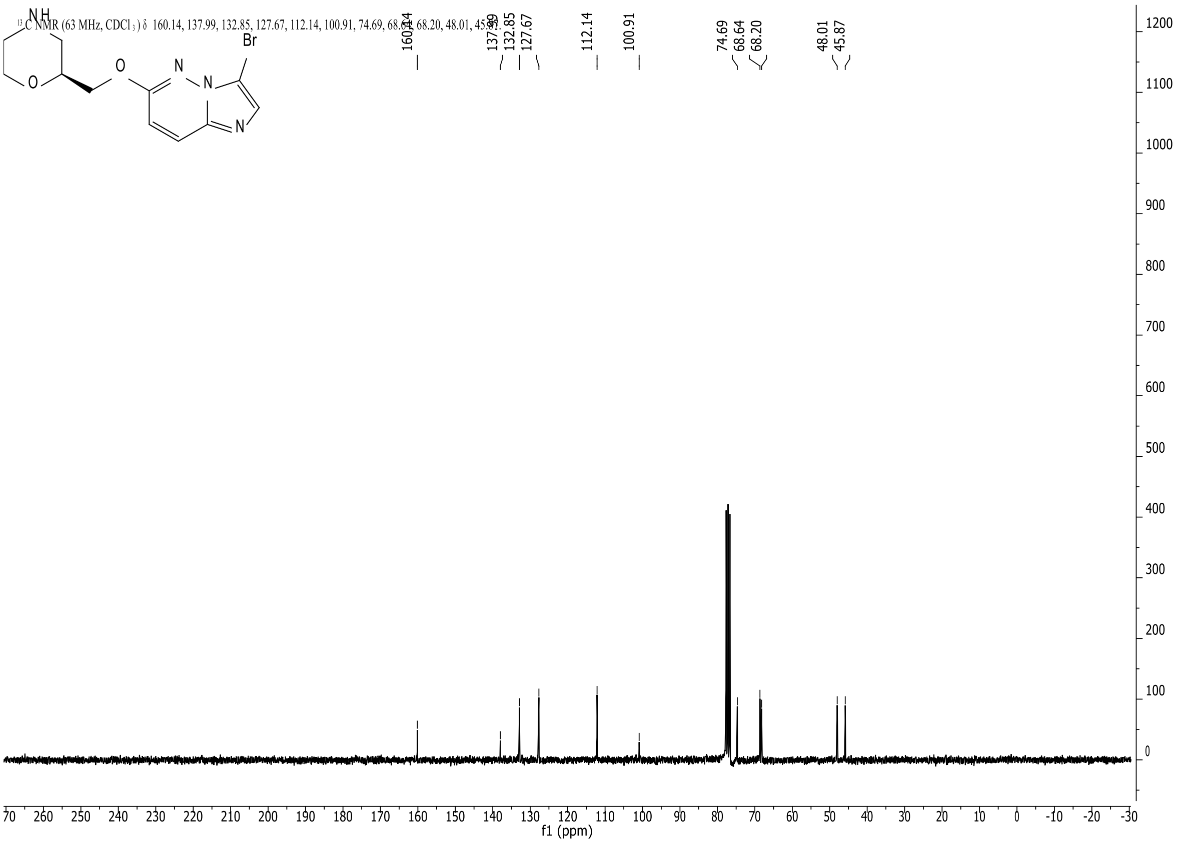
13C NMR (63 MHz, CDCl3) δ 160.14, 137.99, 132.85, 127.67, 112.14, 100.91, 74.69, 68.64, 68.20, 48.01, 45.87.
NH
O
O
N
N
N
Br
160.14
137.99
132.85
127.67
112.14
100.91
74.69
68.64
68.20
48.01
45.87
f1 (ppm)

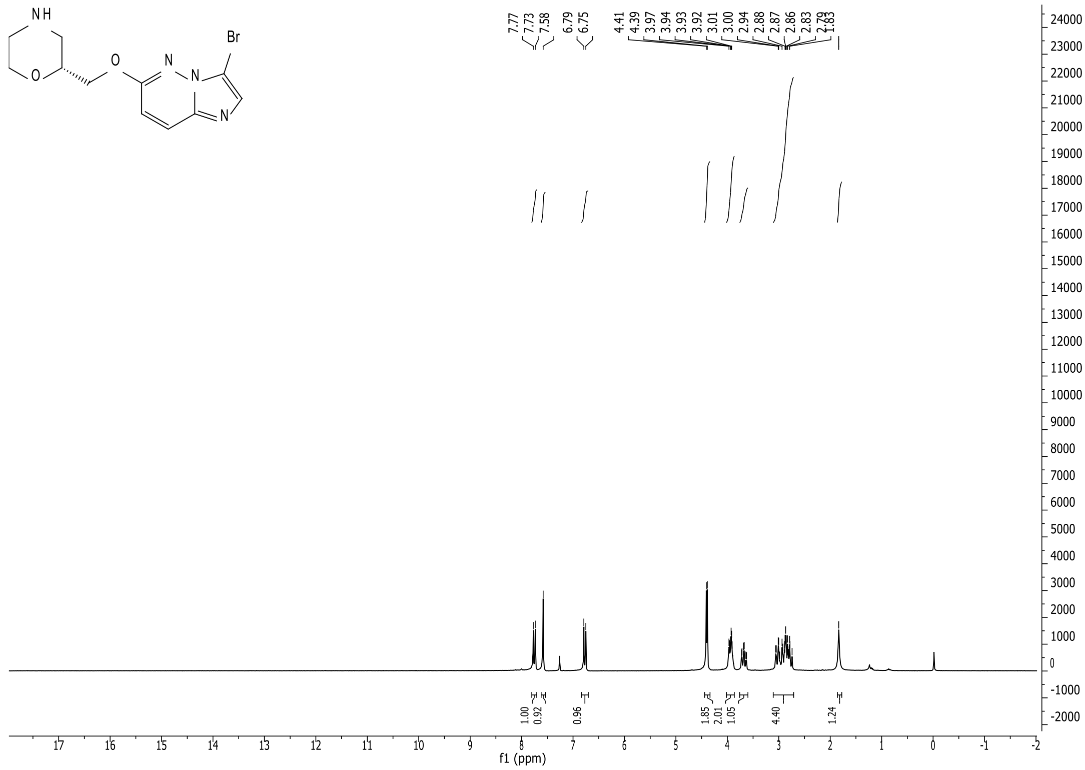
NH
O
N
Br
7.77
7.73
7.58
6.79
6.75
4.41
4.39
3.97
3.94
3.93
3.92
3.01
3.00
2.94
2.88
2.87
2.86
2.83
1.83
1.00
0.92
0.96
1.85
2.01
1.05
4.40
1.24
f1 (ppm)

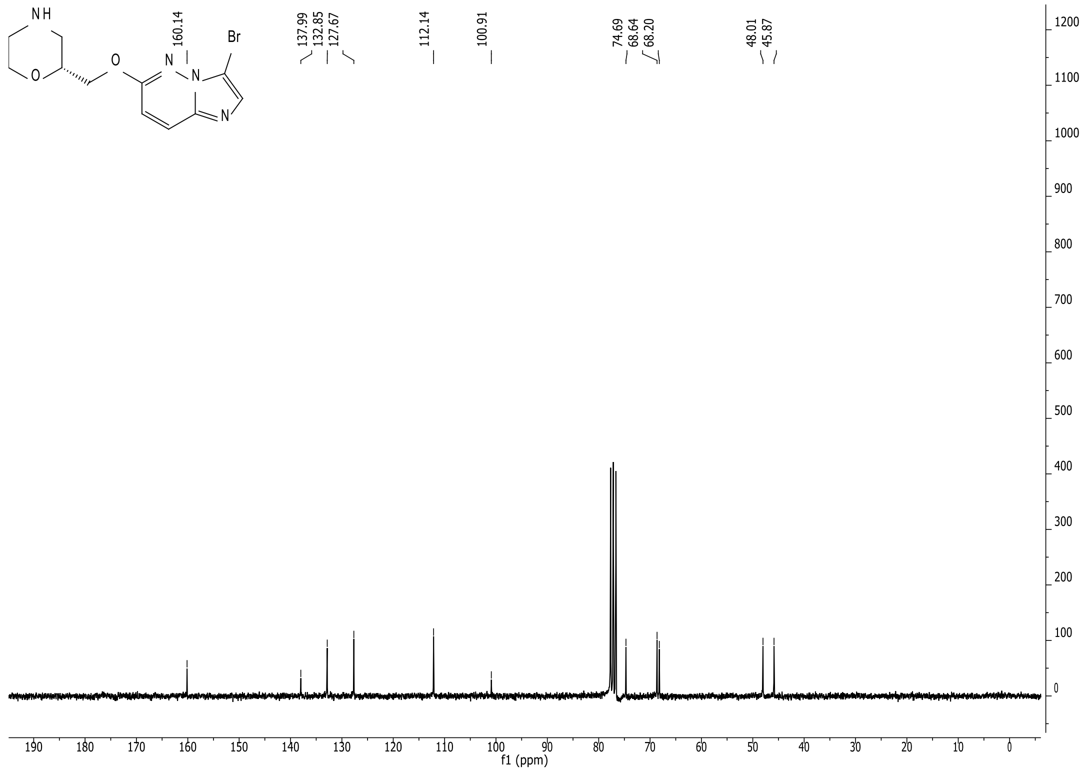
NH
O
N
Br
160.14
137.99
132.85
127.67
112.14
100.91
74.69
68.64
68.20
48.01
45.87
f1 (ppm)

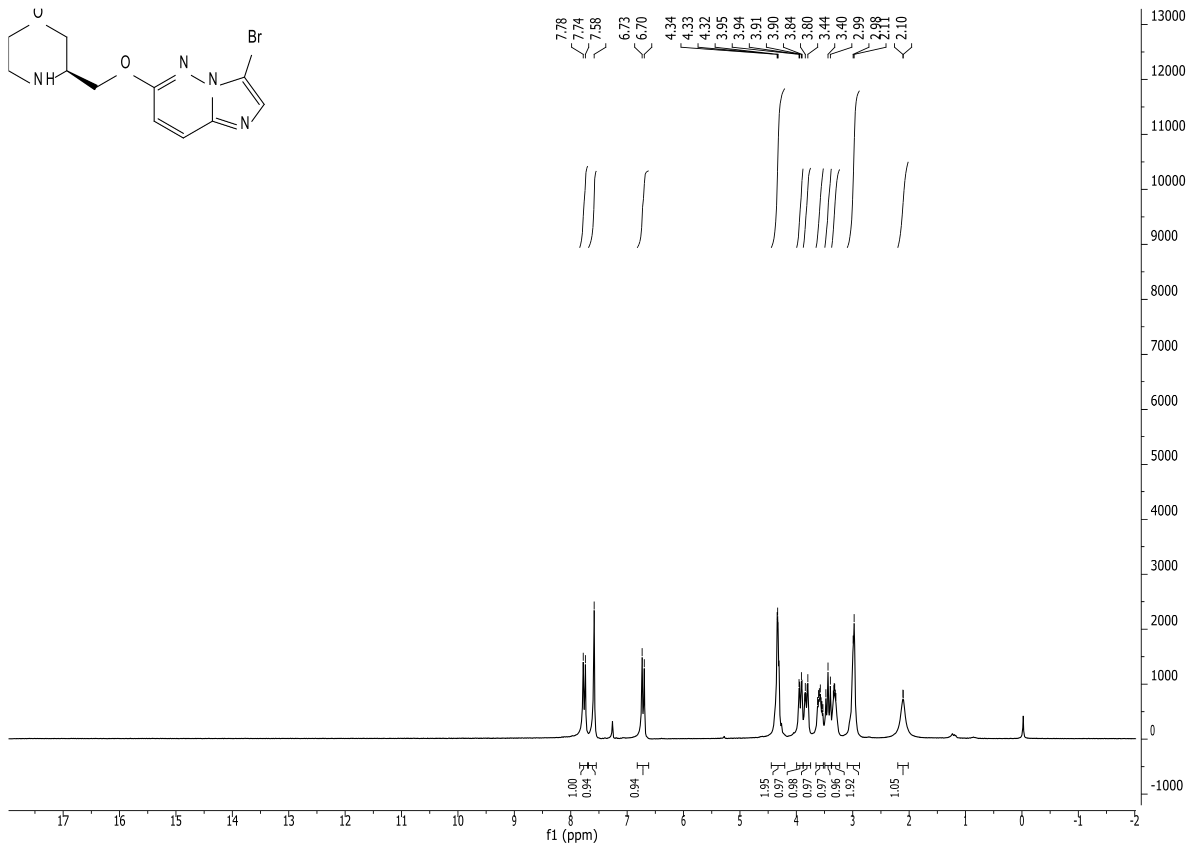
Br
O
N
NH
7.78
7.74
7.58
6.73
6.70
4.34
4.33
4.32
3.95
3.94
3.91
3.90
3.84
3.80
3.44
3.40
2.99
2.98
2.11
2.10
1.00
0.94
0.94
1.95
0.97
0.98
0.97
0.97
0.96
1.92
1.05
f1 (ppm)

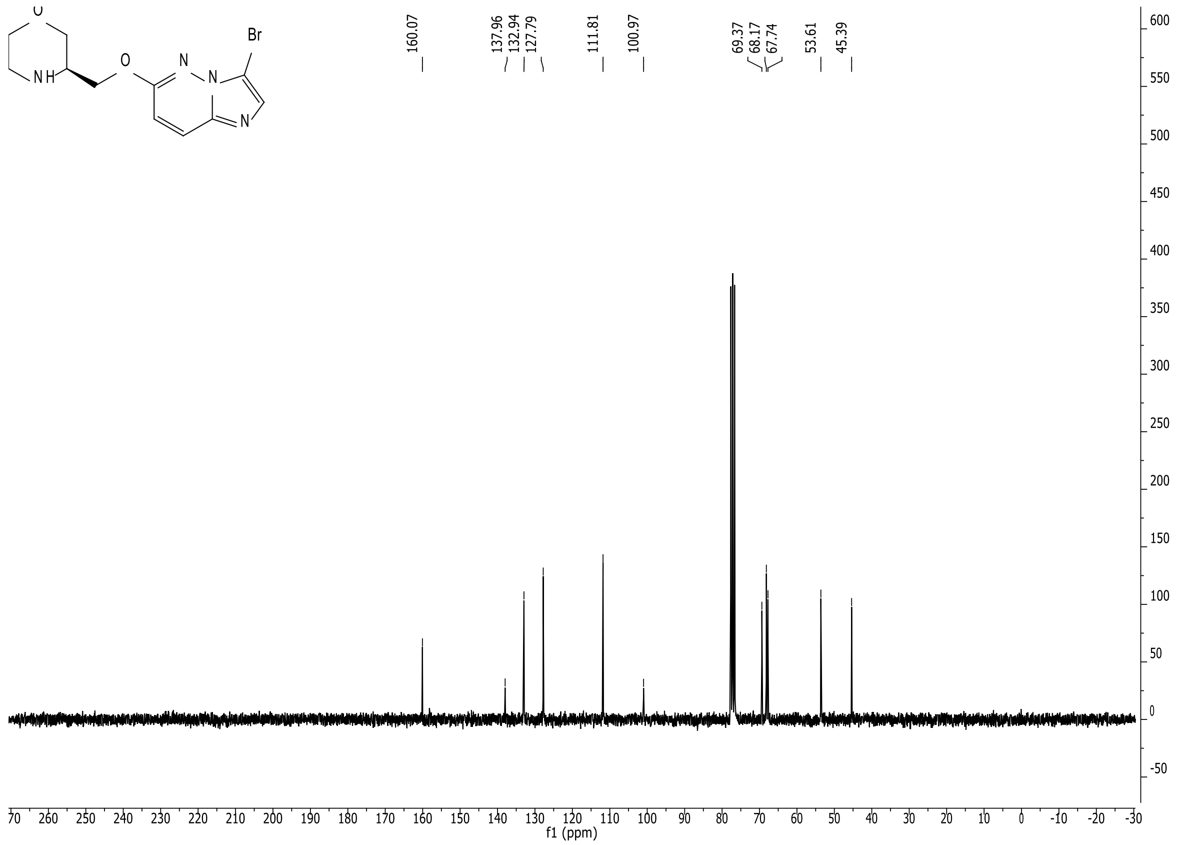
Br
O
N
NH
160.07
137.96
132.94
127.79
111.81
100.97
69.37
68.17
67.74
53.61
45.39
f1 (ppm)

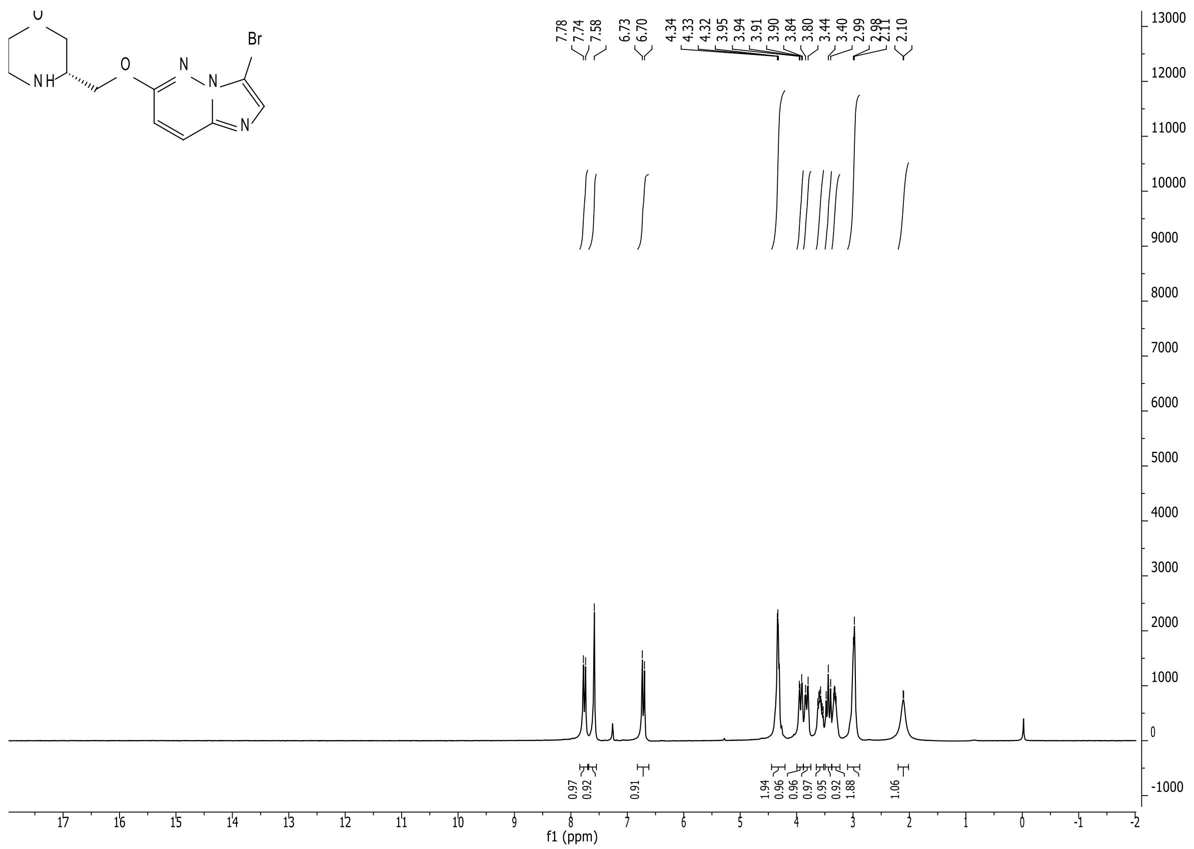

Br
O
N
N
NH
7.78
7.74
7.58
6.73
6.70
4.34
4.33
4.32
3.95
3.94
3.91
3.90
3.84
3.80
3.44
3.40
2.99
2.98
2.11
2.10
0.97
0.92
0.91
1.94
0.96
0.96
0.97
0.95
0.92
1.88
1.06
f1 (ppm)

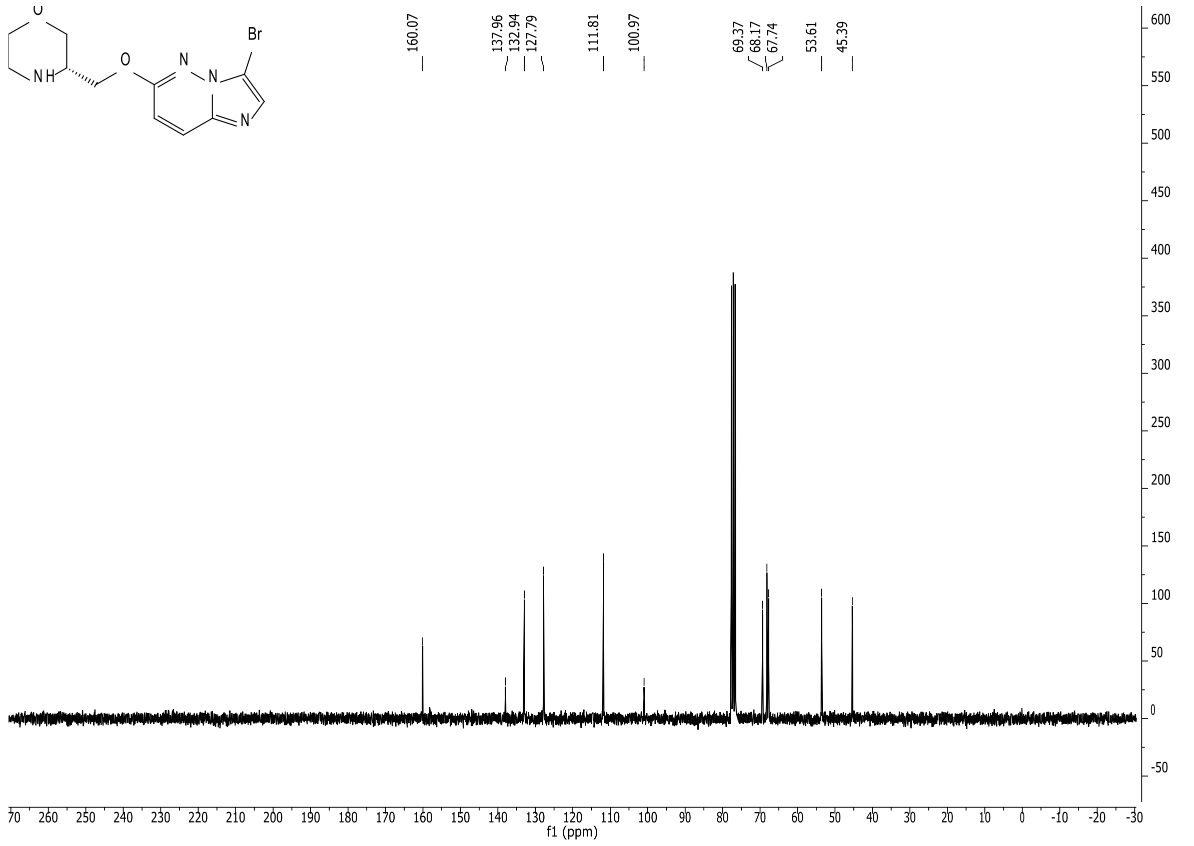

Br
O
N
N
NH
160.07
137.96
132.94
127.79
111.81
100.97
69.37
68.17
67.74
53.61
45.39
f1 (ppm)

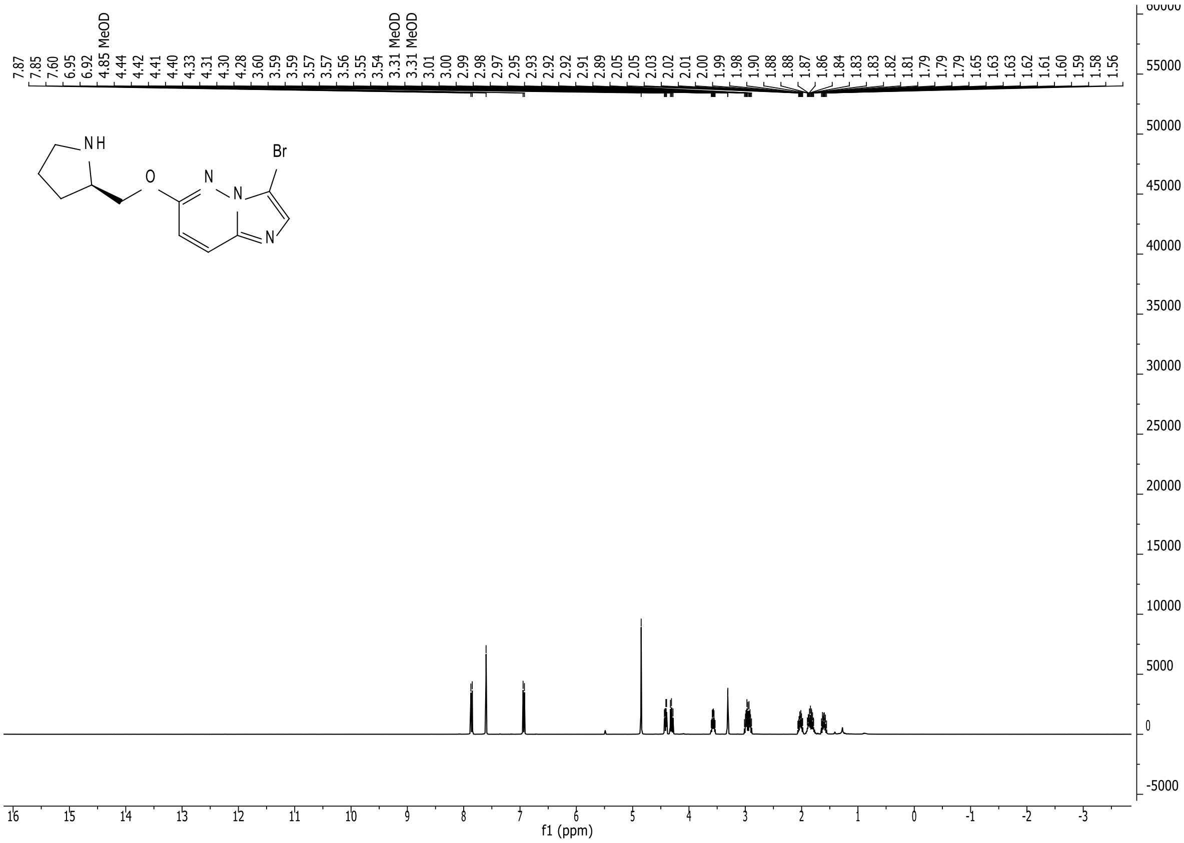

7.87 7.85 7.60 6.95 6.92 4.85 MeOD 4.44 4.42 4.41 4.40 4.33 4.31 4.30 4.28 3.60 3.59 3.59 3.57 3.57 3.56 3.55 3.54 3.31 MeOD 3.31 MeOD 3.01 3.00 2.99 2.98 2.97 2.95 2.93 2.92 2.92 2.91 2.89 2.05 2.05 2.03 2.02 2.01 2.00 1.99 1.98 1.90 1.88 1.88 1.87 1.86 1.84 1.83 1.83 1.82 1.81 1.79 1.79 1.65 1.63 1.63 1.62 1.61 1.60 1.59 1.58 1.56
NH
O
N
N
Br
N
f1 (ppm)

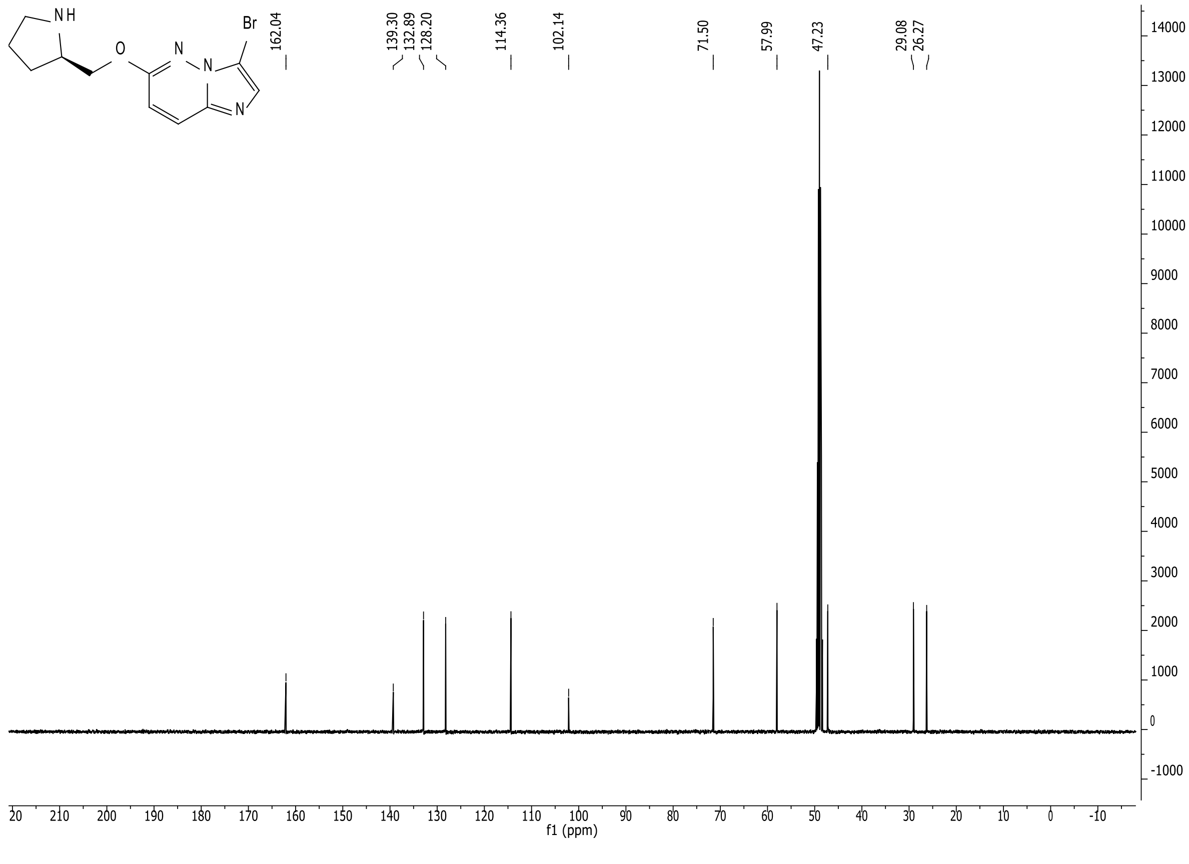

NH
O
N
N
Br
N
162.04
139.30 132.89 128.20
114.36
102.14
71.50
57.99
47.23
29.08 26.27
f1 (ppm)

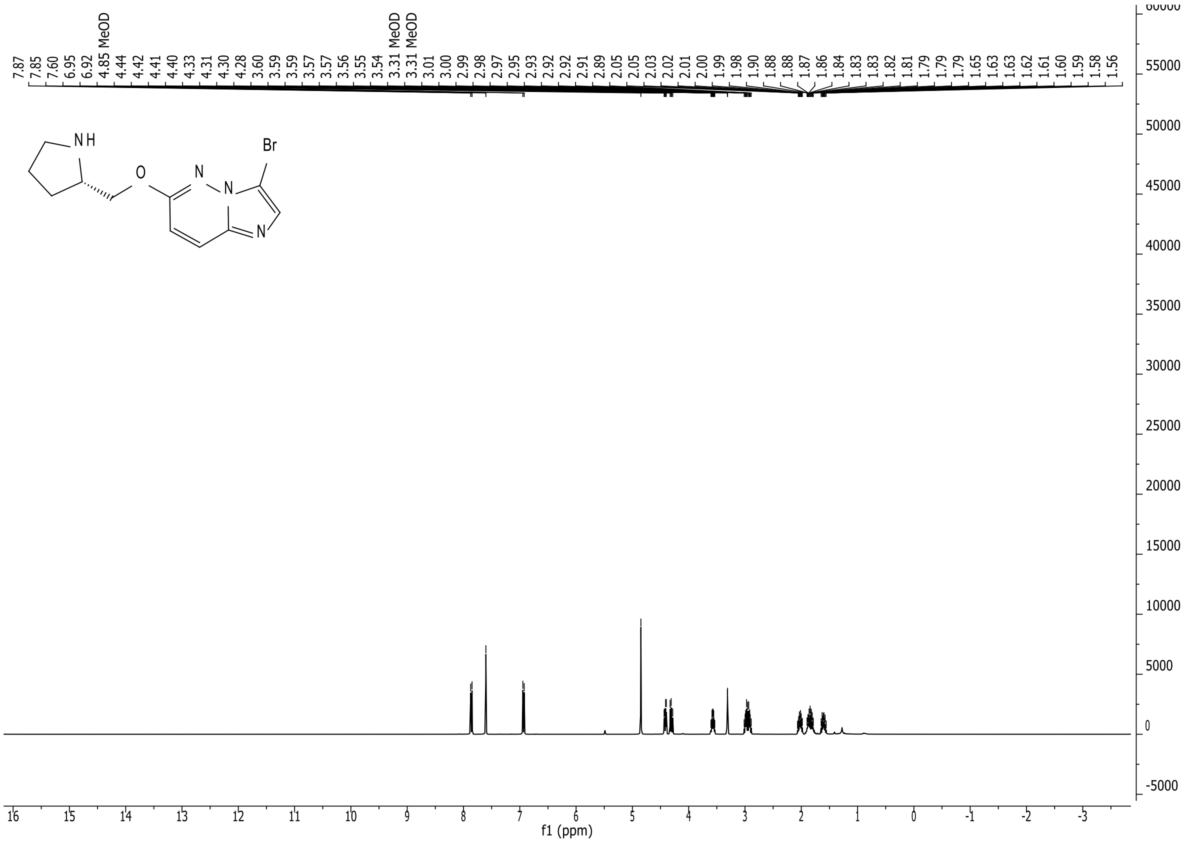
7.87
7.85
7.60
6.95
6.92
4.85 MeOD
4.44
4.42
4.41
4.40
4.33
4.31
4.30
4.28
3.60
3.59
3.59
3.57
3.57
3.56
3.55
3.54
3.31 MeOD
3.31 MeOD
3.01
3.00
2.99
2.98
2.97
2.95
2.93
2.92
2.92
2.91
2.89
2.05
2.05
2.03
2.02
2.01
2.00
1.99
1.98
1.90
1.88
1.88
1.87
1.86
1.84
1.83
1.83
1.82
1.81
1.79
1.79
1.65
1.63
1.63
1.62
1.61
1.60
1.59
1.58
1.56
NH
O
N
N
Br
N
f1 (ppm)

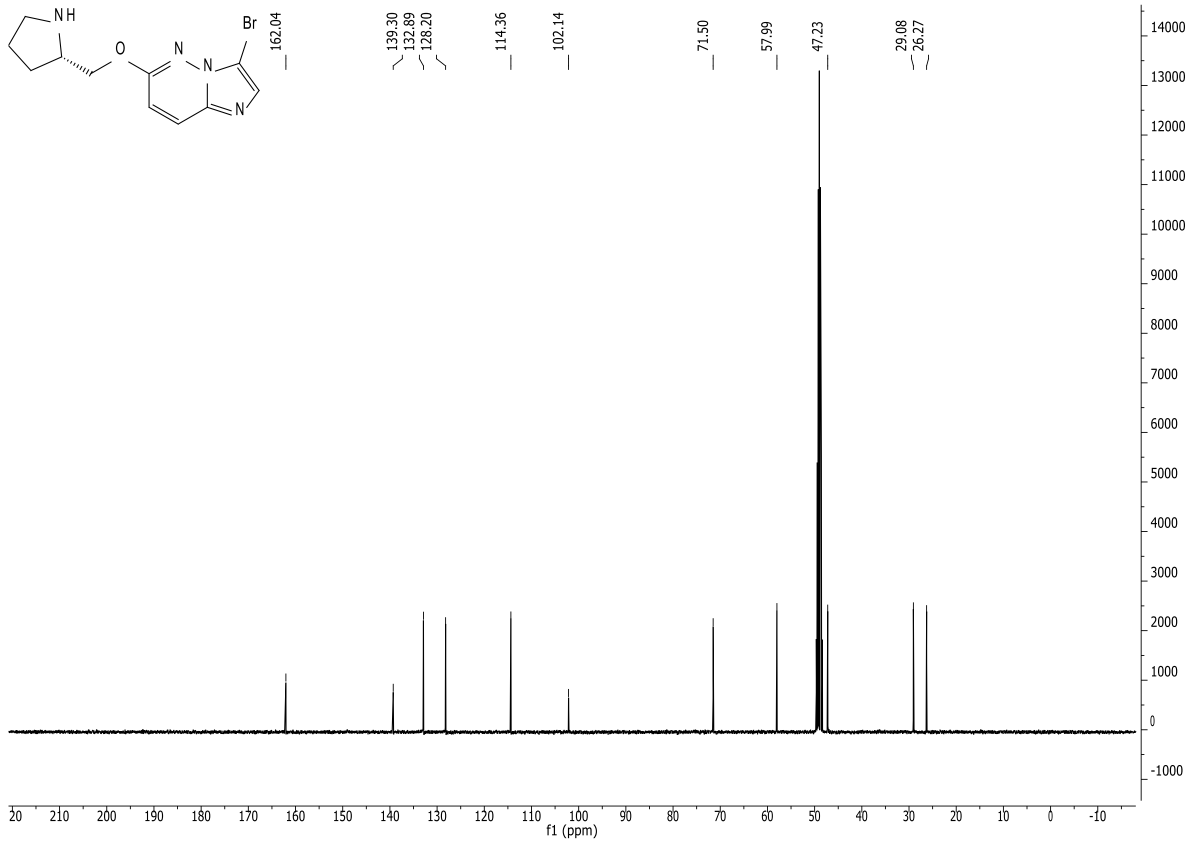
NH
O
N
N
Br
N
162.04
139.30
132.89
128.20
114.36
102.14
71.50
57.99
47.23
29.08
26.27
f1 (ppm)

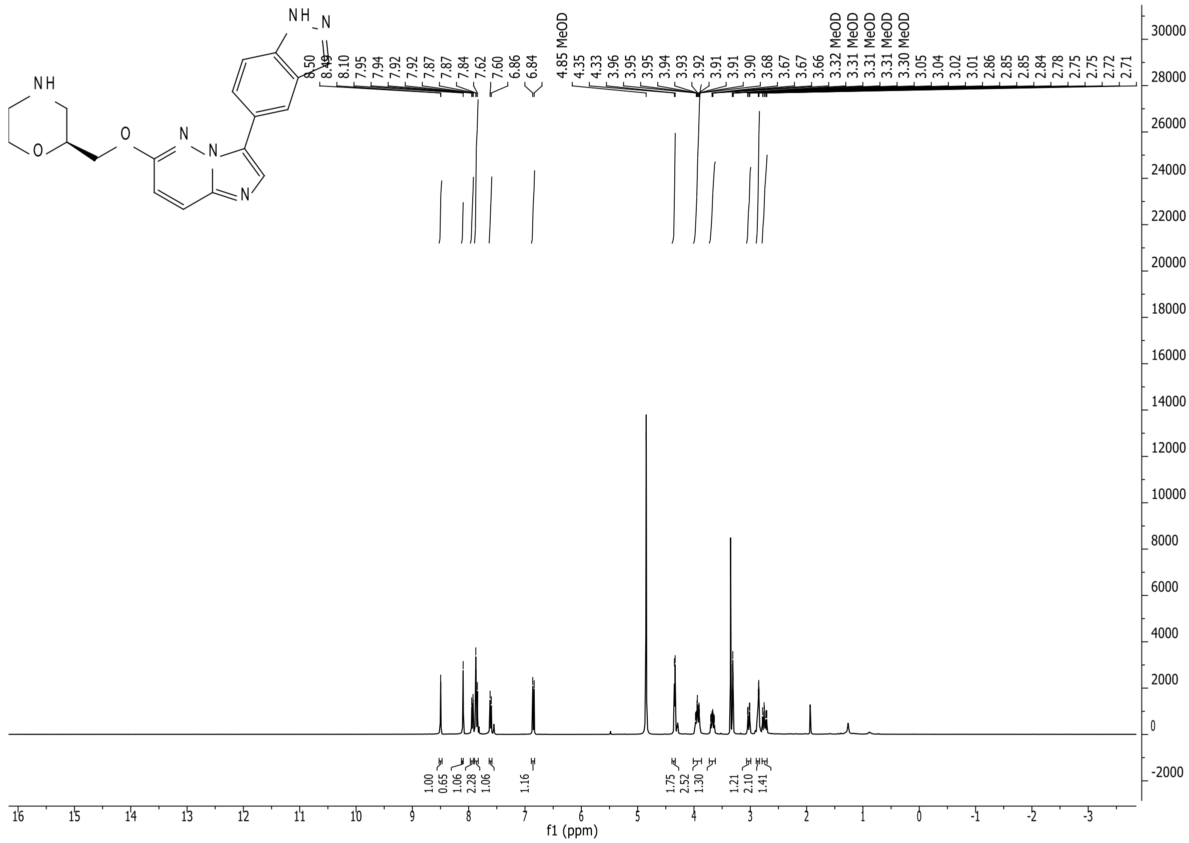
NH
NH
N
O
O
N
N
N
8.50
8.49
8.10
7.95
7.94
7.92
7.92
7.87
7.87
7.84
7.62
7.60
6.86
6.84
4.85 MeOD
4.35
4.33
3.96
3.95
3.95
3.94
3.93
3.92
3.91
3.91
3.90
3.68
3.67
3.66
3.32 MeOD
3.31 MeOD
3.31 MeOD
3.31 MeOD
3.30 MeOD
3.05
3.04
3.02
3.01
2.86
2.85
2.85
2.84
2.78
2.75
2.72
2.71
1.00
0.65
1.06
2.28
1.06
1.16
1.75
2.52
1.30
1.21
2.10
1.41
f1 (ppm)

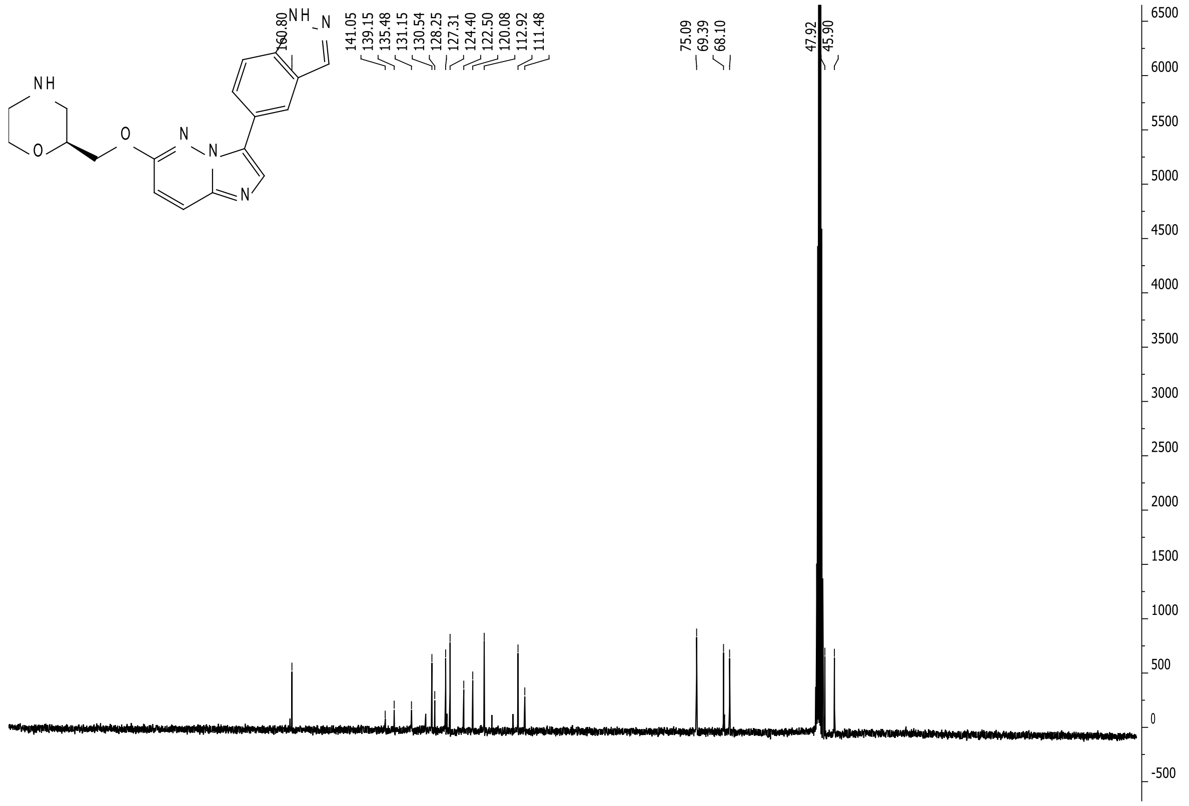
NH
NH
N
O
O
N
N
N
160.80
141.05
139.15
135.48
131.15
130.54
128.25
127.31
124.40
122.50
120.08
112.92
111.48
75.09
69.39
68.10
47.92
45.90
f1 (ppm)

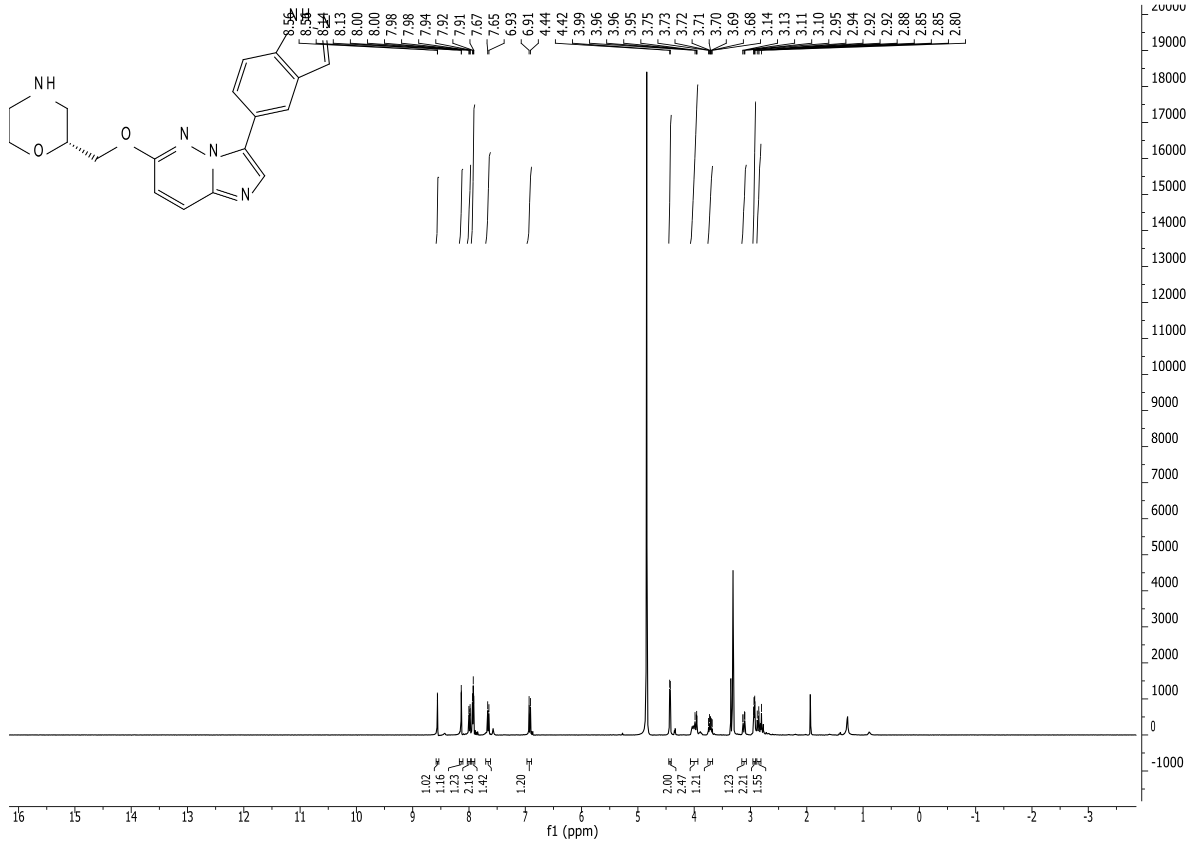

NH
O
O
N
N
N
NH
N
8.56
8.13
8.00
7.98
7.94
7.92
7.91
7.67
7.65
6.93
6.91
4.44
4.42
3.99
3.96
3.96
3.95
3.75
3.73
3.72
3.71
3.70
3.69
3.68
3.14
3.13
3.11
3.10
2.95
2.94
2.92
2.92
2.88
2.85
2.85
2.80
1.02
1.16
1.23
2.16
1.42
1.20
2.00
2.47
1.21
1.23
2.21
1.55
f1 (ppm)

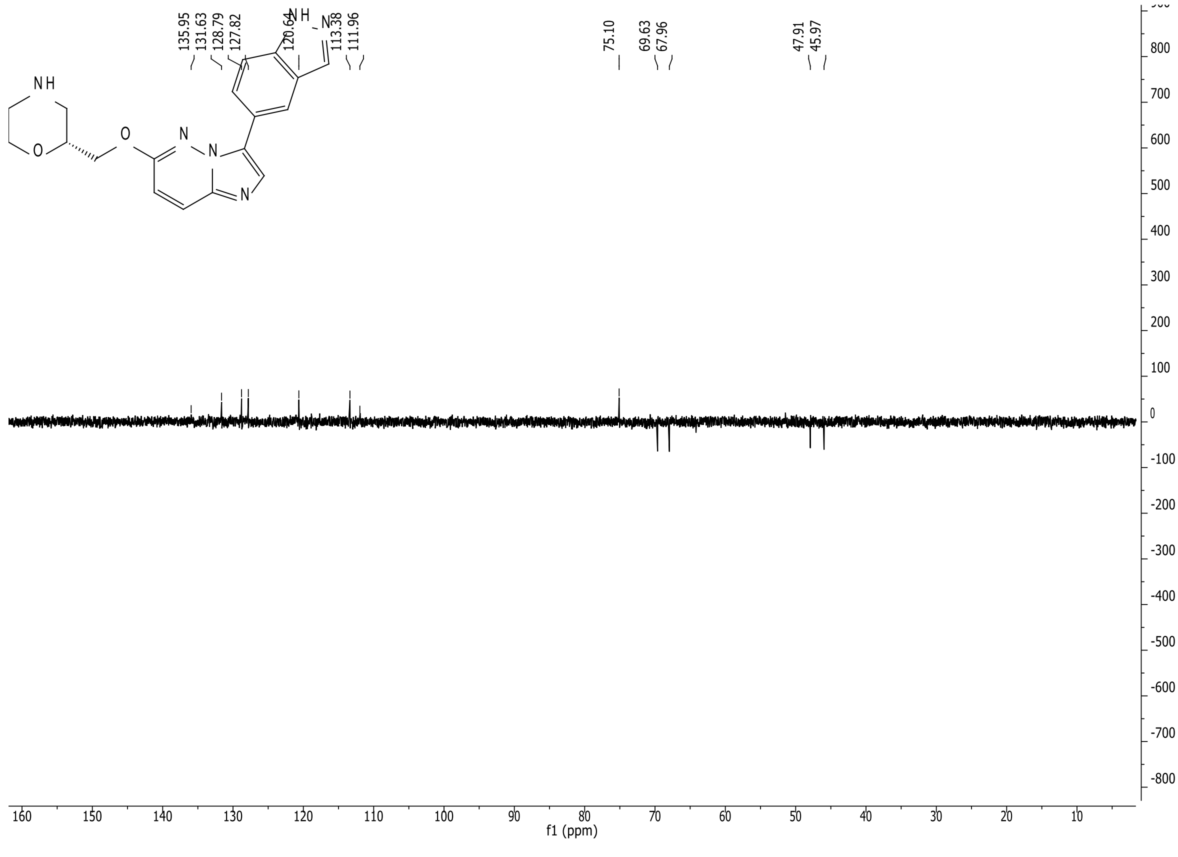

NH
O
O
N
N
N
NH
N
135.95
131.63
128.79
127.82
120.64
113.38
111.96
75.10
69.63
67.96
47.91
45.97
f1 (ppm)

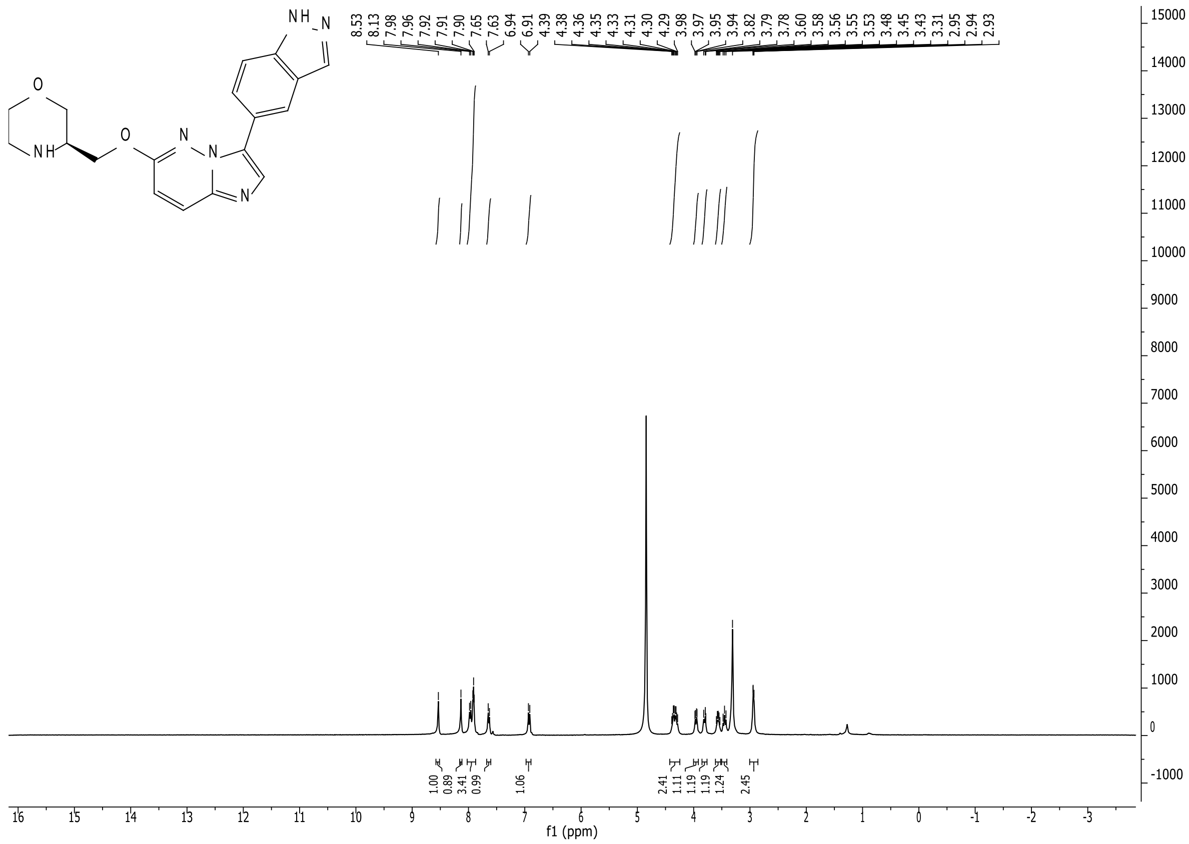
8.53 8.13 7.98 7.96 7.92 7.91 7.90 7.65 7.63 6.94 6.91 4.39 4.38 4.36 4.35 4.33 4.31 4.30 4.29 3.98 3.97 3.95 3.94 3.82 3.79 3.78 3.60 3.58 3.56 3.55 3.53 3.48 3.45 3.43 3.31 2.95 2.94 2.93
1.00 0.89 3.41 0.99 1.06 2.41 1.11 1.19 1.19 1.24 2.45
f1 (ppm)

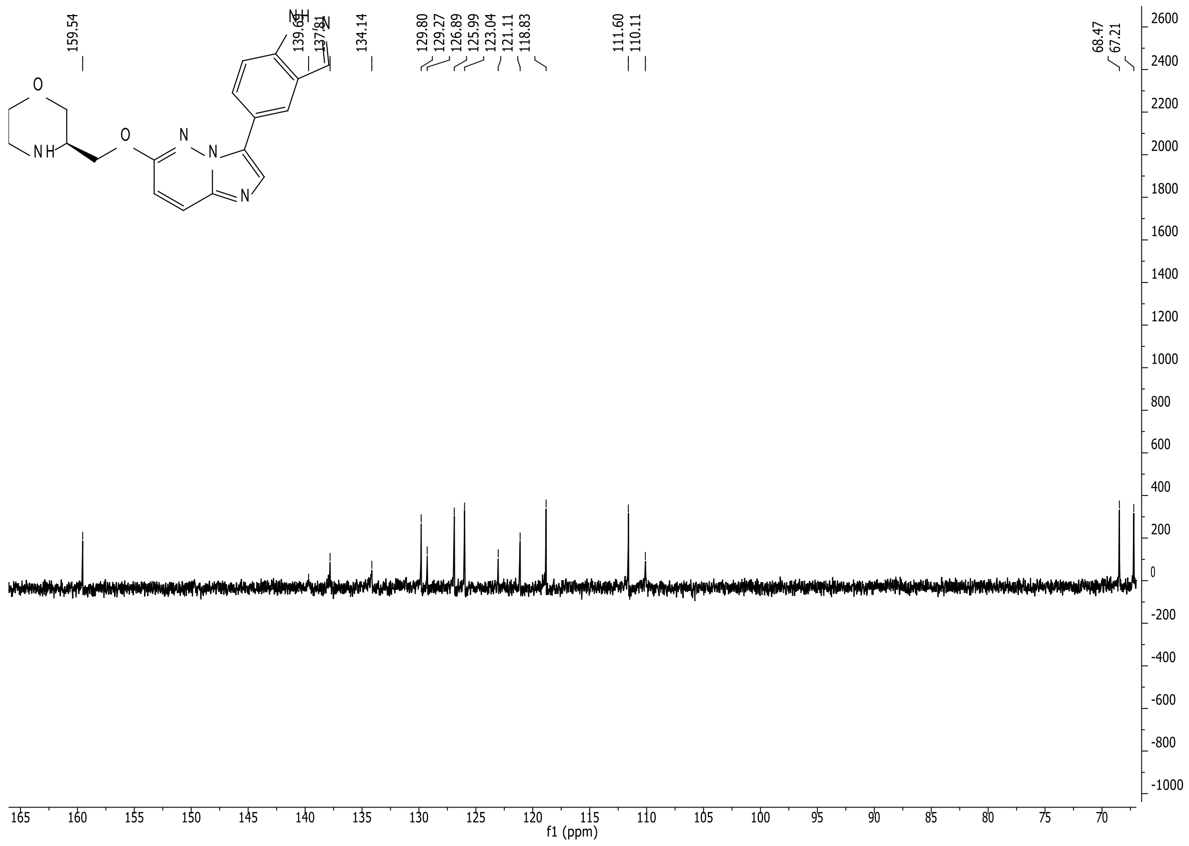
159.54 139.65 137.81 134.14 129.80 129.27 126.89 125.99 123.04 121.11 118.83 111.60 110.11 68.47 67.21
f1 (ppm)

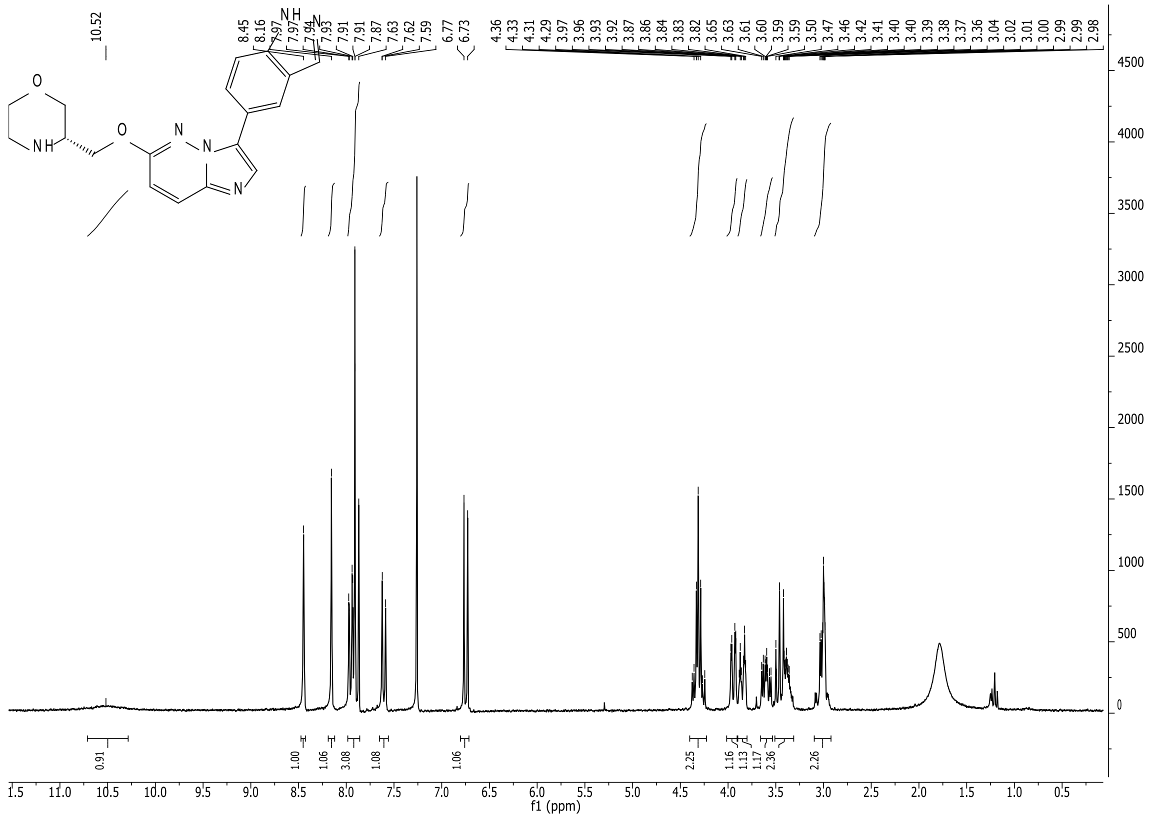
10.52
8.45
8.16
7.97
7.97
7.94
7.93
7.91
7.91
7.87
7.63
7.62
7.59
6.77
6.73
4.36
4.33
4.31
4.29
3.97
3.96
3.93
3.92
3.87
3.86
3.84
3.83
3.82
3.65
3.63
3.61
3.60
3.59
3.59
3.50
3.47
3.46
3.42
3.41
3.40
3.40
3.39
3.38
3.37
3.36
3.04
3.02
3.01
3.00
2.99
2.99
2.98
O
NH
O
N
N
N
NH
N
0.91
1.00
1.06
3.08
1.08
1.06
2.25
1.16
1.13
1.17
2.36
2.26
f1 (ppm)

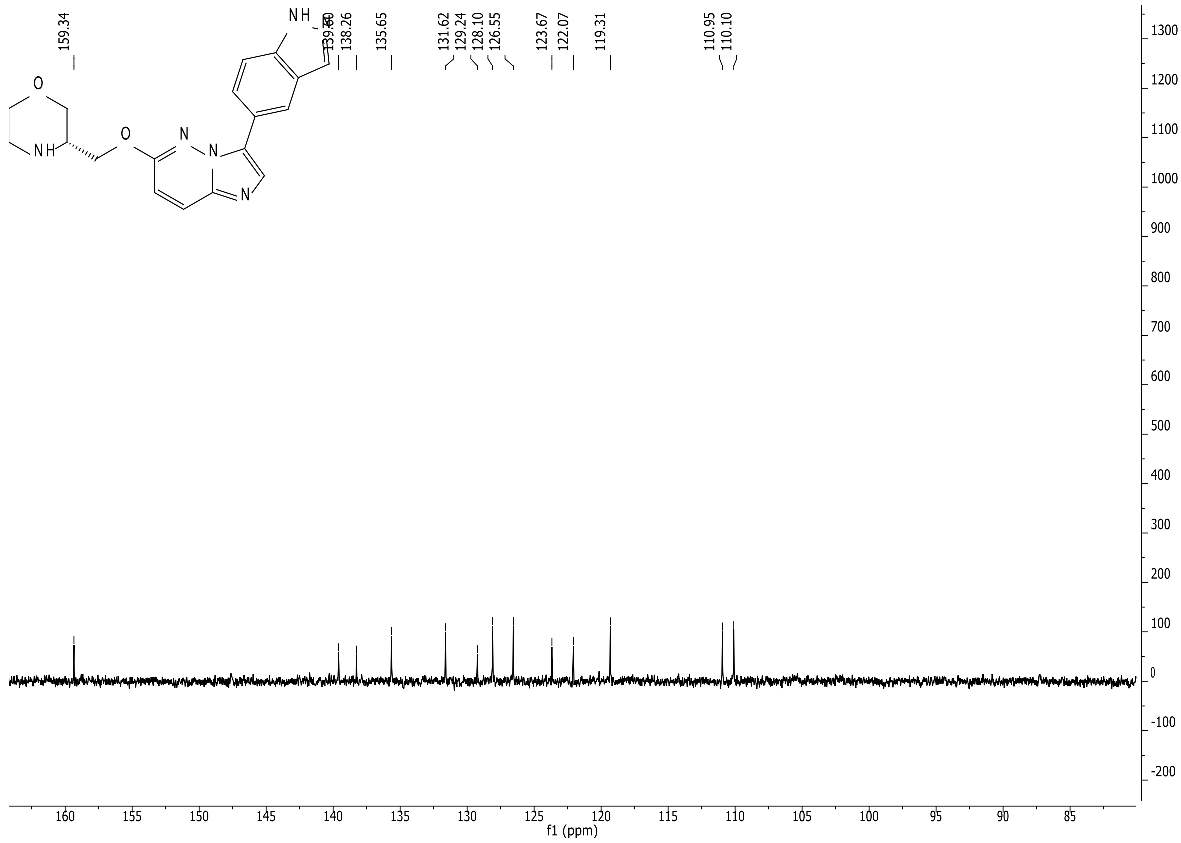
159.34
138.26
135.65
131.62
129.24
128.10
126.55
123.67
122.07
119.31
110.95
110.10
O
NH
O
N
N
N
NH
N
f1 (ppm)

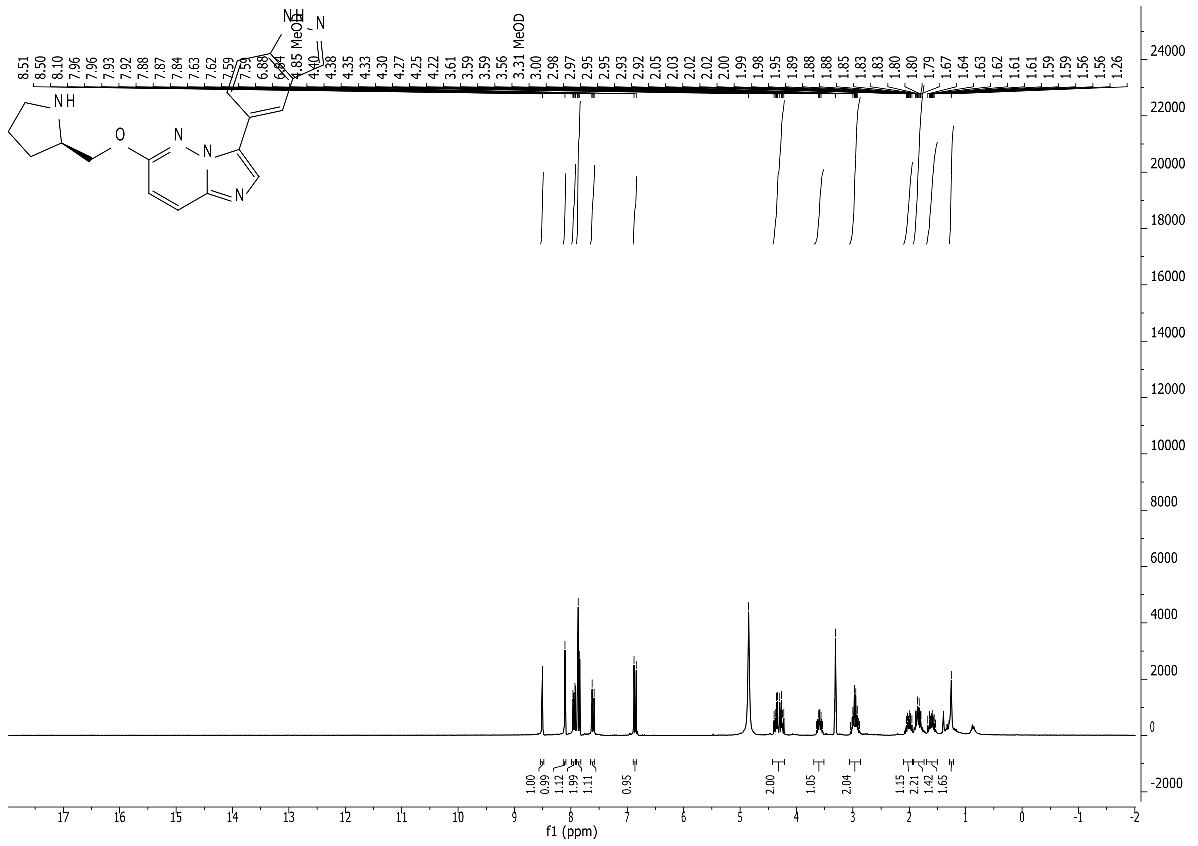
8.51
8.50
8.10
7.96
7.96
7.93
7.92
7.88
7.87
7.84
7.63
7.62
7.59
7.59
6.88
6.84
4.85 MeOH
4.40
4.38
4.35
4.33
4.30
4.27
4.25
4.22
3.61
3.59
3.59
3.56
3.31 MeOD
3.00
2.98
2.97
2.95
2.95
2.93
2.92
2.05
2.03
2.03
2.02
2.00
1.99
1.98
1.95
1.89
1.88
1.88
1.85
1.83
1.83
1.80
1.80
1.79
1.67
1.64
1.63
1.62
1.61
1.61
1.59
1.56
1.56
1.26
NH
O
N
N
N
NH
N
1.00
0.99
1.12
1.99
1.11
0.95
2.00
1.05
2.04
1.15
2.21
1.42
1.65
f1 (ppm)

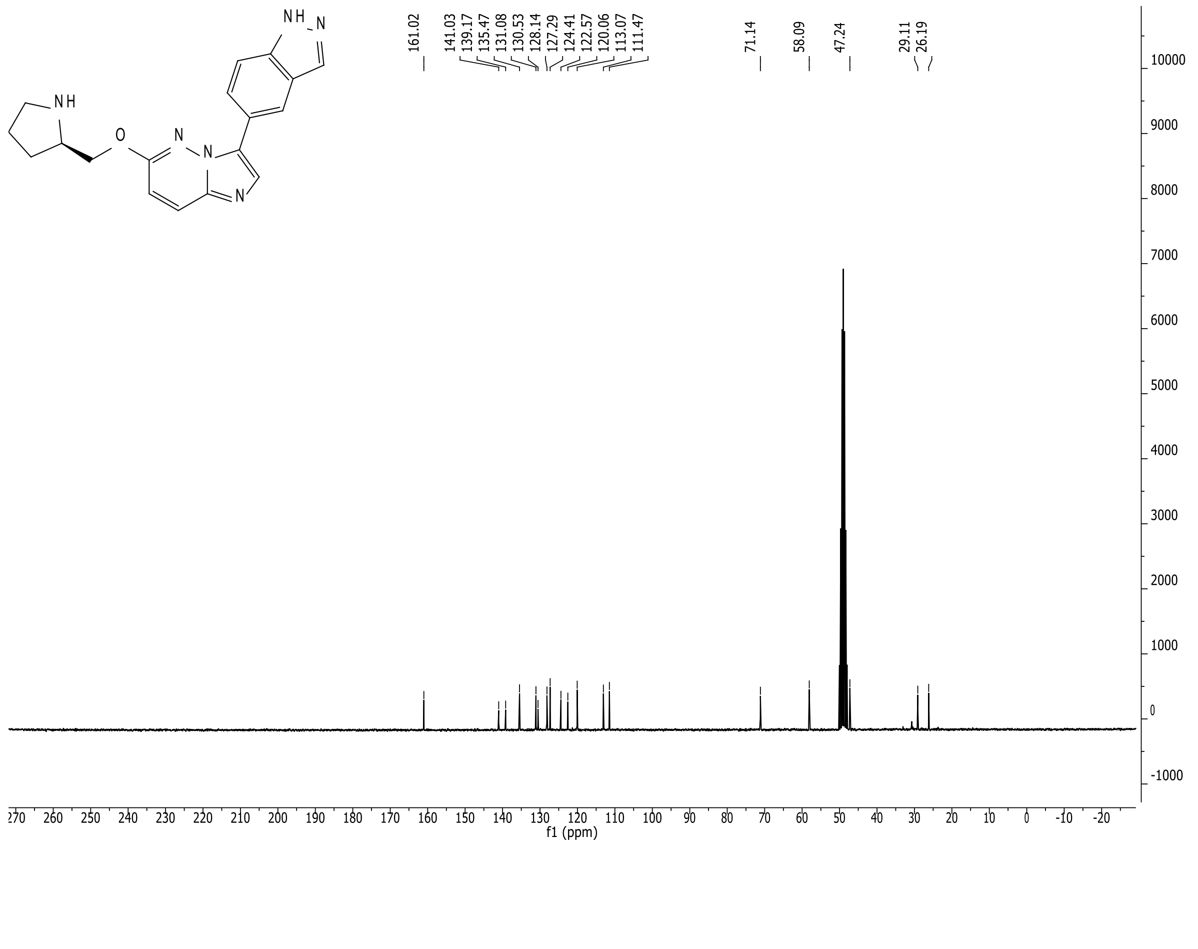
161.02
141.03
139.17
135.47
131.08
130.53
128.14
127.29
124.41
122.57
120.06
113.07
111.47
71.14
58.09
47.24
29.11
26.19
NH
O
N
N
N
NH
N
f1 (ppm)

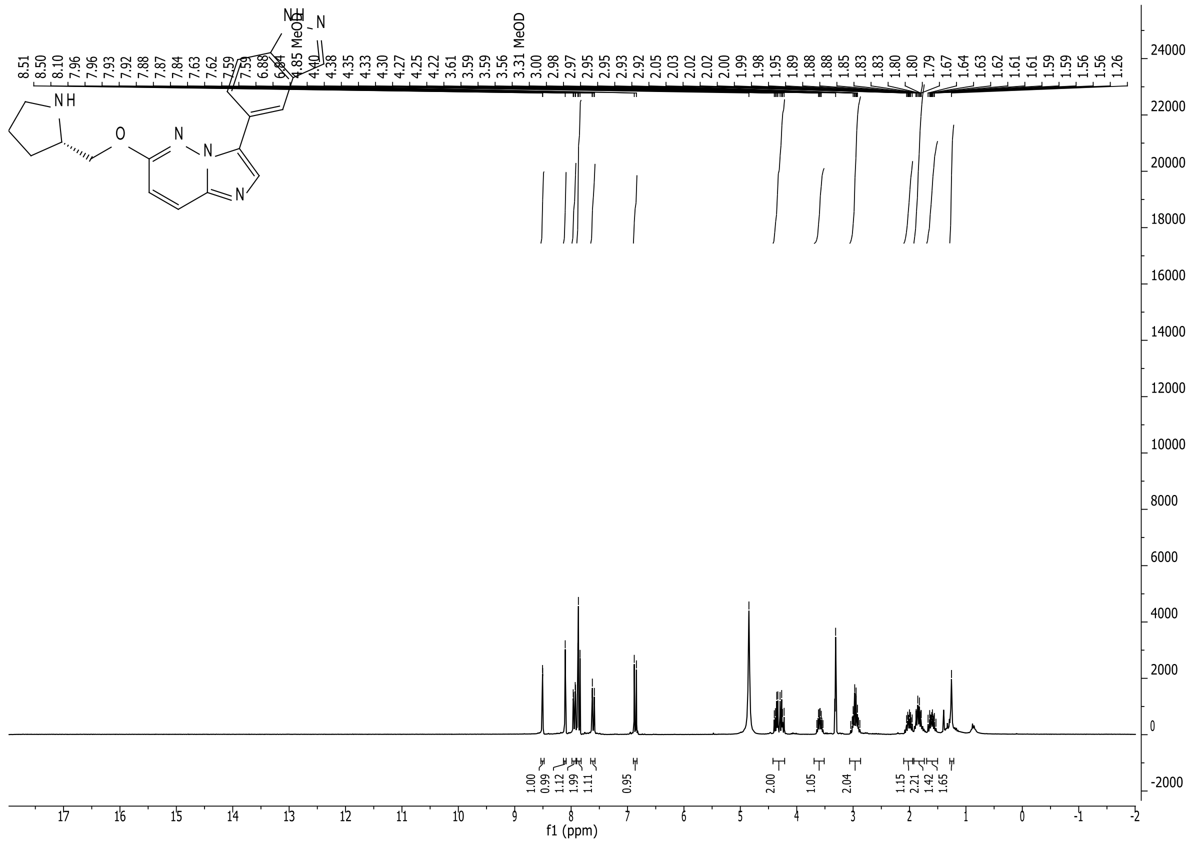
8.51 8.50 8.10 7.96 7.96 7.93 7.92 7.88 7.87 7.84 7.63 7.62 7.59 7.59 6.88 6.84 4.85 MeOD 4.40 4.38 4.35 4.33 4.30 4.27 4.25 4.22 3.61 3.59 3.59 3.56 3.31 MeOD 3.00 2.98 2.97 2.95 2.95 2.93 2.92 2.05 2.03 2.02 2.02 2.00 1.99 1.98 1.95 1.89 1.88 1.88 1.85 1.83 1.83 1.80 1.80 1.79 1.67 1.64 1.63 1.62 1.61 1.61 1.59 1.56 1.26
NH
O
N
N
N
NH
N
1.00 0.99 1.12 1.99 1.11 0.95 2.00 1.05 2.04 1.15 2.21 1.42 1.65
f1 (ppm)

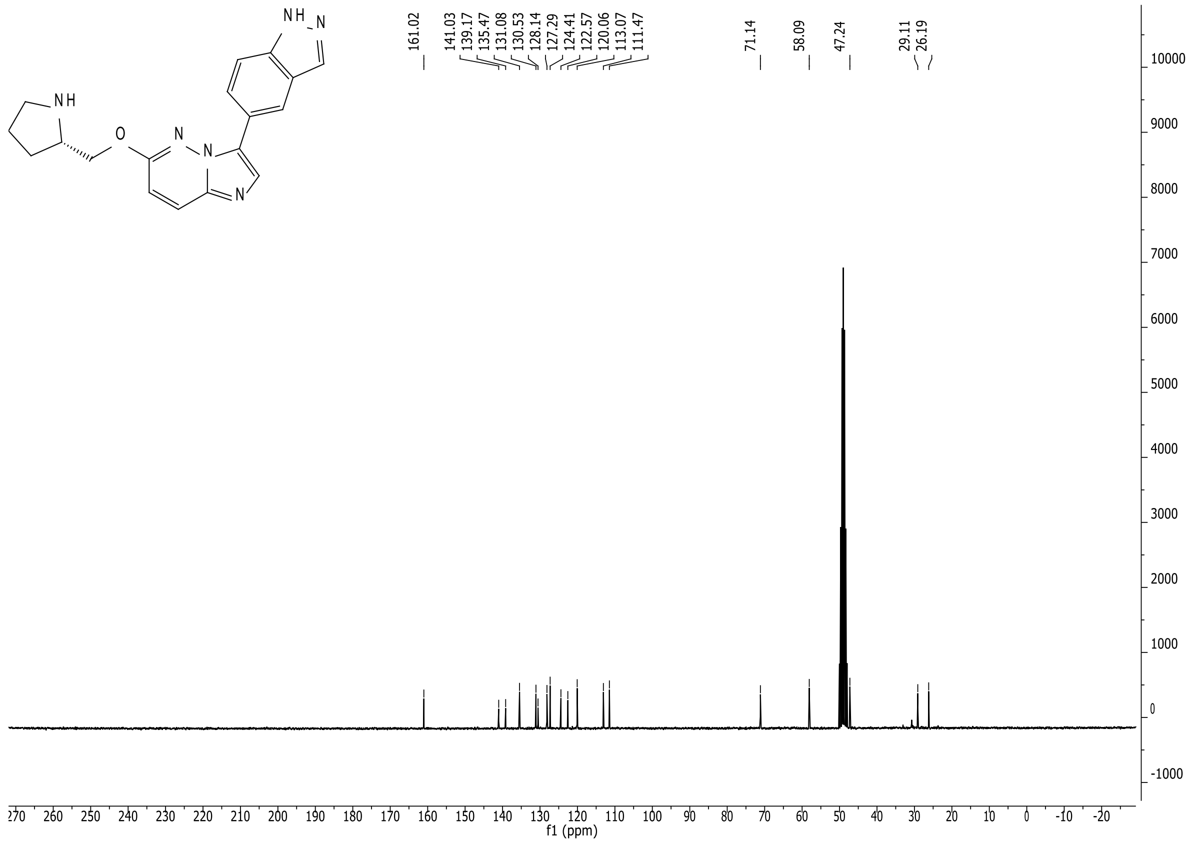
161.02 141.03 139.17 135.47 131.08 130.53 128.14 127.29 124.41 122.57 120.06 113.07 111.47 71.14 58.09 47.24 29.11 26.19
NH
N
NH
O
N
N
N
f1 (ppm)

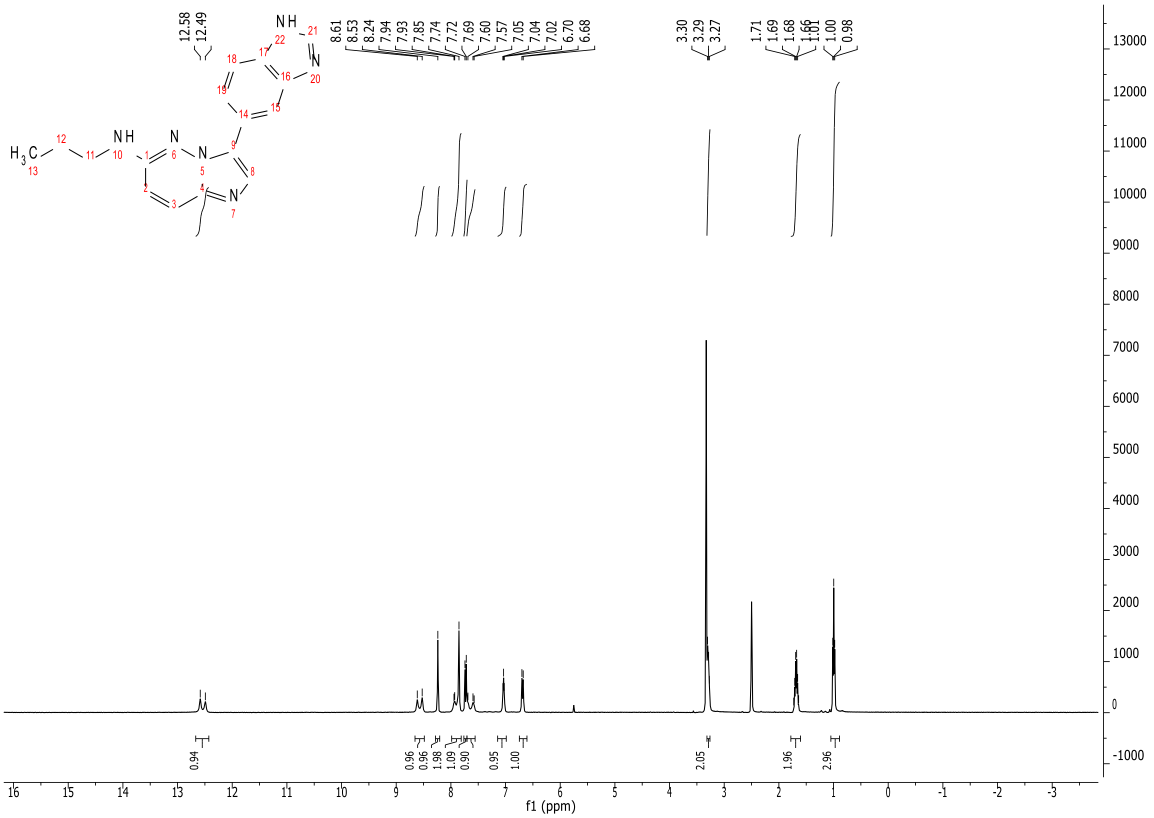
12.58
12.49
8.61
8.53
8.24
7.94
7.93
7.85
7.74
7.72
7.69
7.60
7.57
7.05
7.04
7.02
6.70
6.68
3.30
3.29
3.27
1.71
1.69
1.68
1.66
1.01
1.00
0.98
0.94
0.96
0.96
1.98
1.09
0.90
0.95
1.00
2.05
1.96
2.96
f1 (ppm)

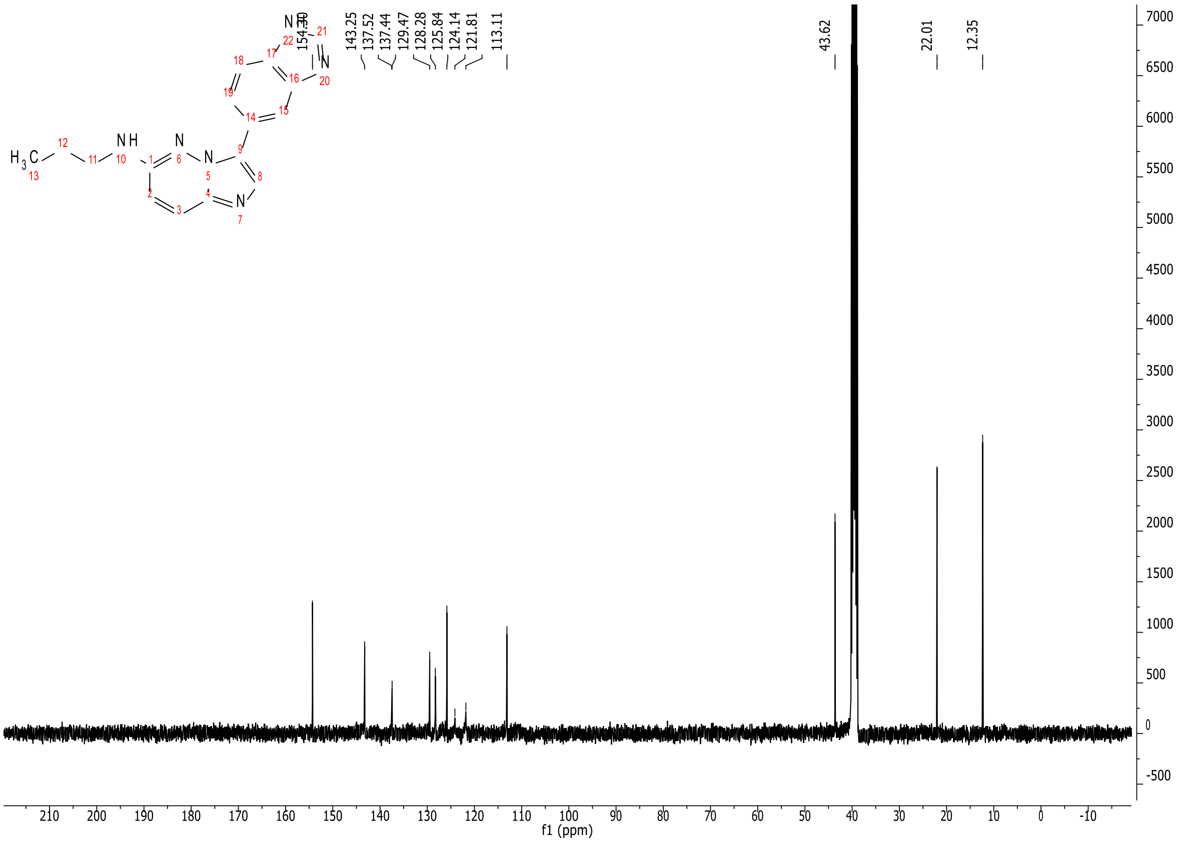
154.30
143.25
137.52
137.44
129.47
128.28
125.84
124.14
121.81
113.11
43.62
22.01
12.35
f1 (ppm)

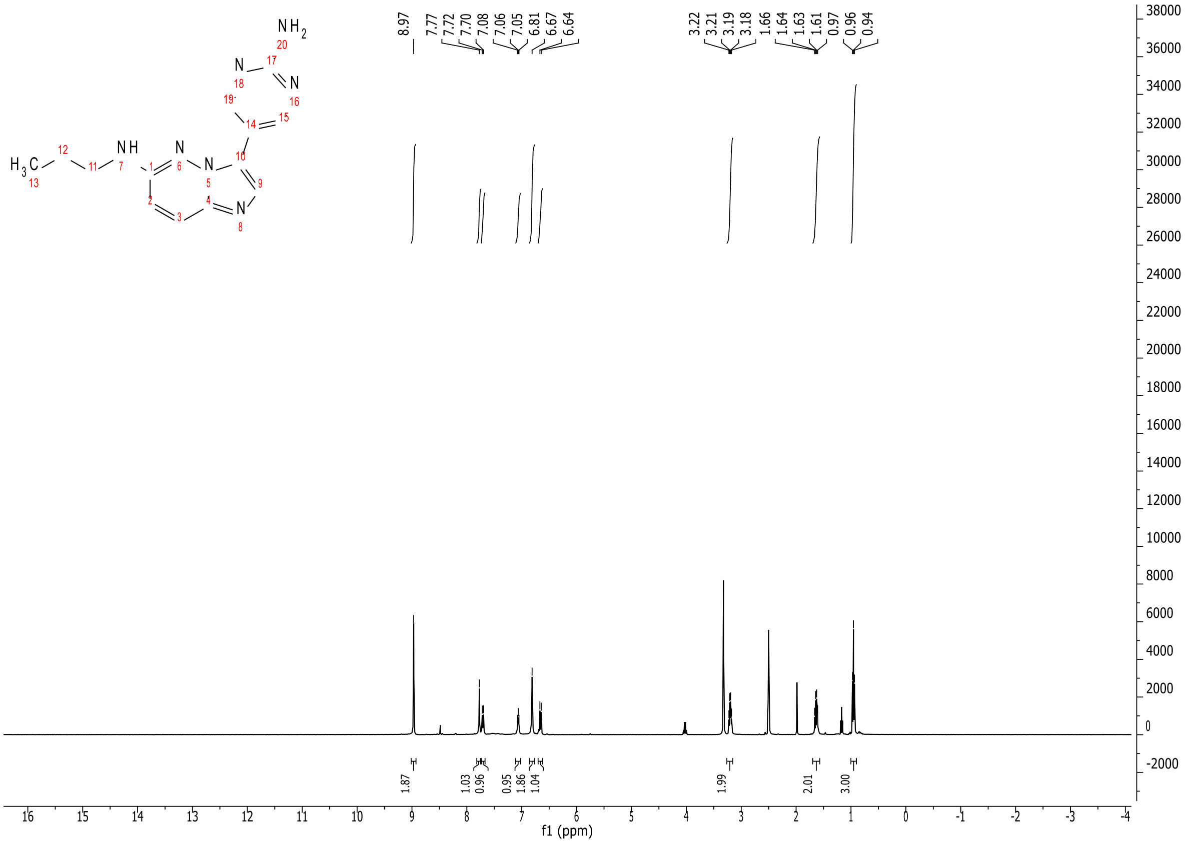

8.97
7.77
7.72
7.70
7.08
7.06
7.05
6.81
6.67
6.64
3.22
3.21
3.19
3.18
1.66
1.64
1.63
1.61
0.97
0.96
0.94
1.87
1.03
0.96
0.95
1.86
1.04
1.99
2.01
3.00
f1 (ppm)

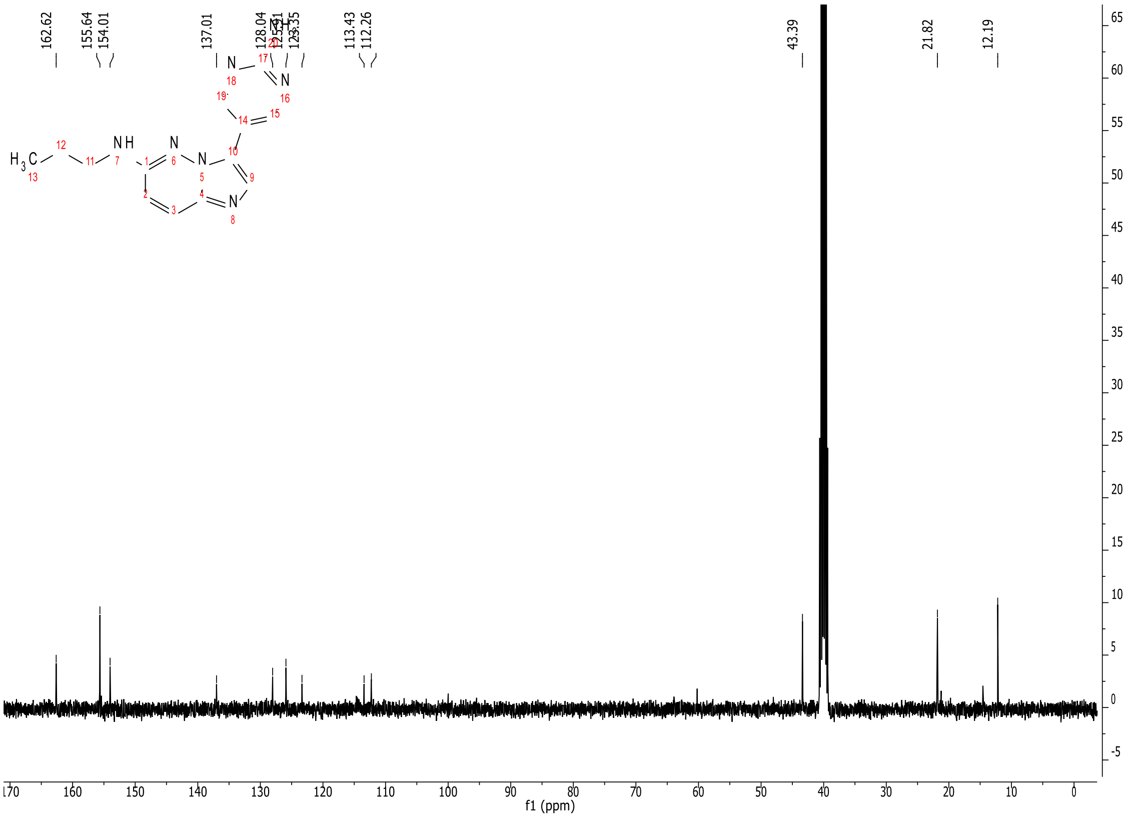

162.62
155.64
154.01
137.01
128.04
123.35
113.43
112.26
43.39
21.82
12.19
f1 (ppm)

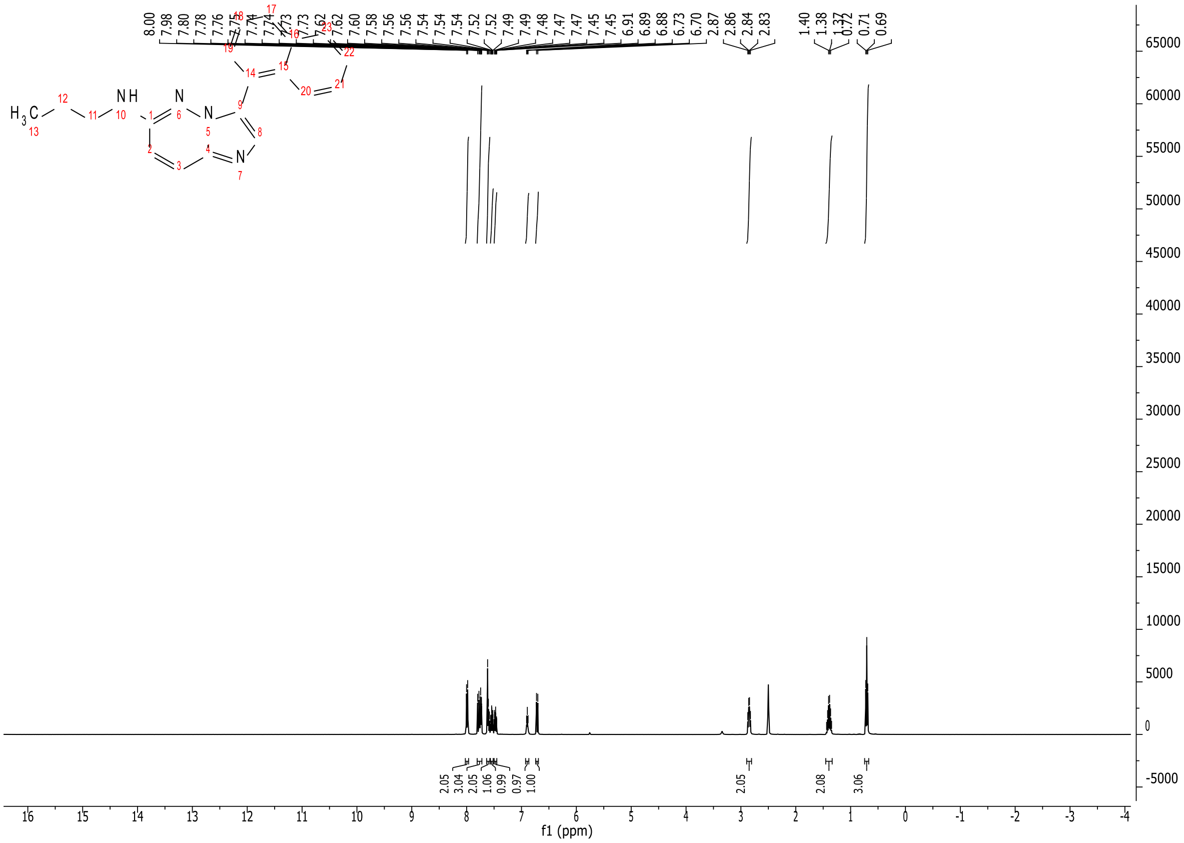

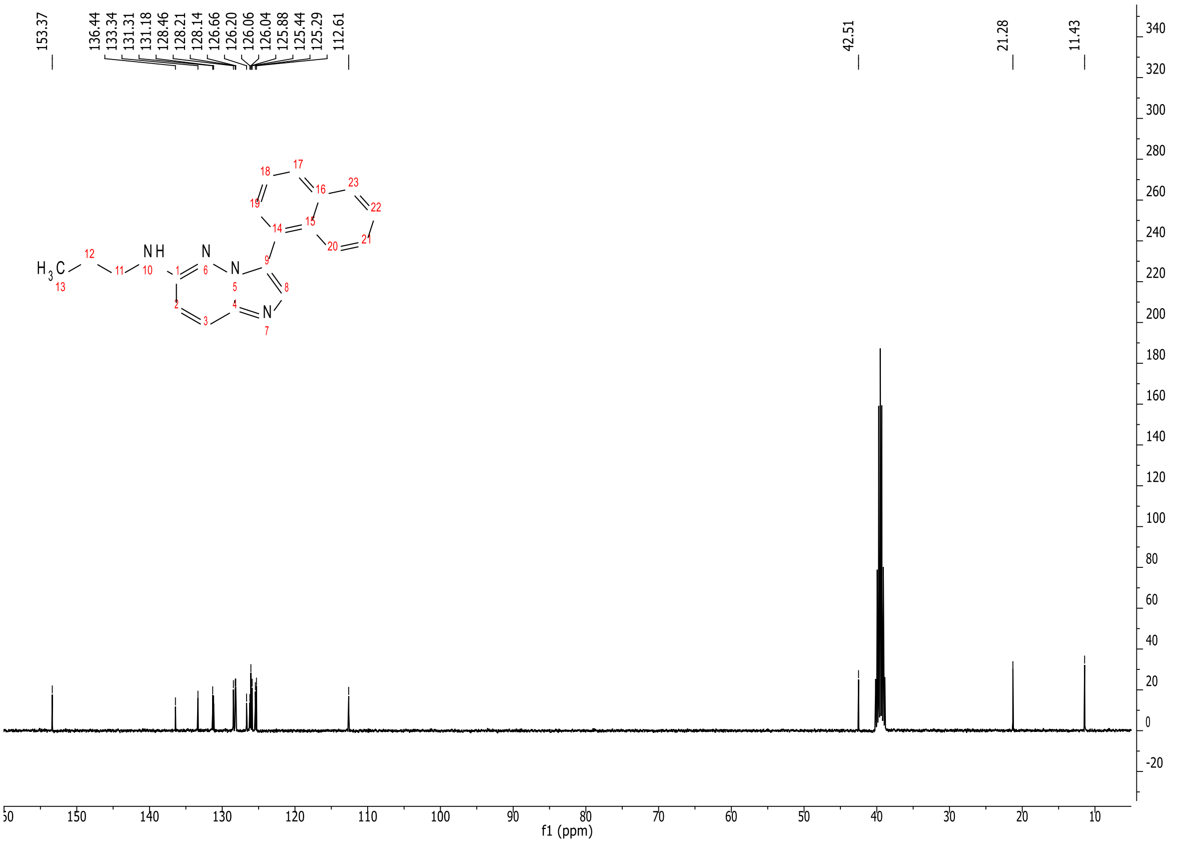

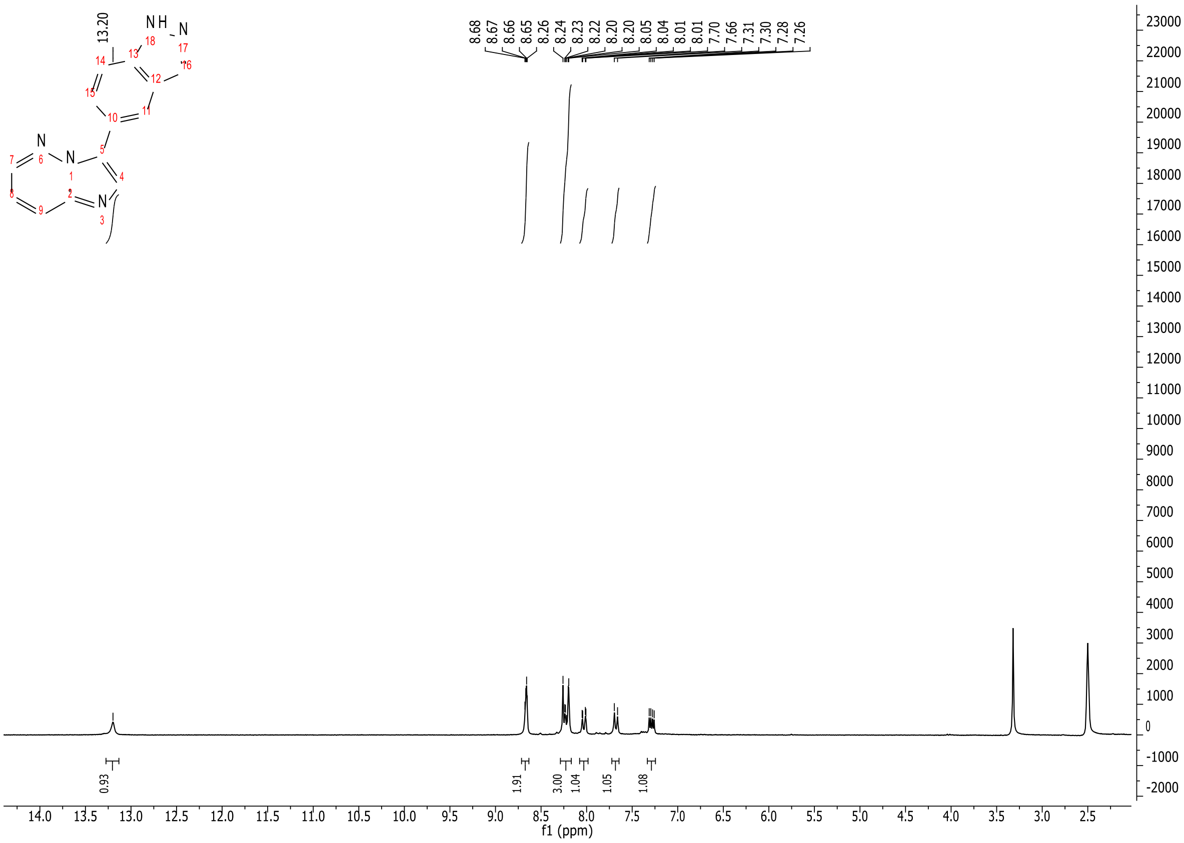
13.20
8.68
8.67
8.66
8.65
8.26
8.24
8.23
8.22
8.20
8.20
8.05
8.04
8.01
8.01
7.70
7.66
7.31
7.30
7.28
7.26
0.93
1.91
3.00
1.04
1.05
1.08
f1 (ppm)

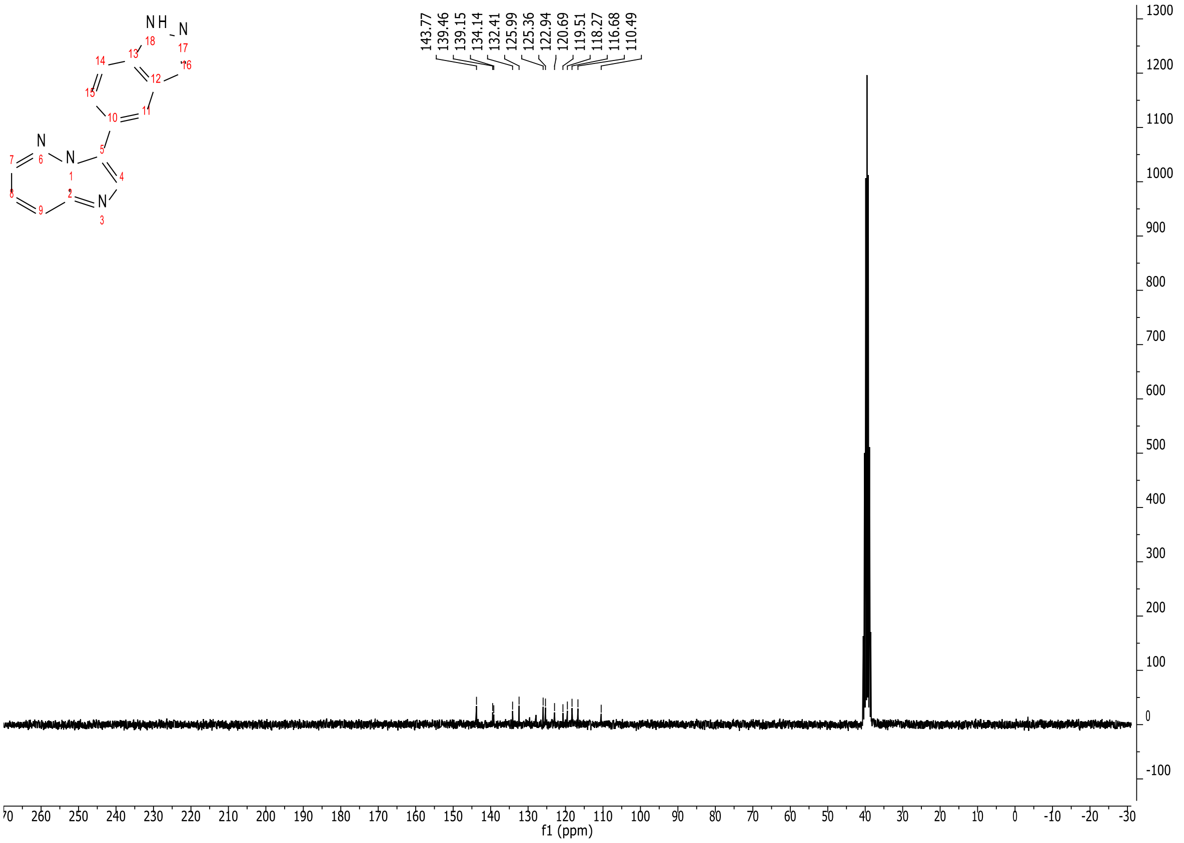
143.77
139.46
139.15
134.14
132.41
125.99
125.36
122.94
120.69
119.51
118.27
116.68
110.49
f1 (ppm)
